# Supplementary material for: Reconstitution of early paclitaxel biosynthetic network
Source: Nat Commun. 2024 Feb 15;15:1419. doi: 10.1038/s41467-024-45574-8 (PMC10869802; doi:10.1038/s41467-024-45574-8)
Supplement: Supplementary file 1 — Supplementary information [file 41467_2024_45574_MOESM1_ESM.pdf]

Supplementary Information

Reconstitution of Early Paclitaxel Biosynthetic Network

Jack Chun-Ting Liu[1], Ricardo De La Peña[2], Christian Tocol[2], Elizabeth S. Sattely[2,3*]

[1]Department of Chemistry, Stanford University, Stanford, CA, USA. [2]Department of Chemical Engineering, Stanford University, Stanford, CA, USA. [3]Howard Hughes Medical Institute, Stanford University, Stanford, CA, USA

*correspondence: sattely@stanford.edu

Table of Contents

Supplementary Figures **2**
Supplementary Tables **56**
Supplementary References **78**

Supplementary Figures

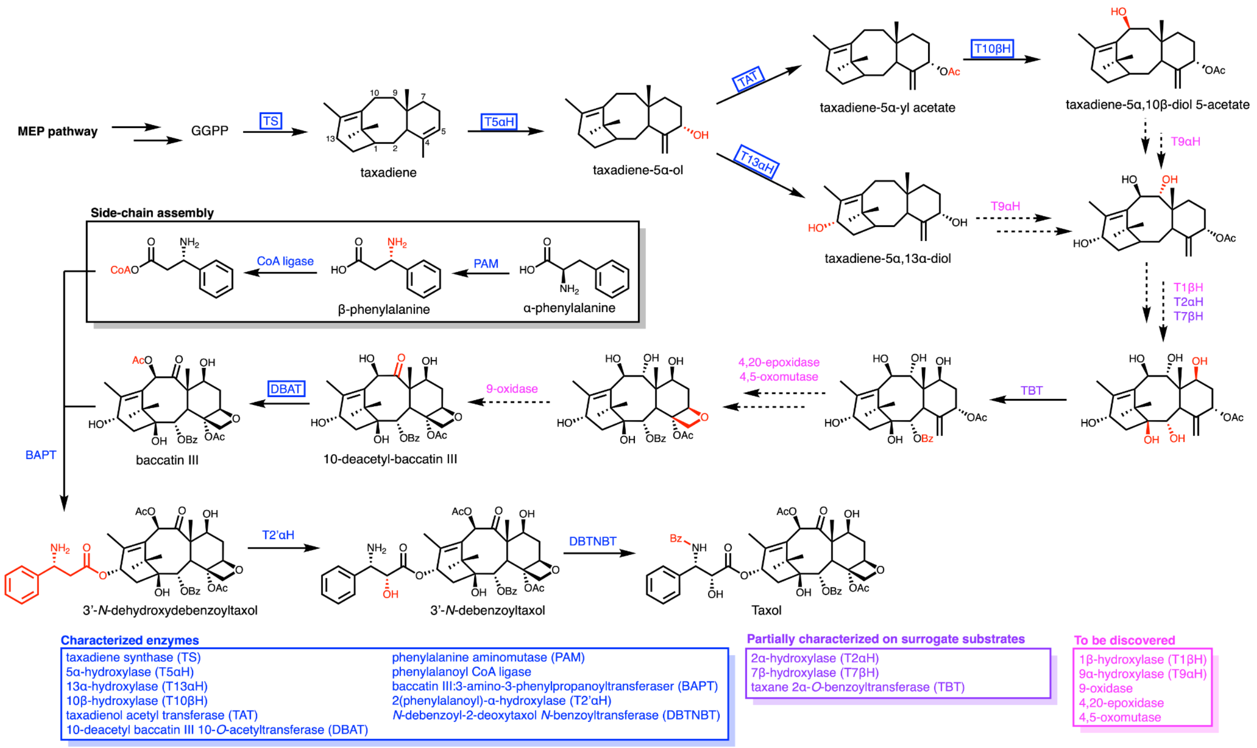

Supplementary Fig. 1. Proposed paclitaxel biosynthetic pathway. Paclitaxel biosynthesis is proposed to involve 19 enzymes, including 11 well-characterized enzymes (blue), 3 partially characterized enzymes (purple), and 5 enzymes yet to be discovered. Enzymes used in this study are circled with boxes. Due to unavailability of early pathway intermediate, T2αH and T7bH were characterized with surrogate substrate taxusin (5α,9α,10β,13α-tetraacetoxytaxadiene)[1] while TBT was characterized with a 2-*O*-debenzoyl-13-*O*-acetyl baccatin III derivative.[2]

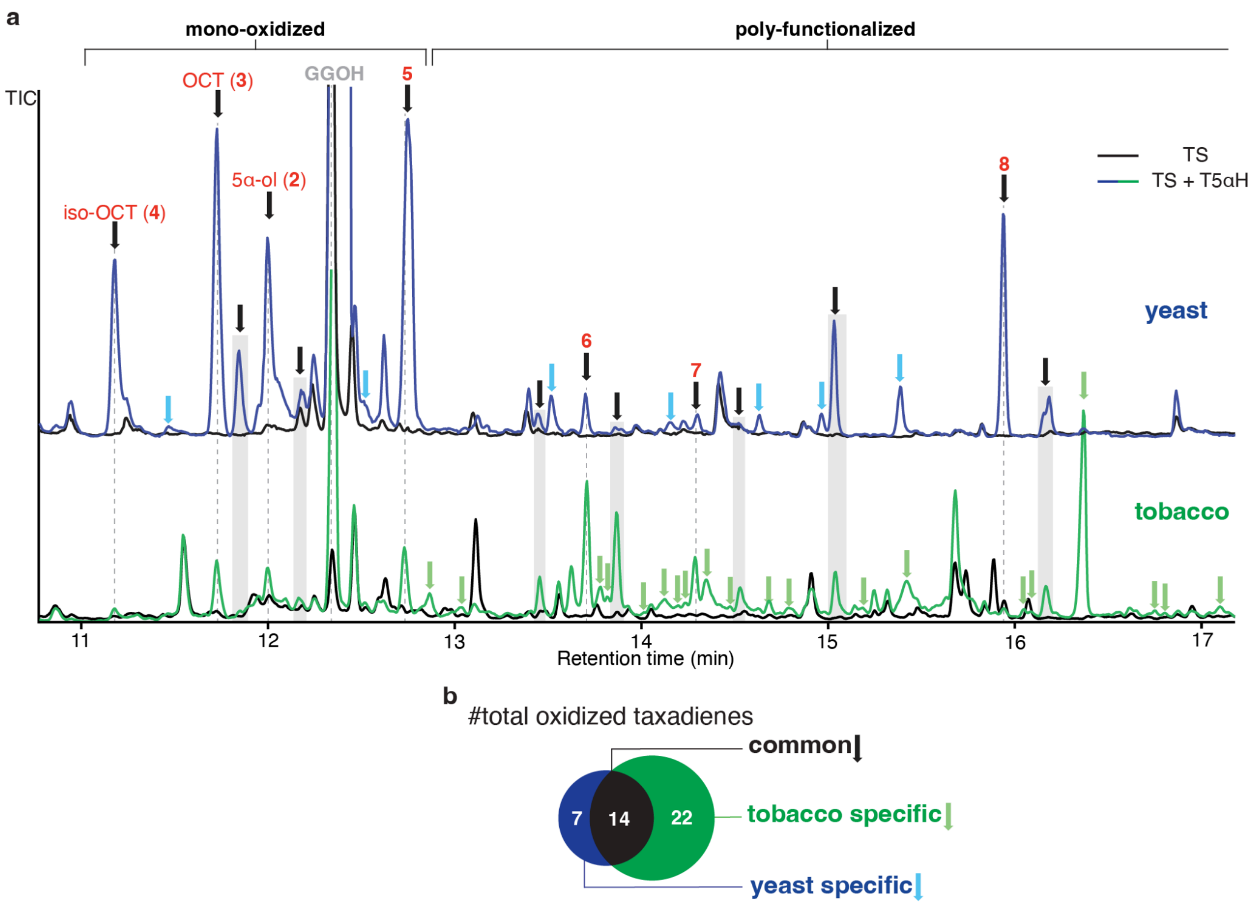

Supplementary Fig. 2. T5αH product profile in yeast and tobacco. a GCMS total ion chromatograms (TICs) of *TS* alone (black trace) and *TS*+*T5αH* (green or blue) expressed in tobacco (*N. benthamiana*) and yeast (*S. cerevisiae*). For the tobacco system, *T5αH* is transiently expressed under 35S promoter in pEAQ-HT vector[3] with truncated 3-hydroxy-3-methylglutaryl coenzyme-A reductase (t*HMGR*), geranylgeranyl diphosphate synthase (*GGPPS*), cytosolic *TS*.[4] For the yeast system (strain JBEI-18128; **Supplementary Table 11**), *TS* and *T5αH* is chromosomally integrated and expressed under Gal80 promoter in an engineered strain with increased diterpenoid production. Green arrows indicate oxidized taxadiene peaks specific to the tobacco system; blue arrows indicate oxidized taxadiene peaks specific to the yeast system; black arrows indicate oxidized taxadiene peaks present in both tobacco and yeast system, including structurally characterized taxadien-5α-ol (**2**), OCT (**3**), iso-OCT (**4**), **5-8**. **b** Venn diagram of the oxidized taxadiene peaks from yeast and tobacco.

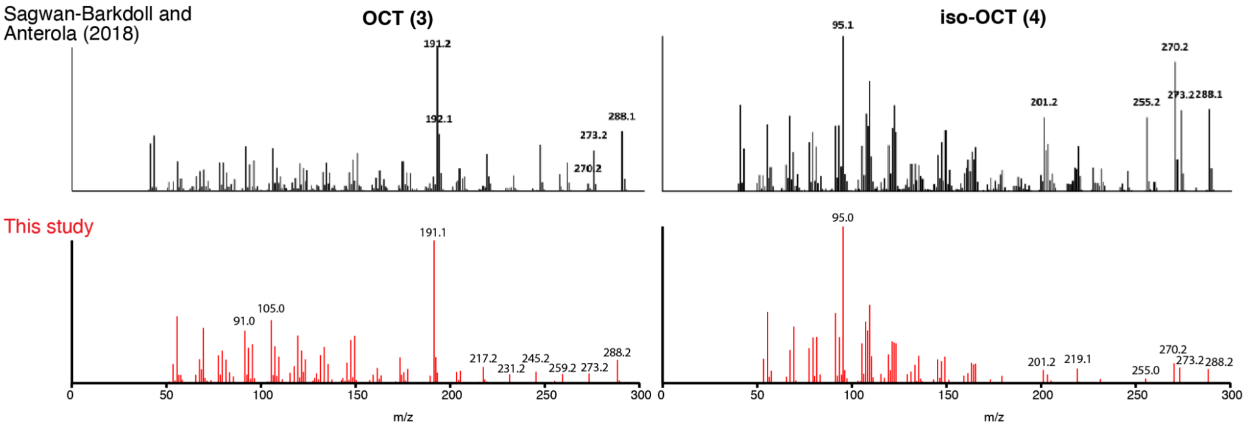

Supplementary Fig. 3. Mass spectra of OCT and iso-OCT in the literature and this study. Mass spectra of the proposed OCT (**3**) and iso-OCT (**4**) peaks in this study compared to those previously reported[5]. The presence of diagnostic ions for OCT (m/z 288, 273, and 191) and for iso-OCT (m/z 288, 270, 255, 201, and 95) confirmed the identification of OCT and iso-OCT in this study.

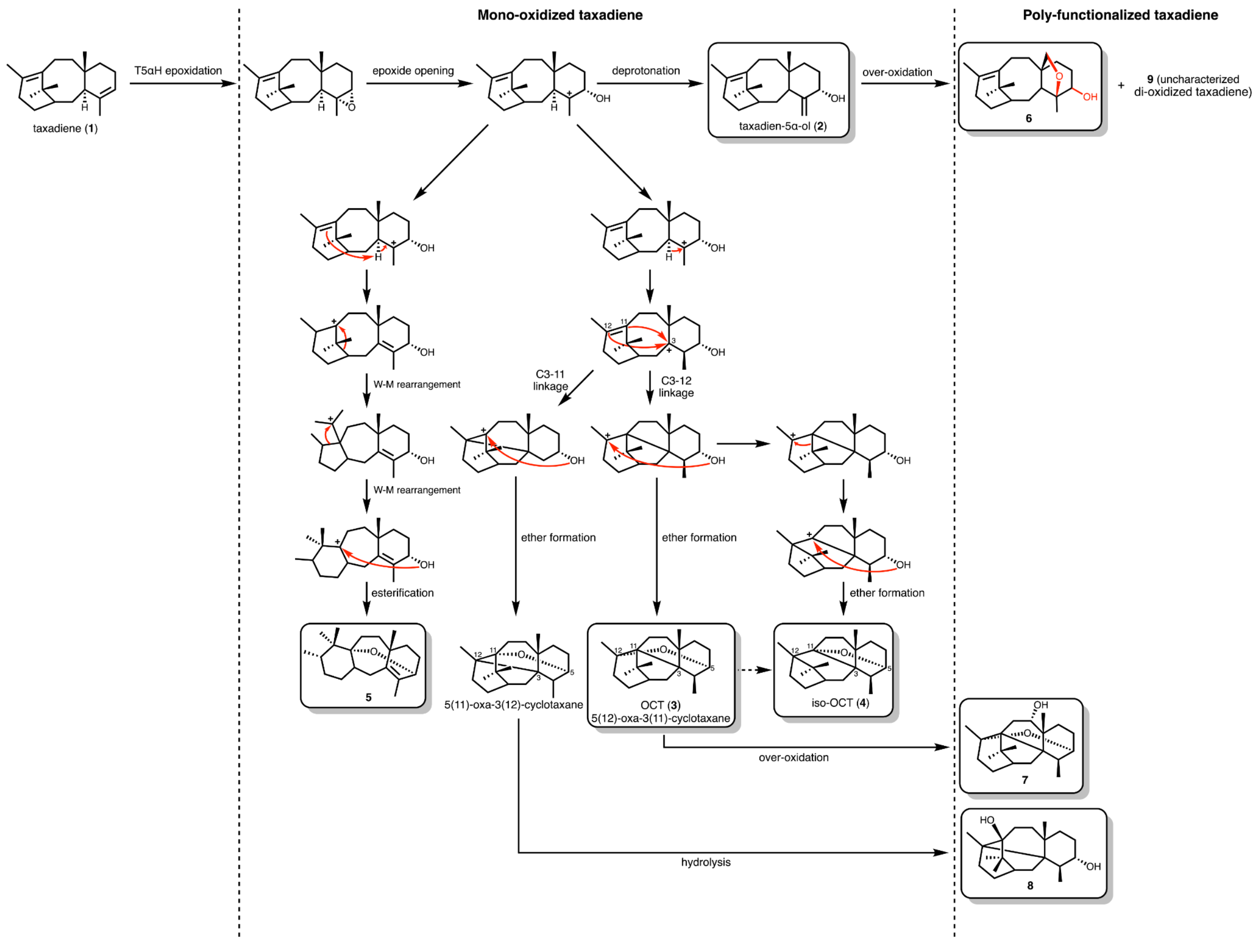

Supplementary Fig. 4. Proposed mechanisms for the formation of oxidized taxadiene 2-9. We have proposed mechanisms for the formation of **2**-**9** based on previously proposed T5αH epoxidation mechanism[6]. Compound **2**-**8** whose structures are confirmed in this study are highlighted in the box. The unstable epoxide ring-opened intermediate can either undergo deprotonation to yield taxadien-5α-ol (**2**), or a series of proton shifts and cation rearrangements [including Wagner-Meerwein (W-M) rearrangement] to reach OCT (**3**), iso-OCT (**4**), **5**, and 5(11)-oxa-3(12)-cyclotaxane. It is proposed that some of these mono-oxidized taxadiene can then be hydrolyzed or further over-oxidized by T5αH to generate further modified products including **6**-**9**. Dashed arrow indicates that spontaneous rearrangement directly from OCT (**3**) to iso-OCT (**4**) is also possible as previously reported[7,8].

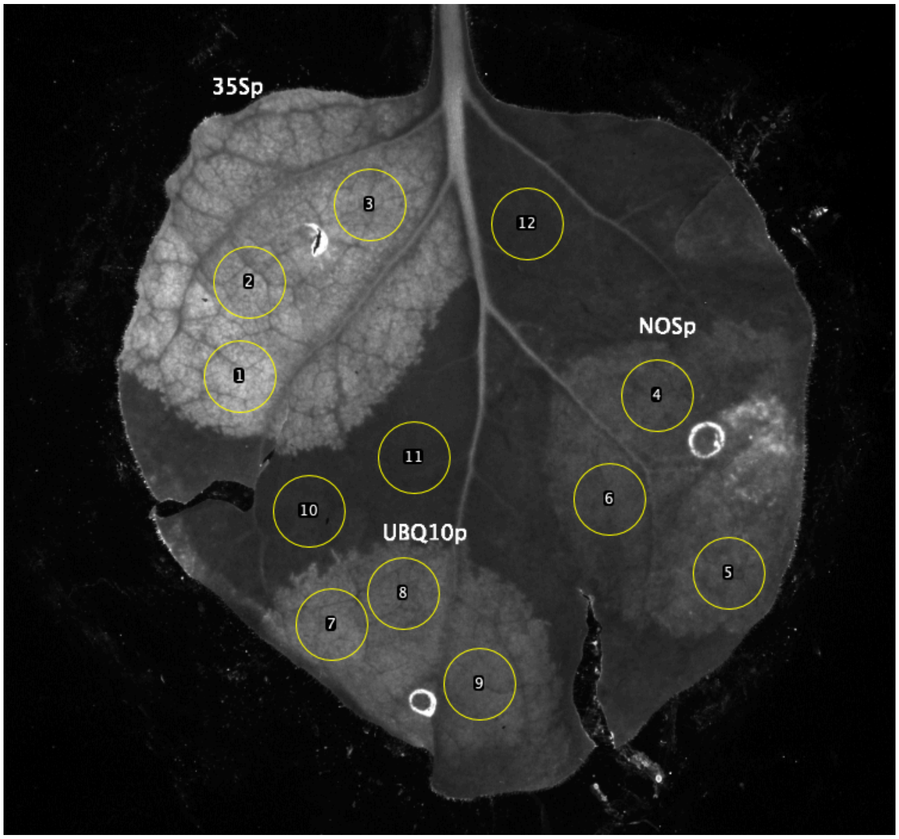

Supplementary Fig. 5. Fluorescence intensity of GFP when expressed under 35S, NOS, or UBQ10 promoter. Fluorescence of GFP in standard pEAQ-HT vector with 35S promoter[3] and in engineered pEAQ-HT vector where the 35S promoter is replaced with either NOS or UBQ10 promoter. Leaf sample (5-day post infiltration) was excited by UV and imaged on the Bio-Rad Gel Doc XR+ system. Image was processed on Fiji ImageJ to measure the average gray value (0-255) of three circled area in 35S (numbered 1-3), NOS (numbered 4-6), UBQ10 (numbered 7-10) and background (numbered 10-12) regions. The measured gray values were normalized to background, and the ratio of average gray values in 35S, UBQ10, NOS areas was 2.7 : 2.0 : 1.7.

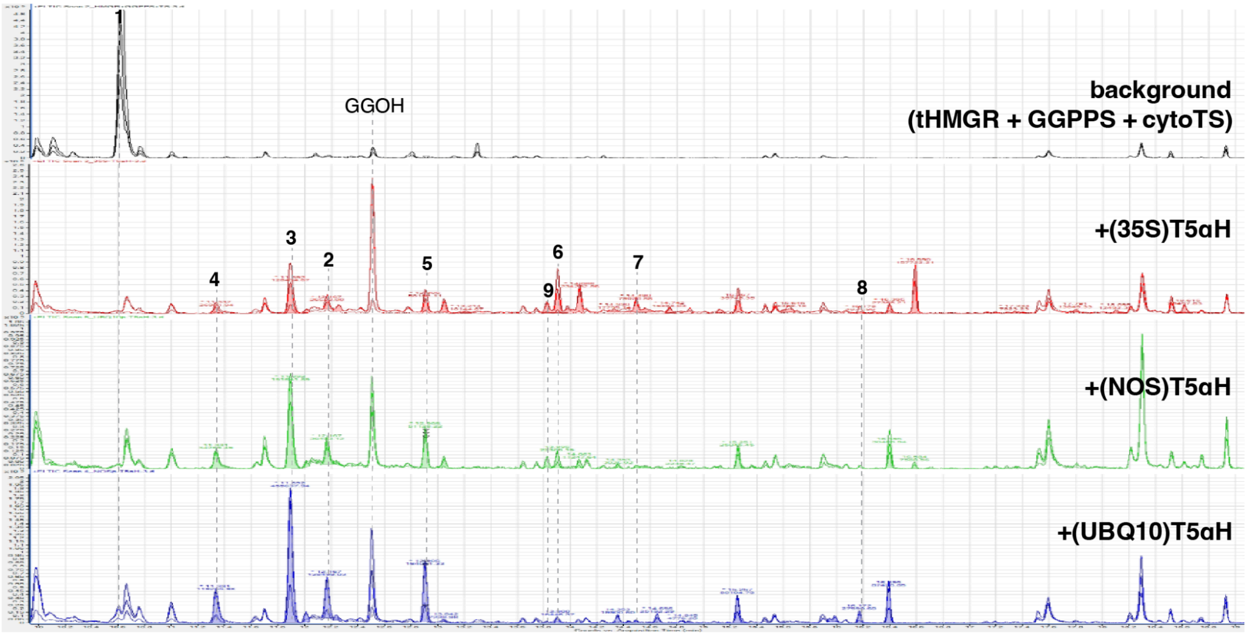

Supplementary Fig. 6. GCMS chromatograms of *T5αH* expressed under different promoters compared to background condition. GCMS total ion chromatograms (TICs) of *Nicotiana benthamiana* leaves transiently expressing background genes (t*HMGR*, *GGPPS* and cyto*TS*)[4] compared to background genes plus *T5αH* expressed under either 35S, UBQ10, or NOS promoter. Triplicate traces for each construct are highlighted in the same color and overlaid. The absolute intensities of oxidized taxadienes show leaf-to-leaf variations, and typically younger leaves show higher products compared to older leaves. Oxidized taxadiene peaks are integrated by Agilent MassHunter Qualitative Analysis software and the result is summarized in **Source Data**.

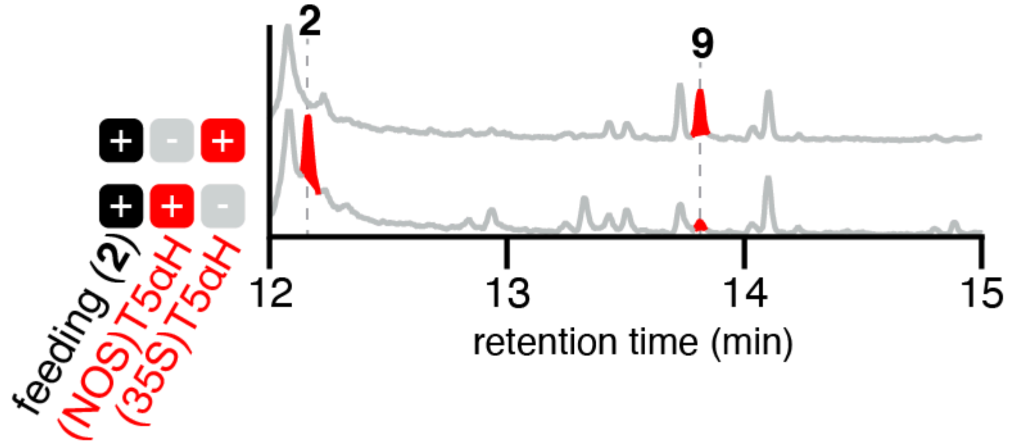

Supplementary Fig. 7. Synthetic 2 fed to *T5αH* expressed under the 35S or NOS promoter. GCMS total ion chromatograms (TICs) of *Nicotiana benthamiana* leaves transiently expressing *T5αH* under NOS or 35S promoter and fed with synthetic taxadien-5α-ol (**2**) at 3-day post infiltration. Representative traces of three biological replicates are shown.

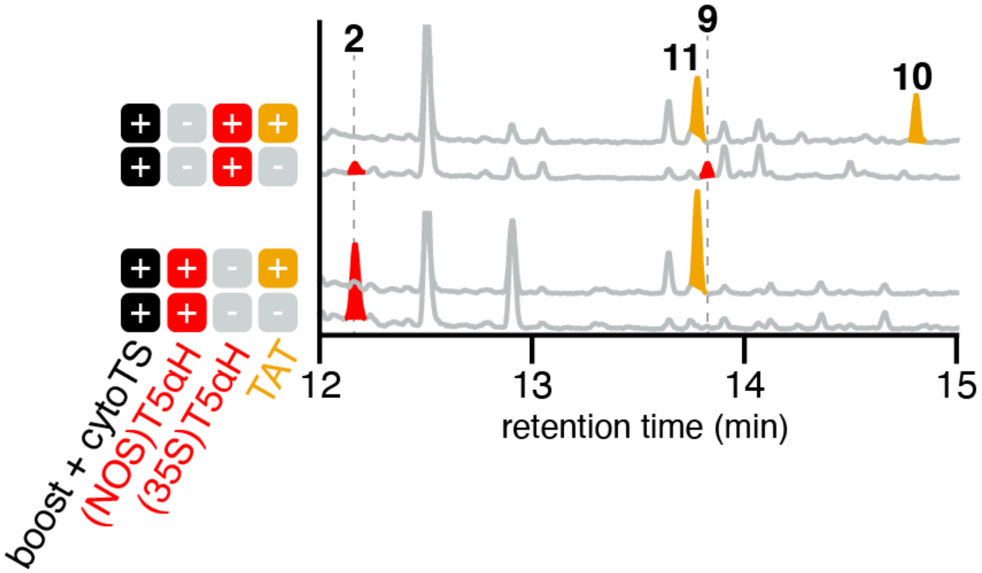

Supplementary Fig. 8. Comparing the effect of 35S and NOS promoter when additional downstream enzyme TAT is introduced. GCMS total ion chromatograms (TICs) of *Nicotiana benthamiana* leaves transiently expressing cytosolic diterpenoid boost (t*HMGR* + *GGPPS*)[4], cyto*TS*, *T5αH* under either 35S or NOS promoter, and taxadien-5α-ol acetyltransferase (*TAT*). T5αH products **2** and **9** are highlighted in red and TAT products **10** and **11** are highlighted in orange. Representative traces of three biological replicates are shown.

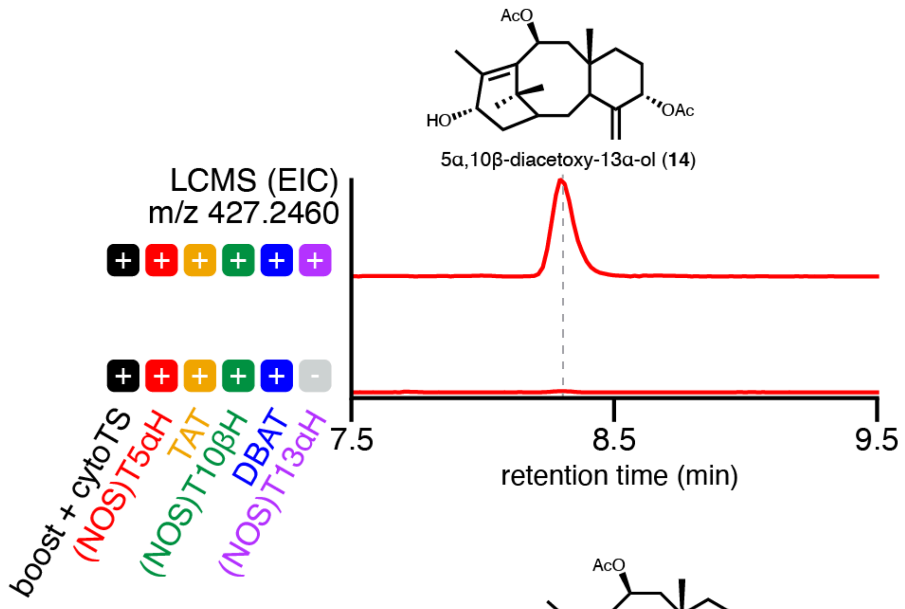

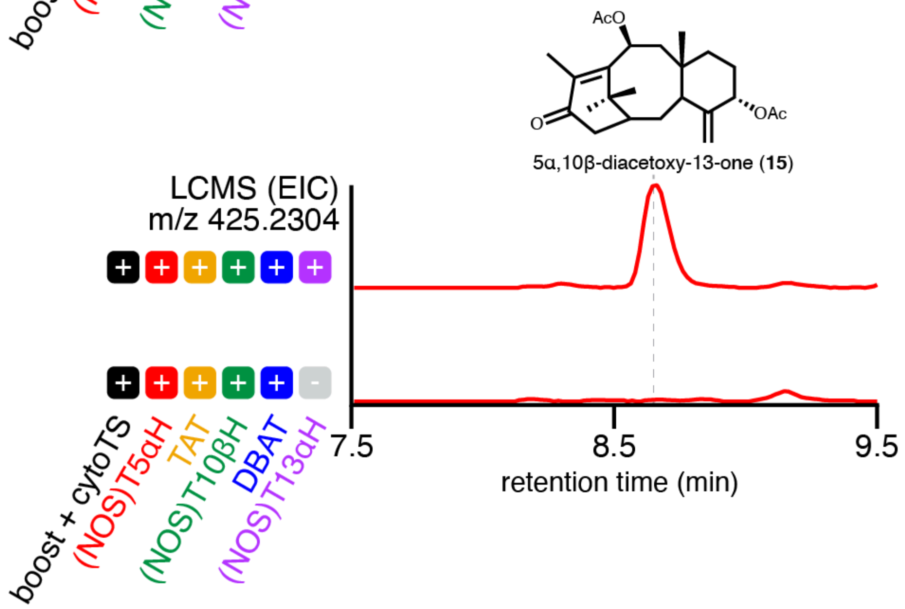

Supplementary Fig. 9. LCMS EIC of compound 14 and 15. LCMS extracted ion chromatogram (EIC) of *Nicotiana benthamiana* leaves transiently expressing cytosolic diterpenoid boost (t*HMGR* + *GGPPS*)[4], cyto*TS*, T5αH under the NOS promoter, taxadien-5α-ol acetyltransferase (*TAT*), *T10βH* under the NOS promoter, and 10-deacetylbaccatin III:10-O-acetyltransferase (*DBAT*), with either *T13αH* under the NOS promoter or not. EIC of compound **14** (**[**M+Na]$^{+}$ = 427.2460) and **15** ([M+Na]$^{+}$ = 425.2304) are shown. “Taxadien” is omitted in the names for simplicity. Representative traces of three biological replicates are shown.

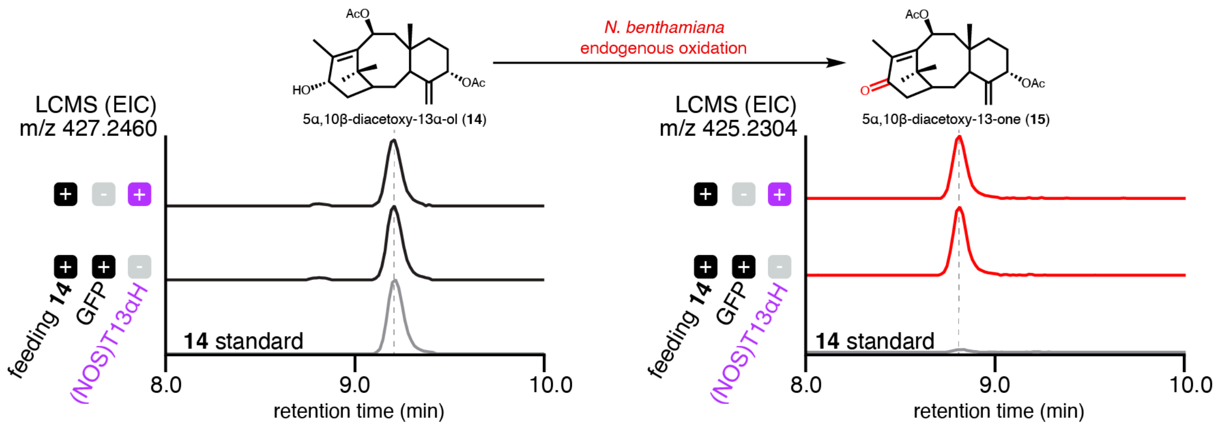

Supplementary Fig. 10. Oxidation of 5α,10β-diacetoxy-13α-ol (14) by *N. benthamiana* endogenous enzymes. LCMS extracted ion chromatogram (EIC) of *Nicotiana benthamiana* leaves transiently expressing *GFP* (middle trace) or *T13αH* under the NOS promoter (top trace) and fed with 400 uM **14**. In both conditions, formation of **15** was observed, suggesting that T13αH is not involved in the formation of **15** but rather *N. benthamiana* endogenous enzymes. EIC of **14** standard used for the feeding was shown (bottom trace) to show that **15** is only present at a low level as a co-purified contaminant. "Taxadien" is omitted in the names for simplicity.

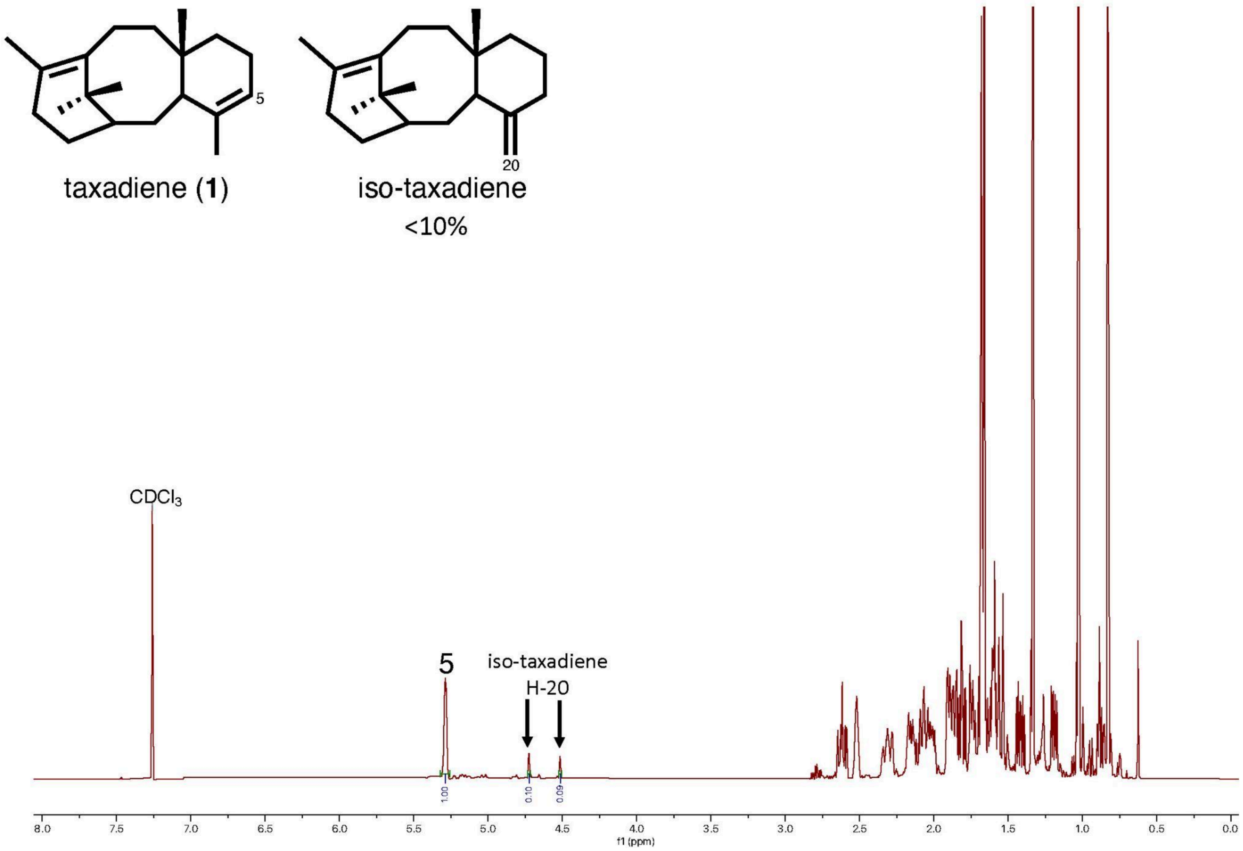

Supplementary Fig. 11. ^{1}H-NMR spectrum of taxadiene (1) in $CDCl_3$ (600 Hz, n = 16).

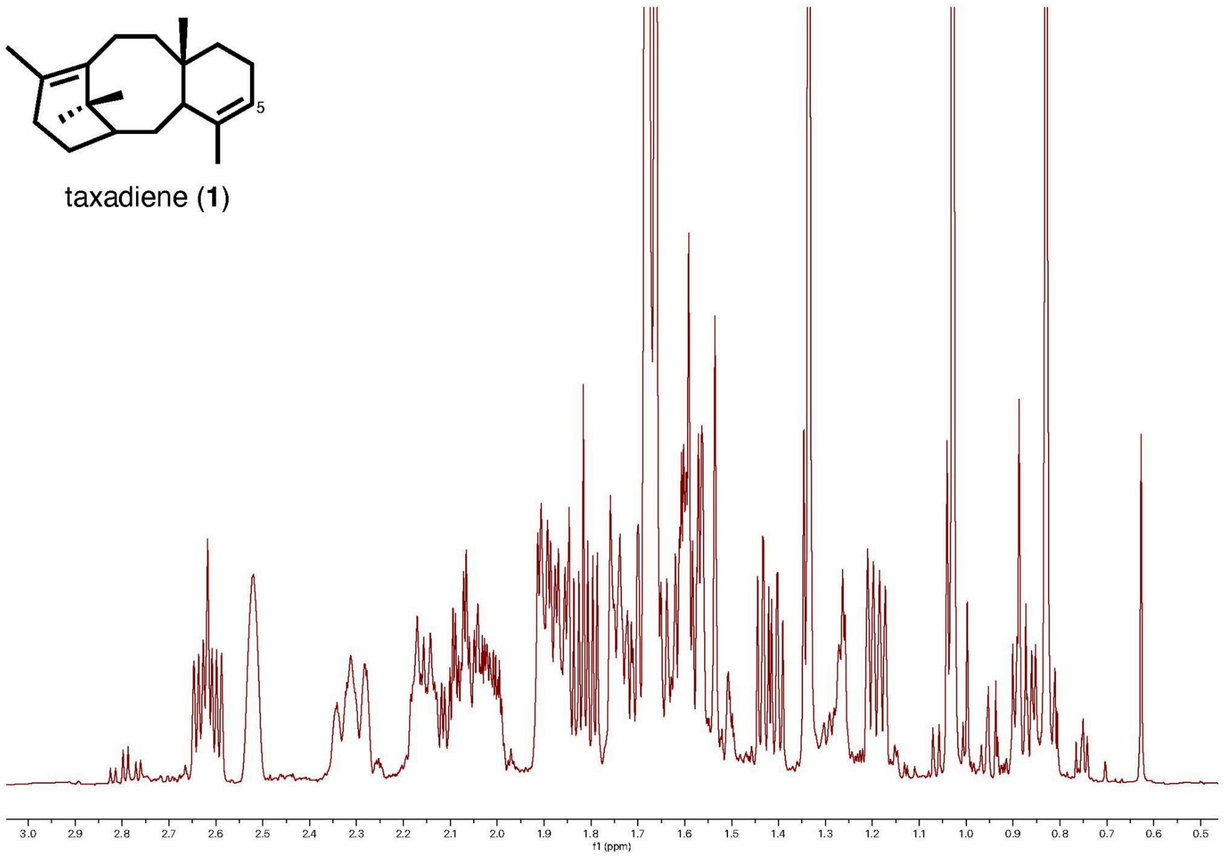

Supplementary Fig 12. ^{1}H NMR spectrum of taxadiene (1) in $CDCl_3$ (600 Hz, n = 16) in the region of 0.5~3.0 ppm.

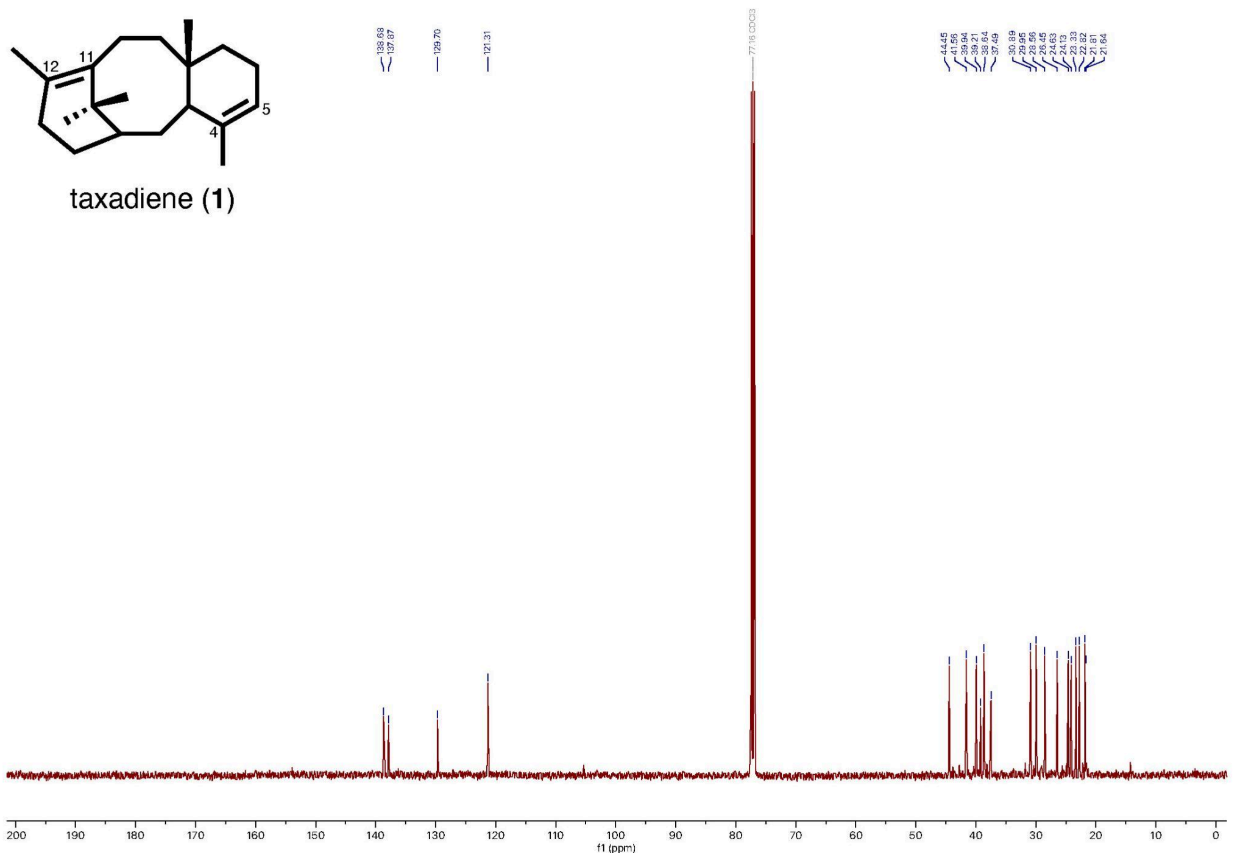

Supplementary Fig. 13. ^{13}C NMR spectrum of taxadiene (1) in $CDCl_3$ (500 Hz, n = 512). Chemical shifts (ppm): 138.68, 137.87, 129.70, 121.31, 44.45, 41.56, 39.94, 39.21, 38.64, 37.49, 30.89, 29.95, 28.56, 26.45, 24.63, 24.13, 23.33, 22.82, 21.81, 21.64.

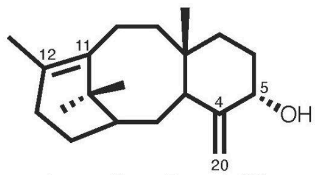

taxadien-5α-ol (**2**)

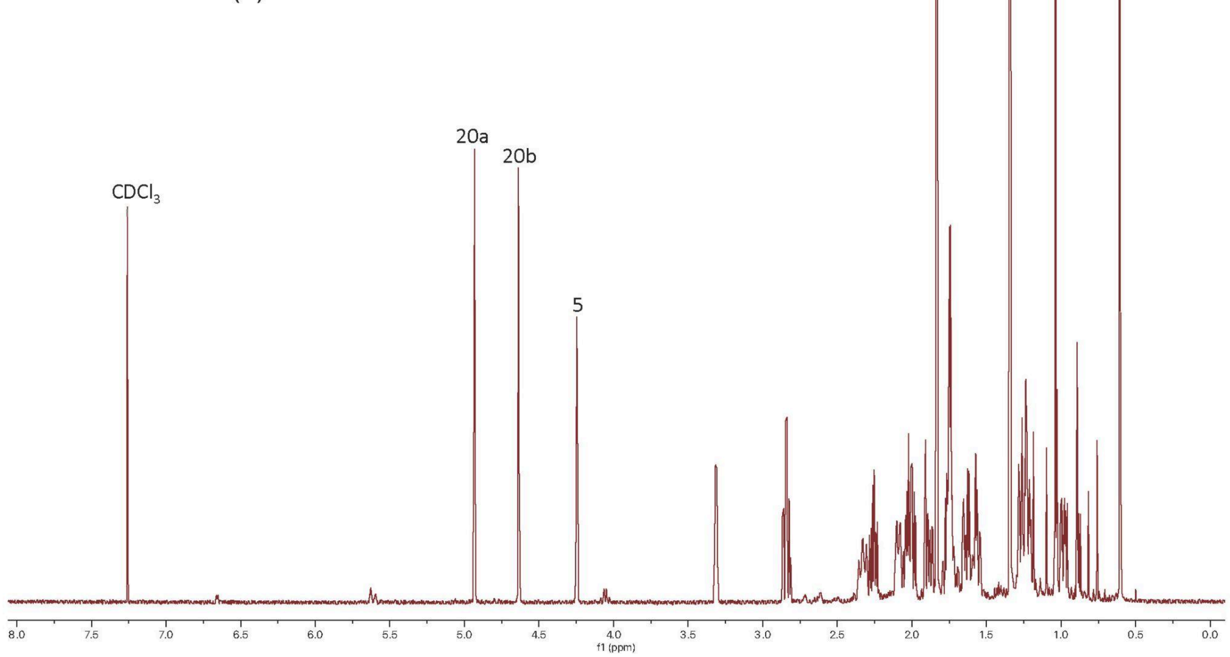

Supplementary Fig. 14. ^{1}H-NMR spectrum of taxadien-5α-ol (2) in $CDCl_3$ (600 Hz, n = 32).

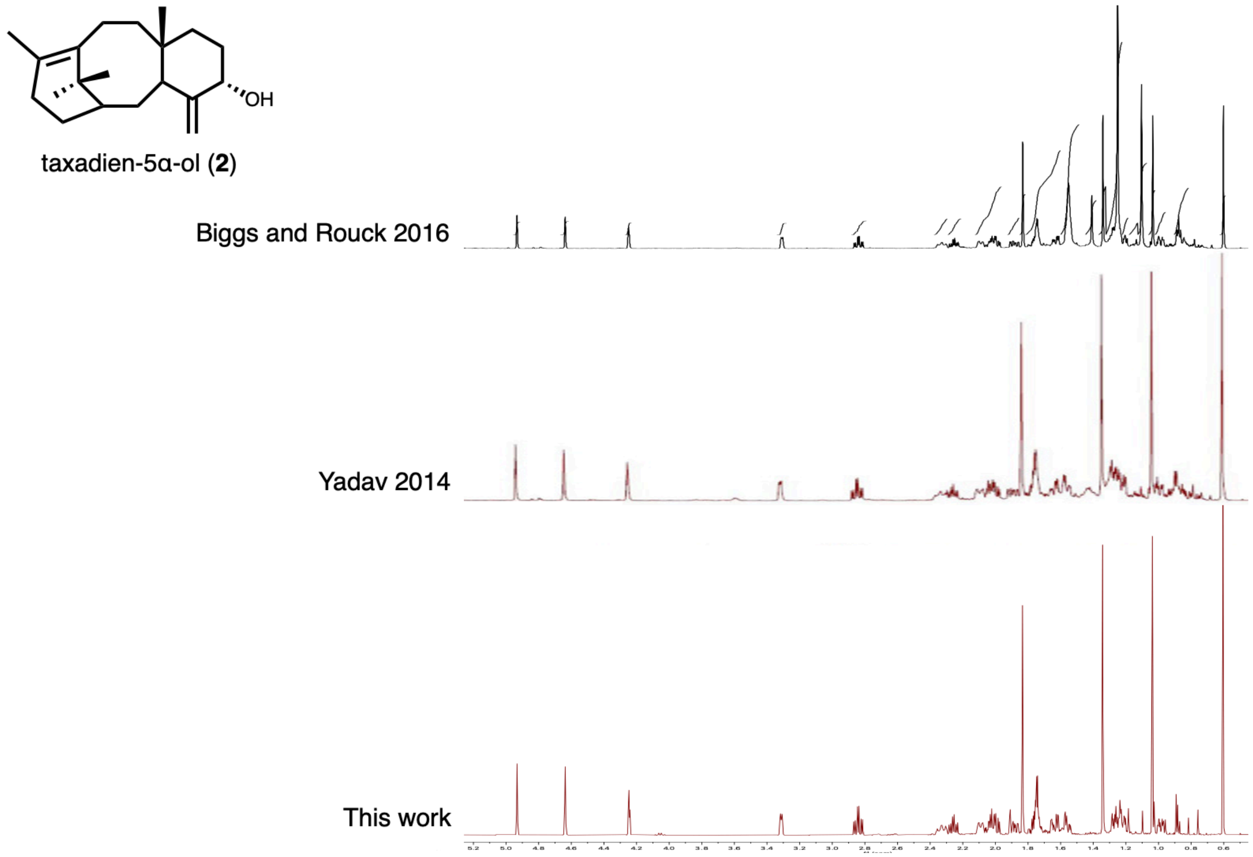

Supplementary Fig. 15. ¹H-NMR spectra of taxadien-5α-ol (2) from previous works and this work in the region of 0.6~5.2 ppm.

1H-NMR spectrum of **2** purified from engineered *E. coli* expressing TS and T5αH in two previous works[7,9] (top 2 traces) are used to confirm the identity of the synthetic **2** in this work.

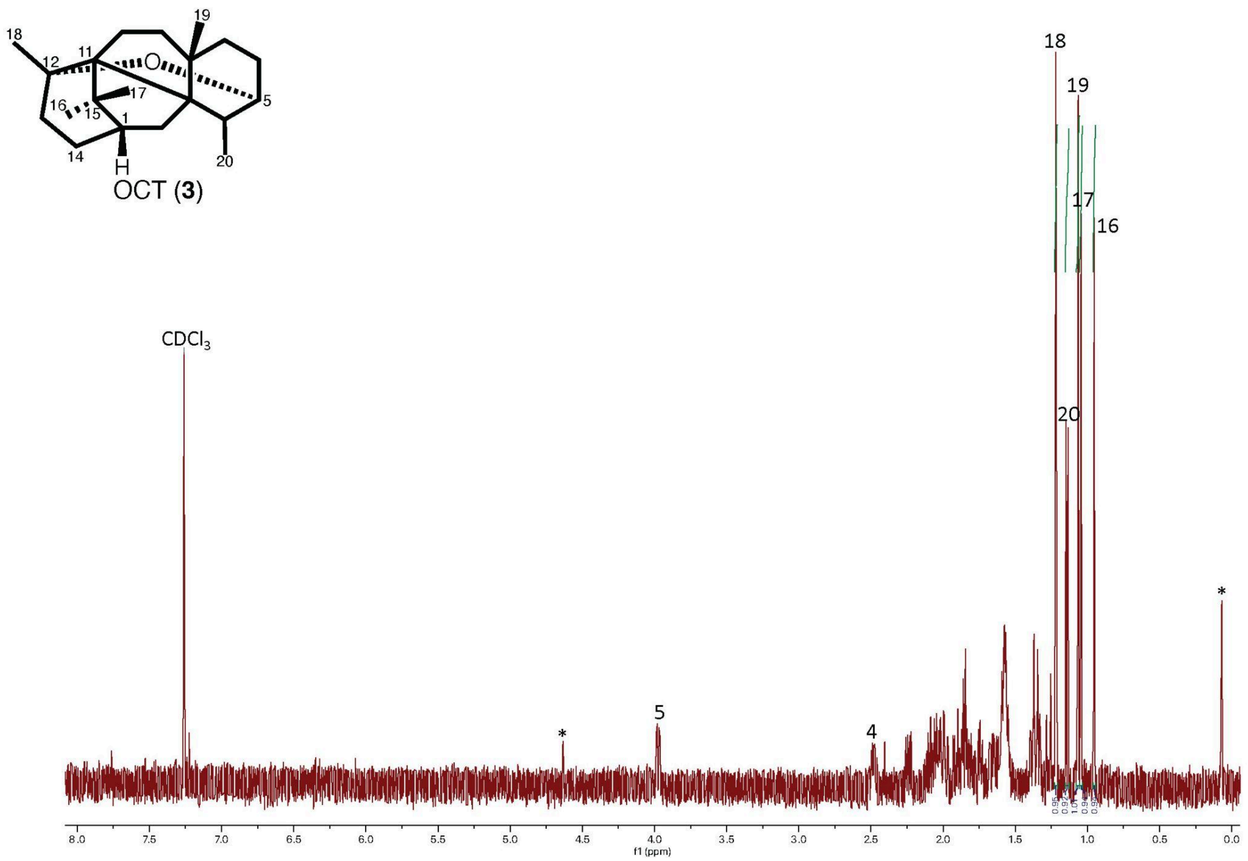

Supplementary Fig. 16. ^{1}H-NMR spectrum of OCT (3) in $CDCl_3$ (500 Hz, n = 64). Asterisk indicates impurities.

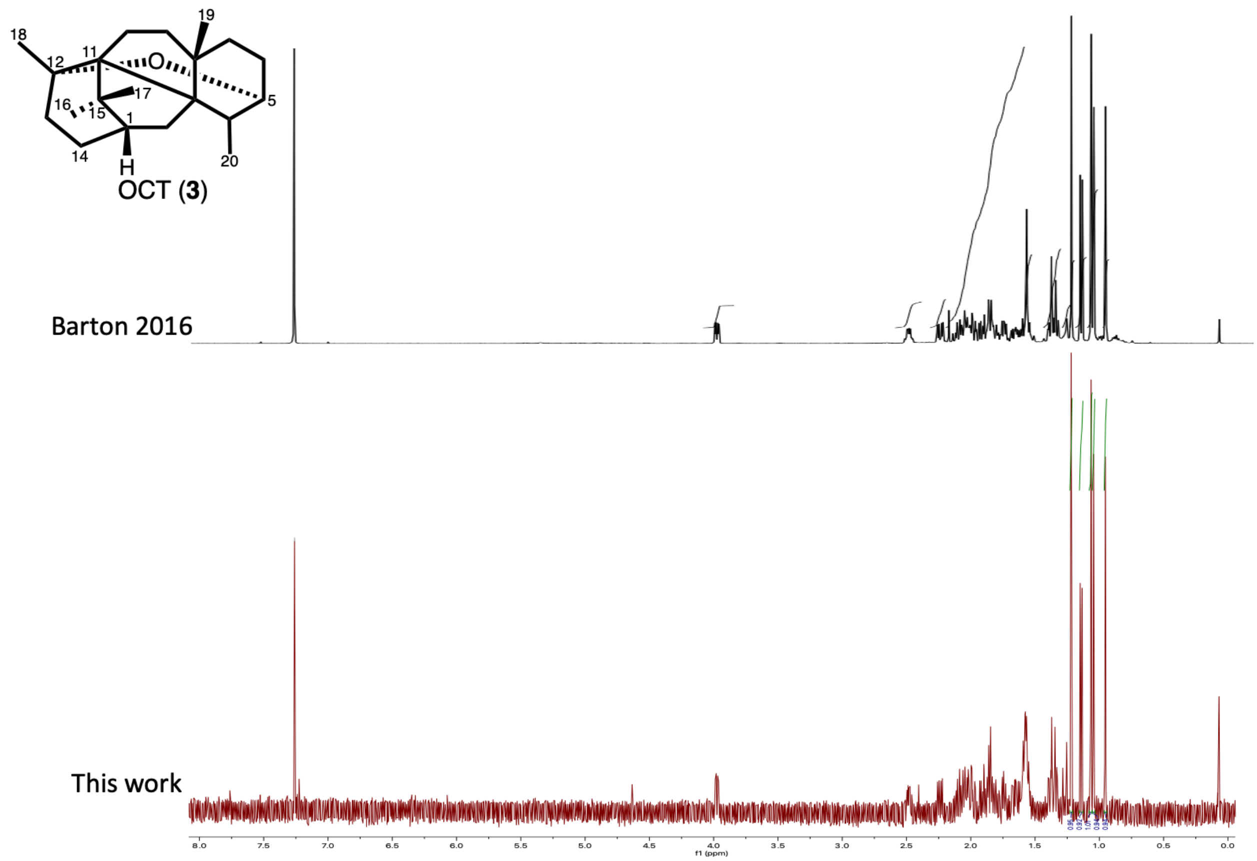

Supplementary Fig. 17. ^{1}H-NMR spectra of OCT (3) from previous work and this work. ^{1}H-NMR spectra of synthetic OCT[6] (top trace) and OCT (**3**) isolated from tobacco in this work (bottom trace).

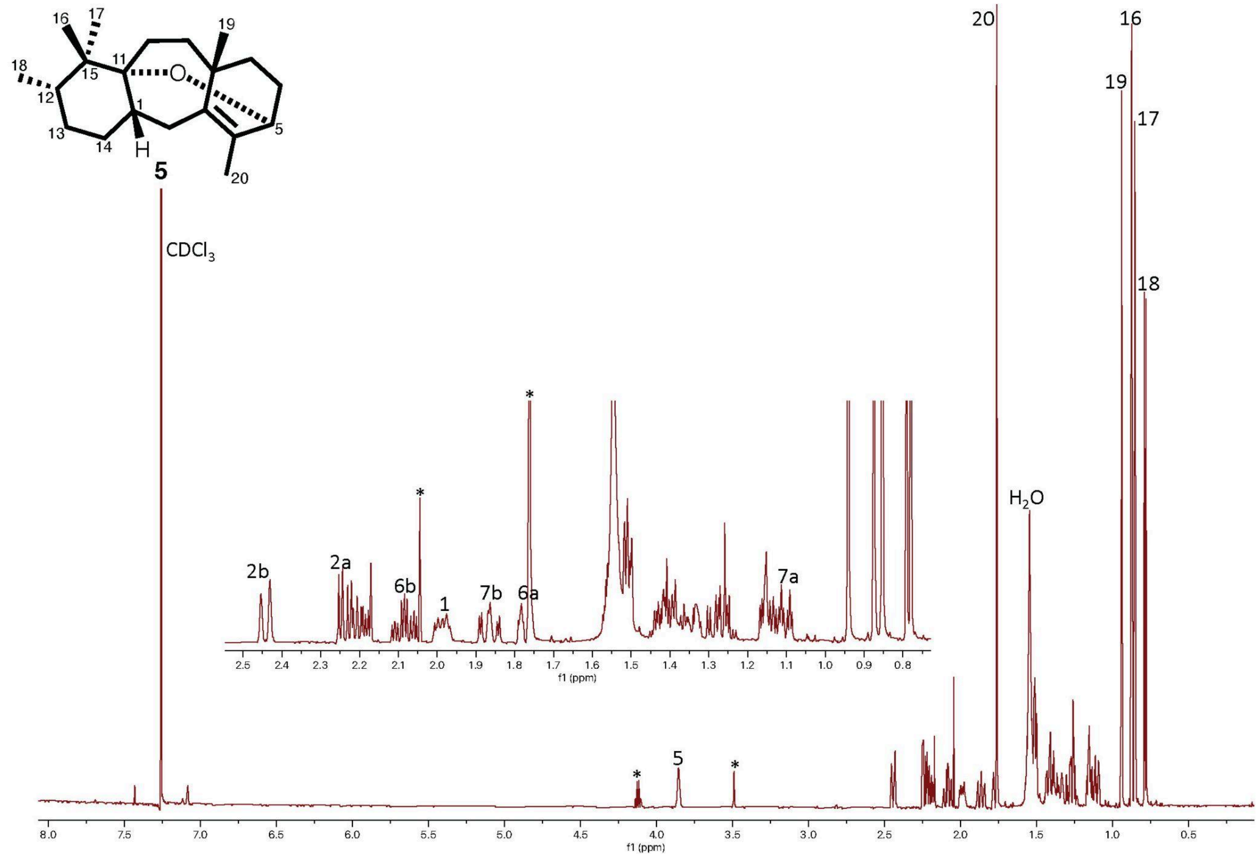

Supplementary Fig. 18. ^{1}H-NMR spectrum of compound 5 in $CDCl_3$ (600 Hz, n = 32). Asterisk indicates impurities.

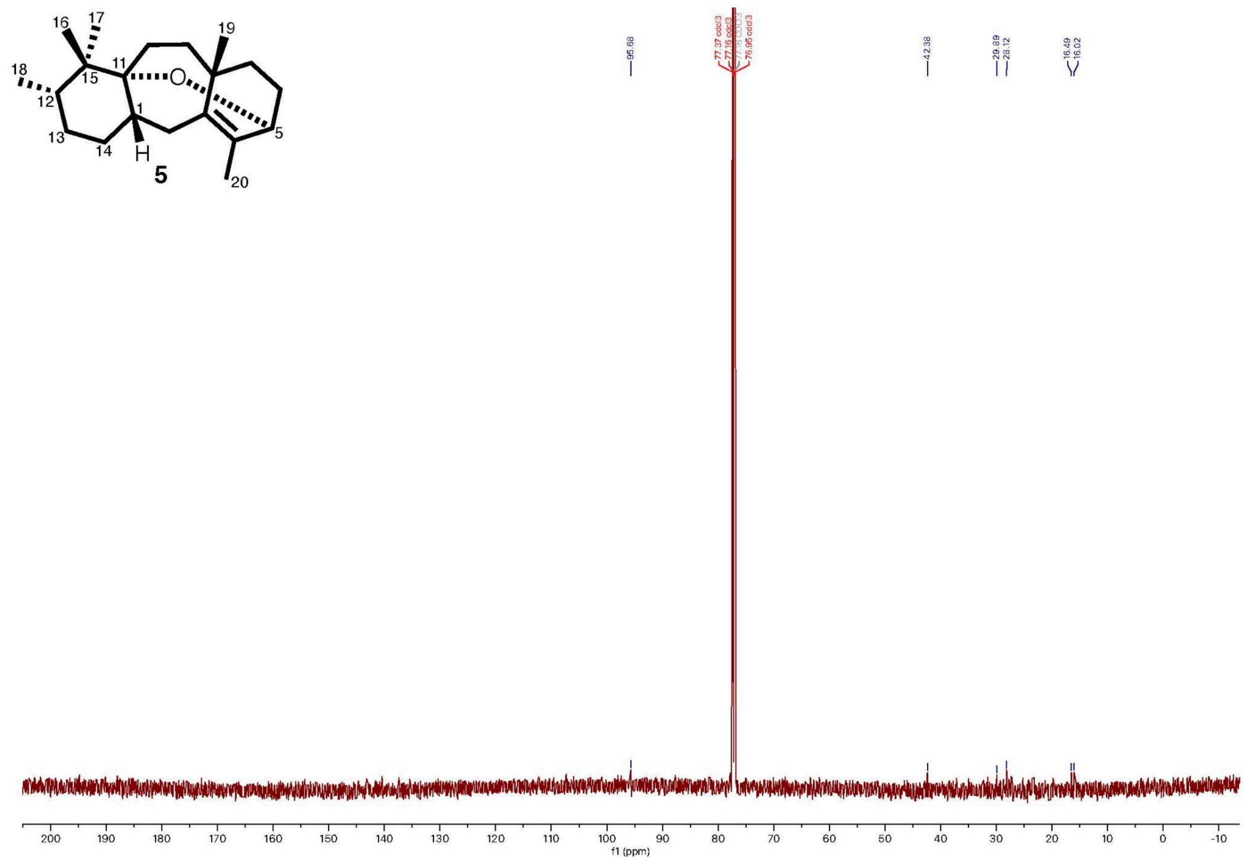

Supplementary Fig. 19. ^{13}C-NMR spectrum of compound 5 in $CDCl_3$ (600 Hz, n = 2048).

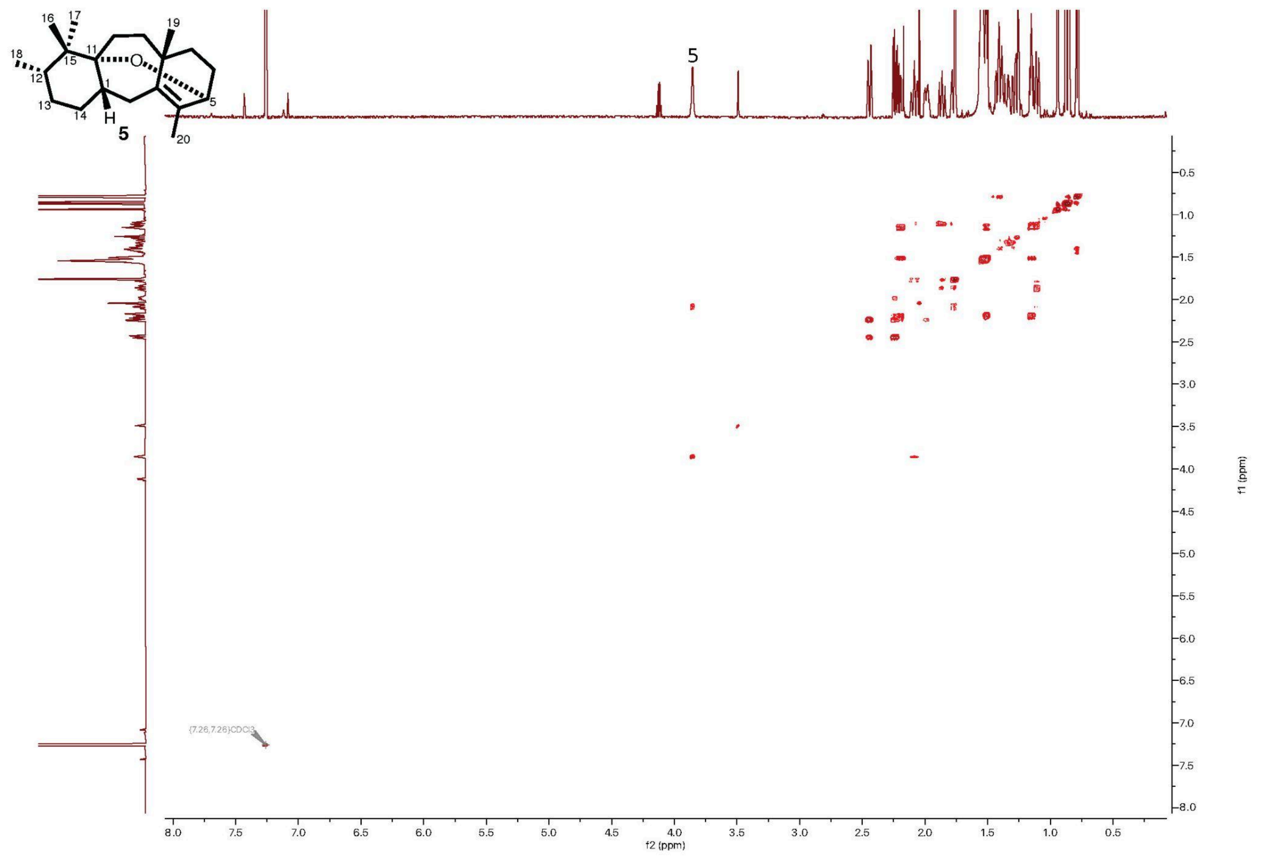

Supplementary Fig. 20. COSY spectrum of compound 5 in $CDCl_3$ (600 Hz, n = 4).

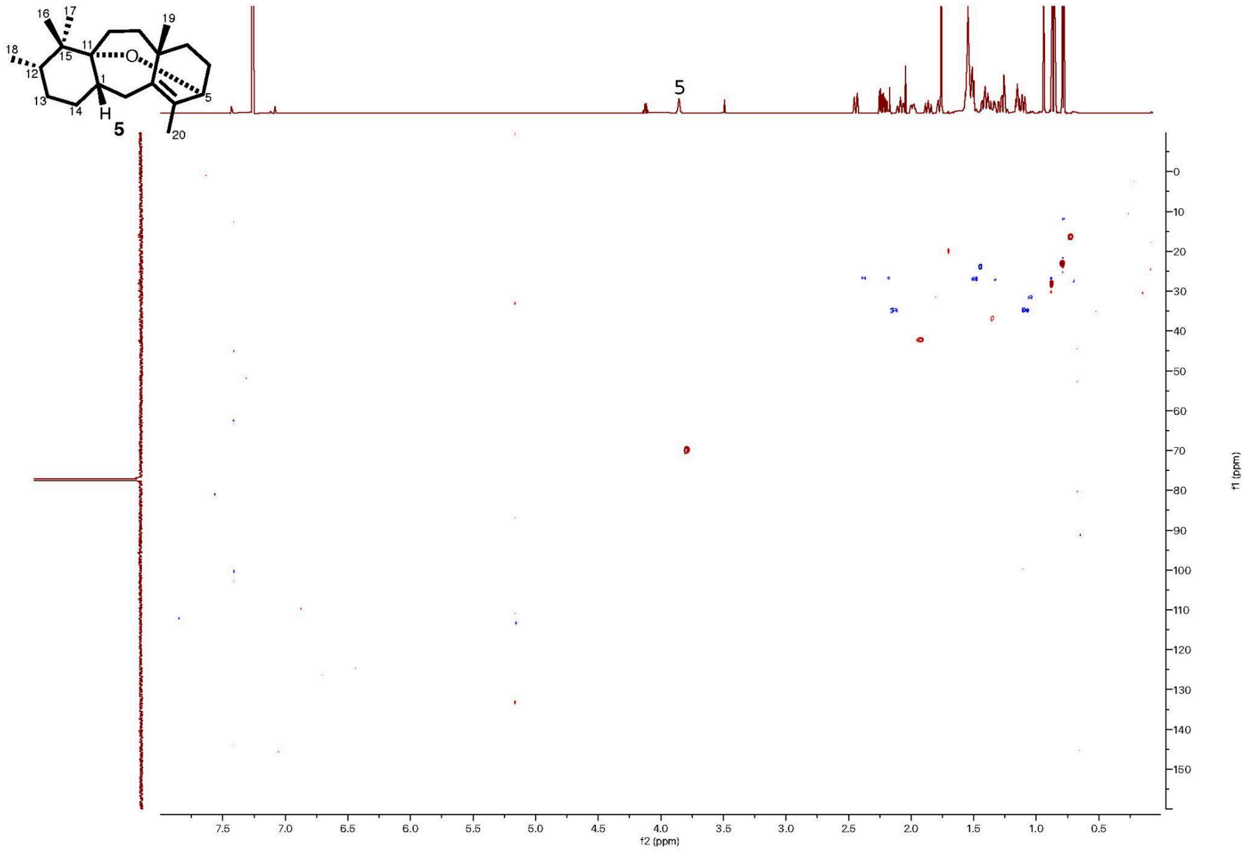

Supplementary Fig. 21. HSQC spectrum of compound 5 in $CDCl_3$ (600 Hz, n = 8).

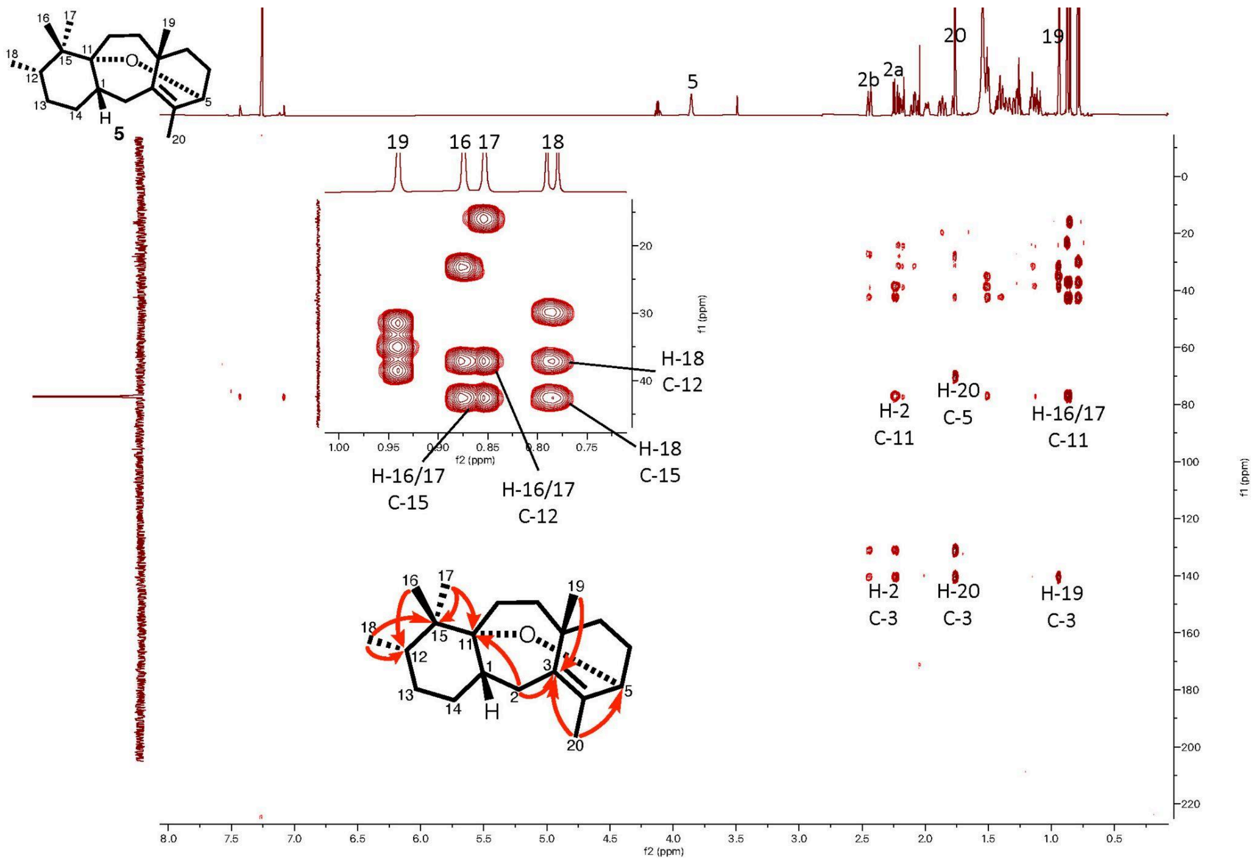

Supplementary Fig. 22. HMBC spectrum of compound 5 in $CDCl_3$ (600 Hz, n = 32).

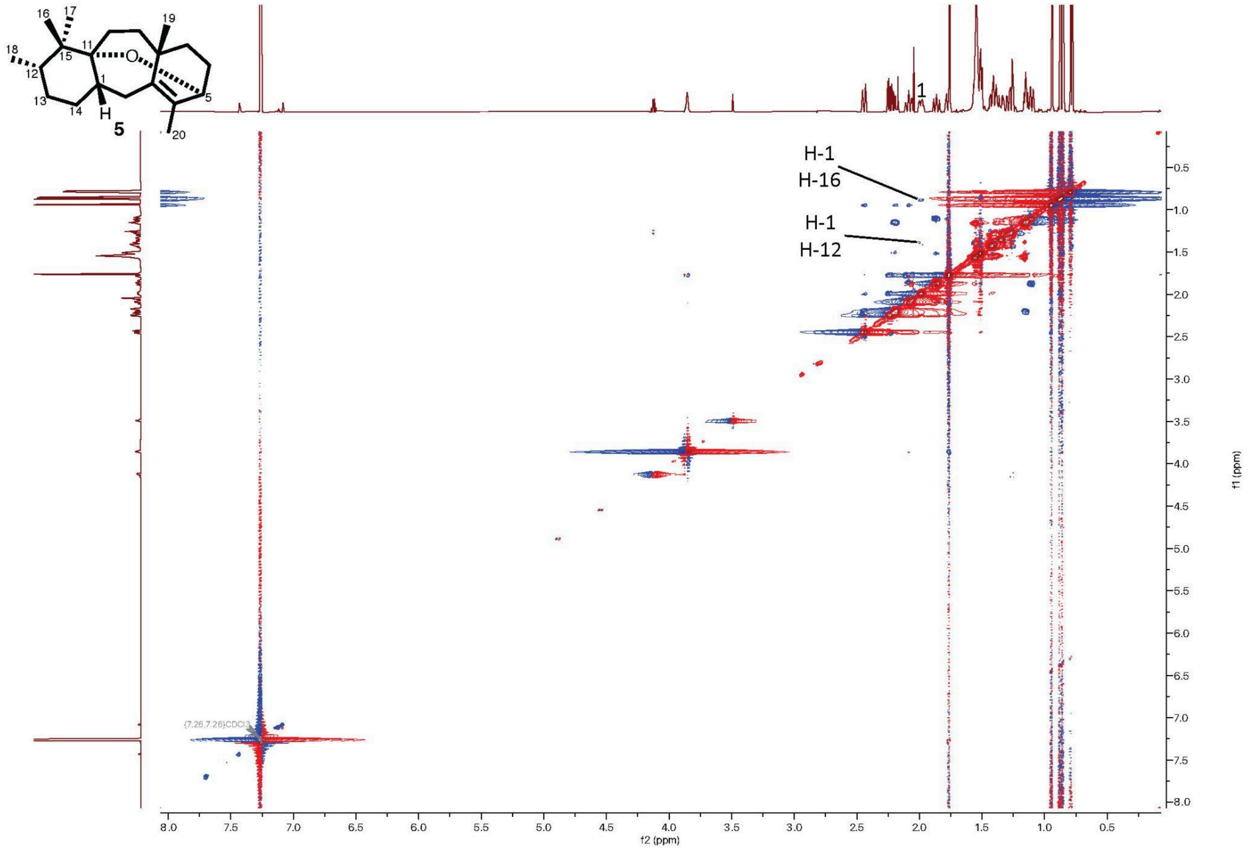

Supplementary Fig. 23. ROESY spectrum of compound 5 in $CDCl_3$ (600 Hz, n = 8).

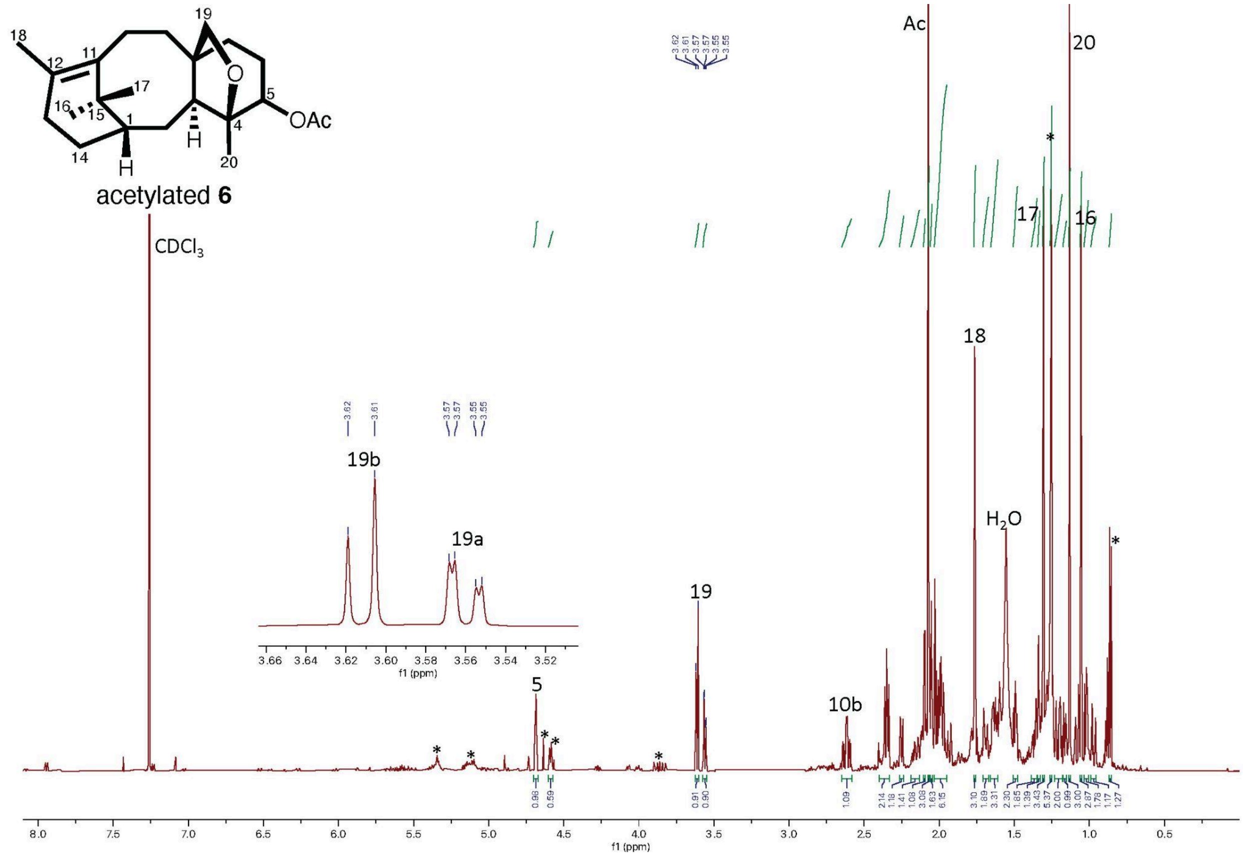

Supplementary Fig. 24. ^{1}H-NMR spectrum of acetylated 6 in $CDCl_3$ (600 Hz, n = 64). Asterisk indicates impurities. The W-coupling of proton 19a is shown.

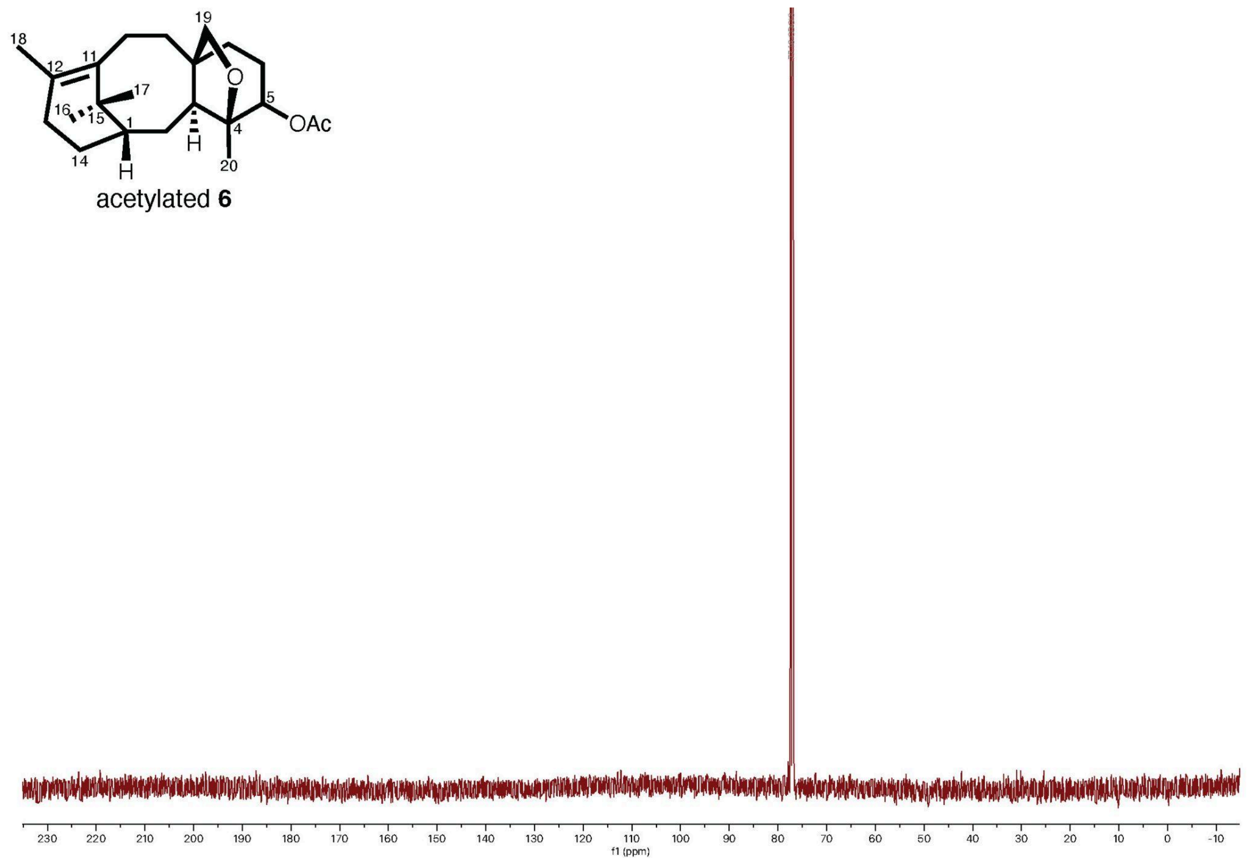

Supplementary Fig. 25. ^{13}C-NMR spectrum of acetylated 6 in $CDCl_3$ (600 Hz, n = 1024).

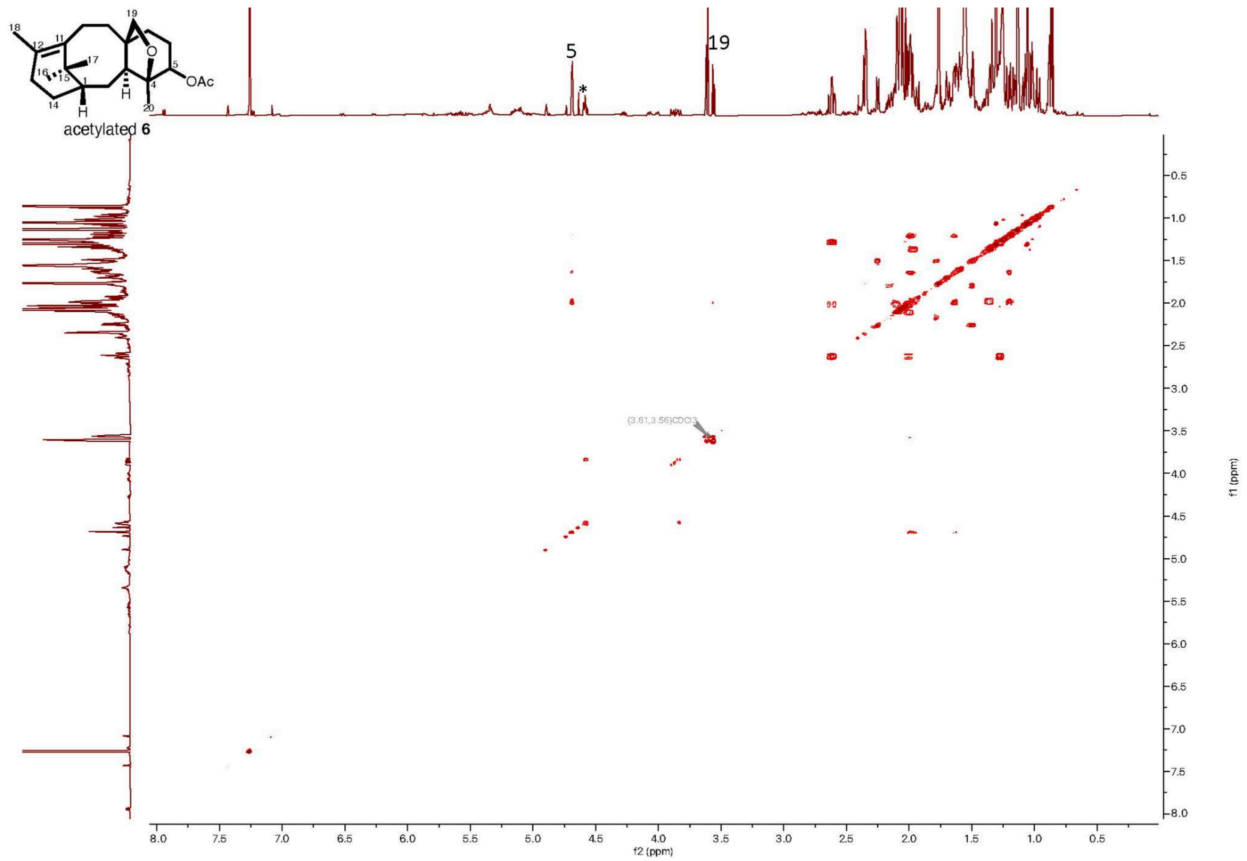

Supplementary Fig. 26. COSY spectrum of acetylated 6 in $CDCl_3$ (600 Hz, n = 4).

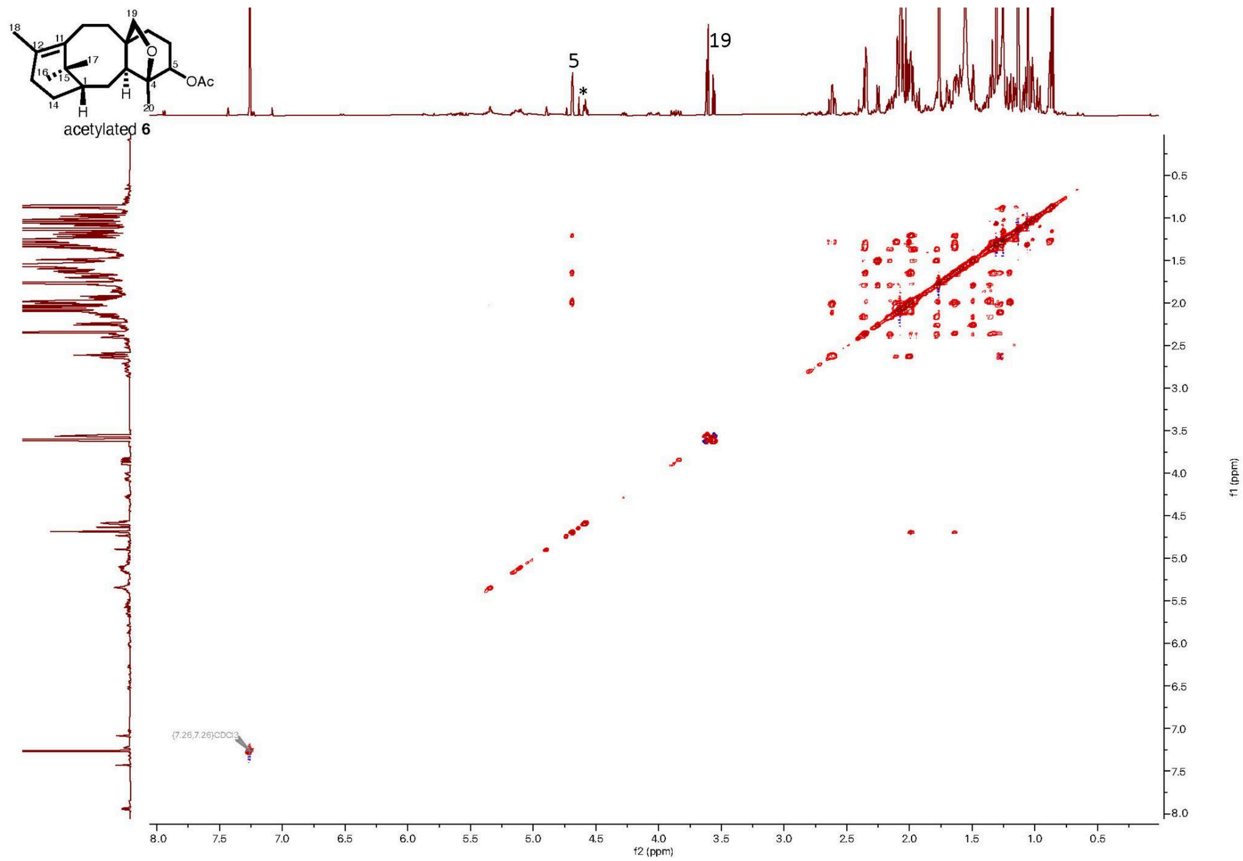

Supplementary Fig. 27. TOCSY spectrum of acetylated 6 in $CDCl_3$ (600 Hz, n = 2).

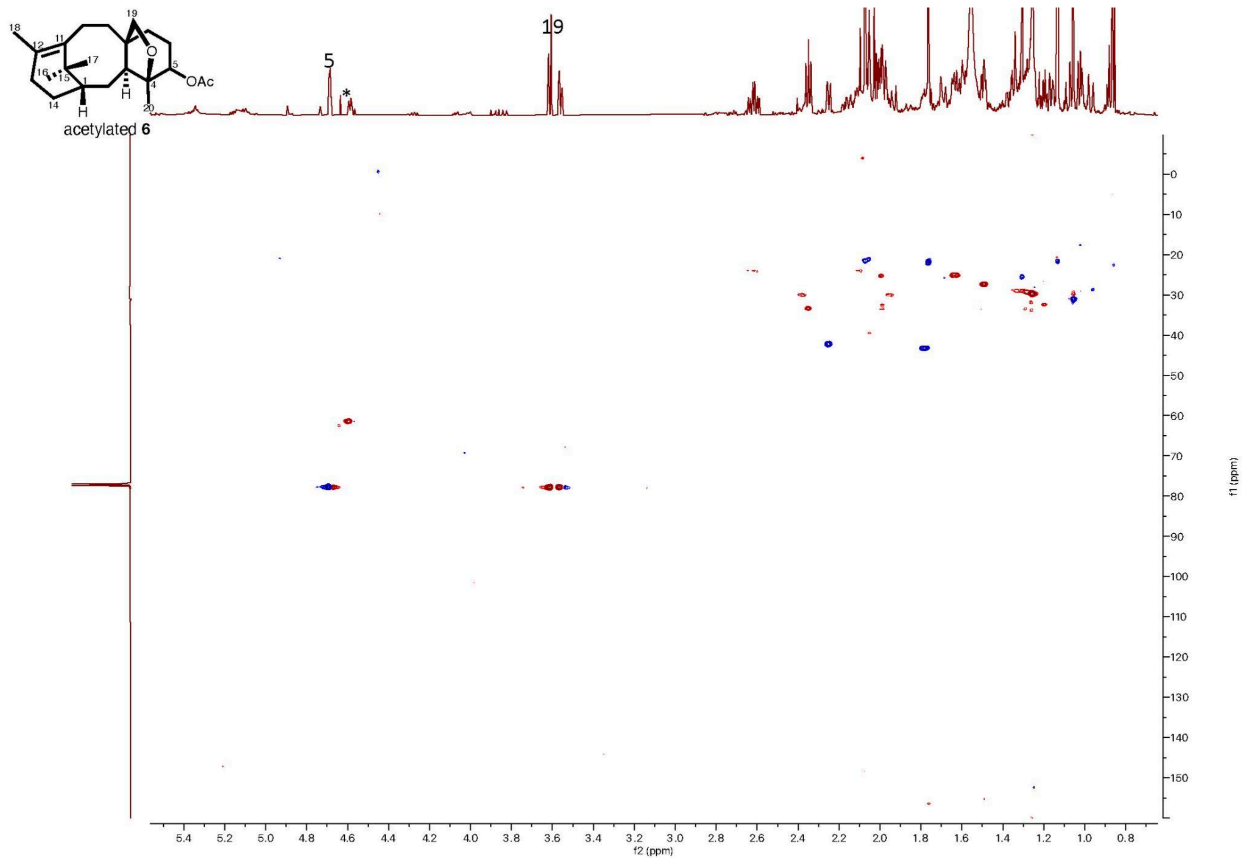

Supplementary Fig. 28. HSQC spectrum of acetylated 6 in $CDCl_3$ (600 Hz, n = 8).

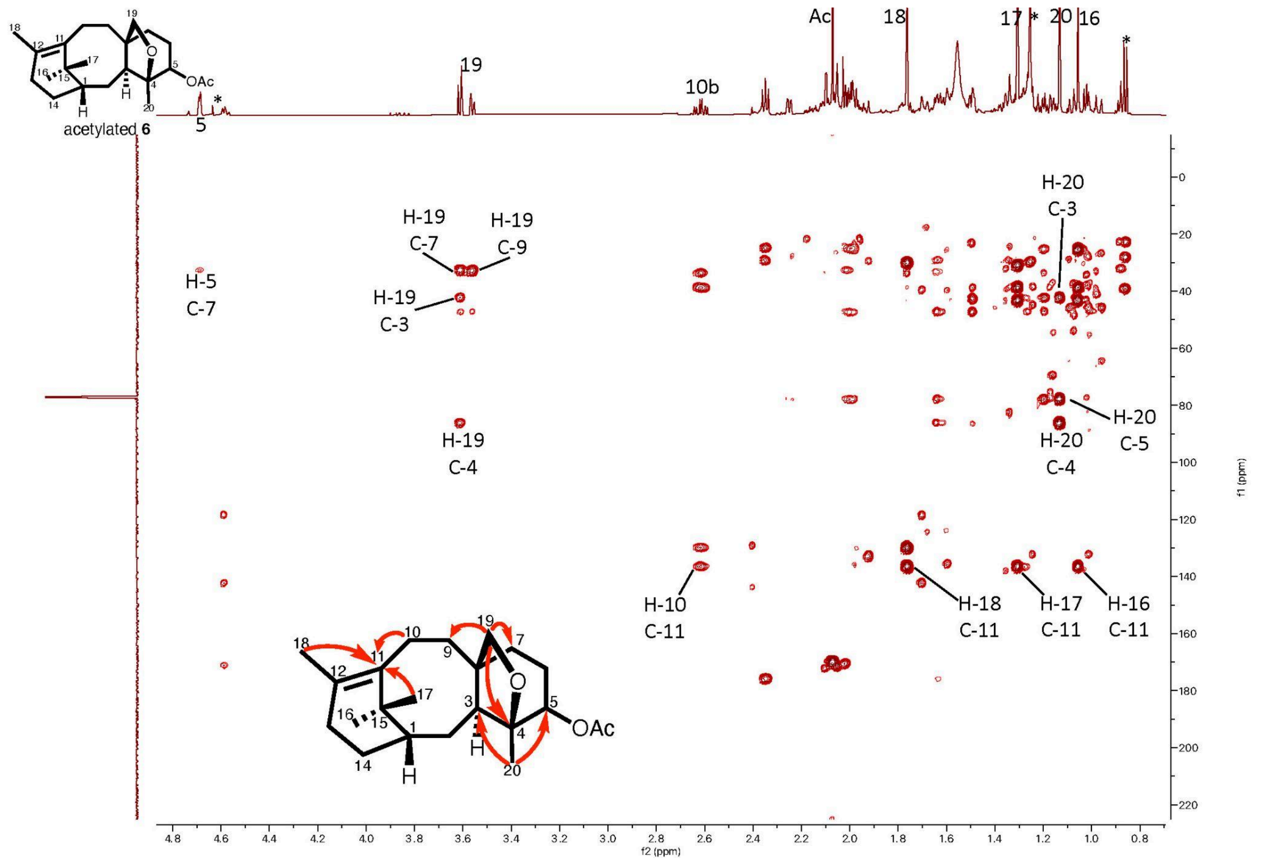

Supplementary Fig. 29. HMBC spectrum of acetylated 6 in $CDCl_3$ (600 Hz, n = 32).

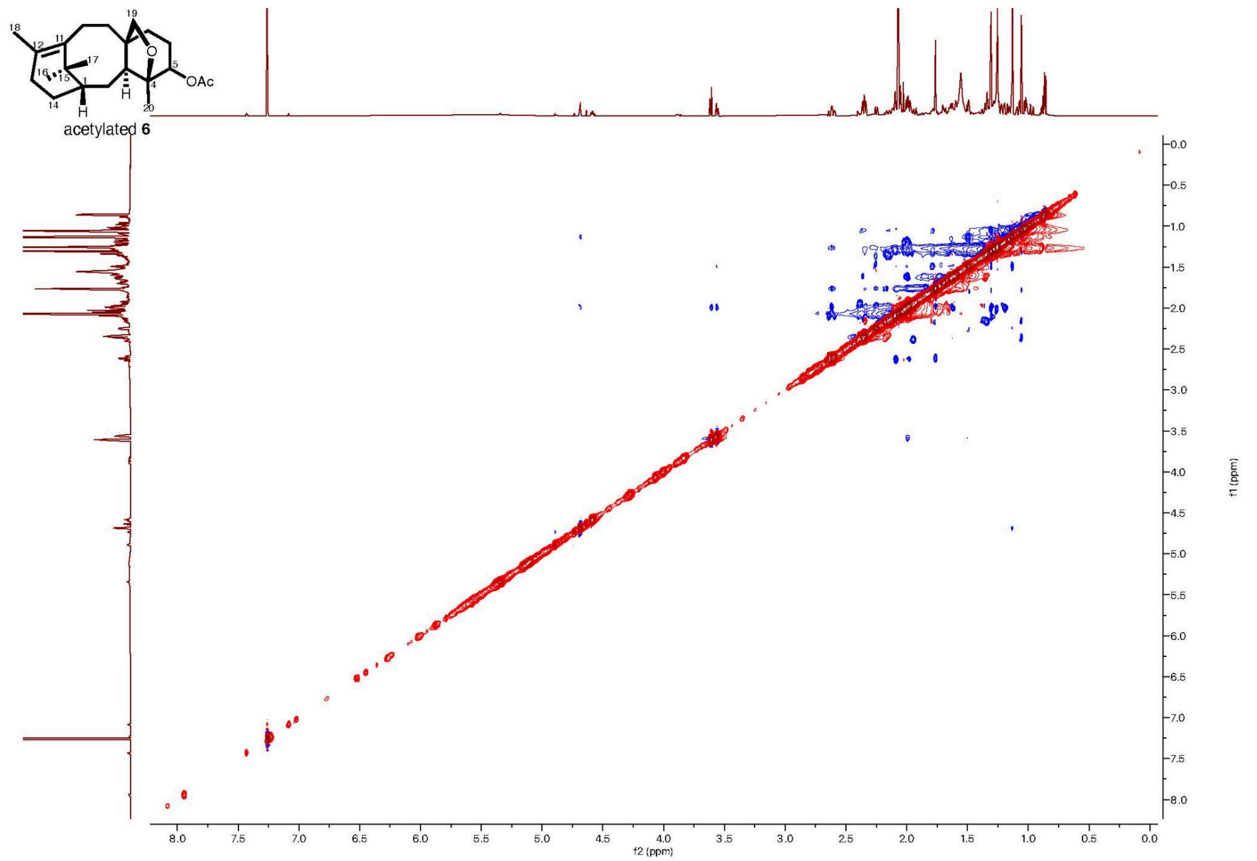

Supplementary Fig. 30. ROESY spectrum of acetylated 6 in $CDCl_3$ (600 Hz, n = 8).

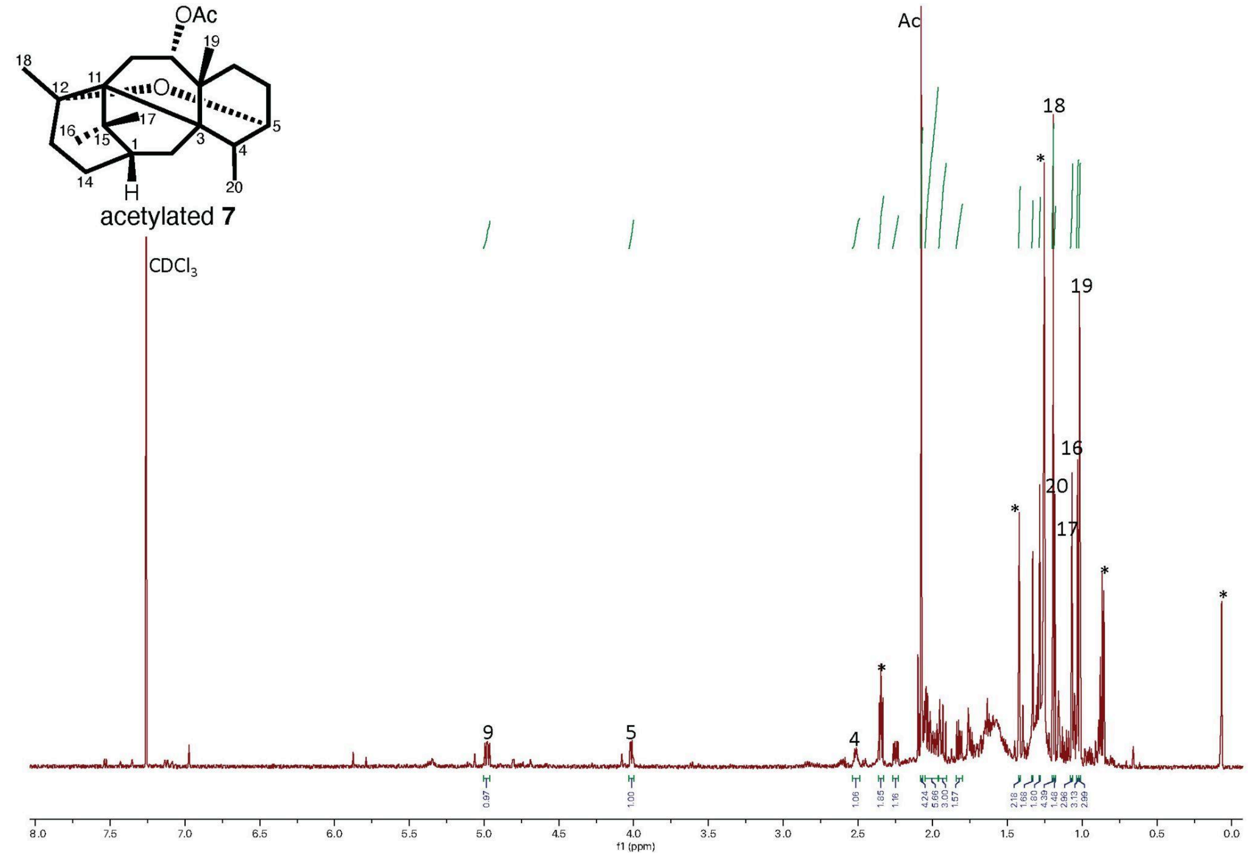

Supplementary Fig. 31. ^{1}H-NMR spectrum of acetylated 7 in $CDCl_3$ (600 Hz, n = 64). Asterisk indicates impurities.

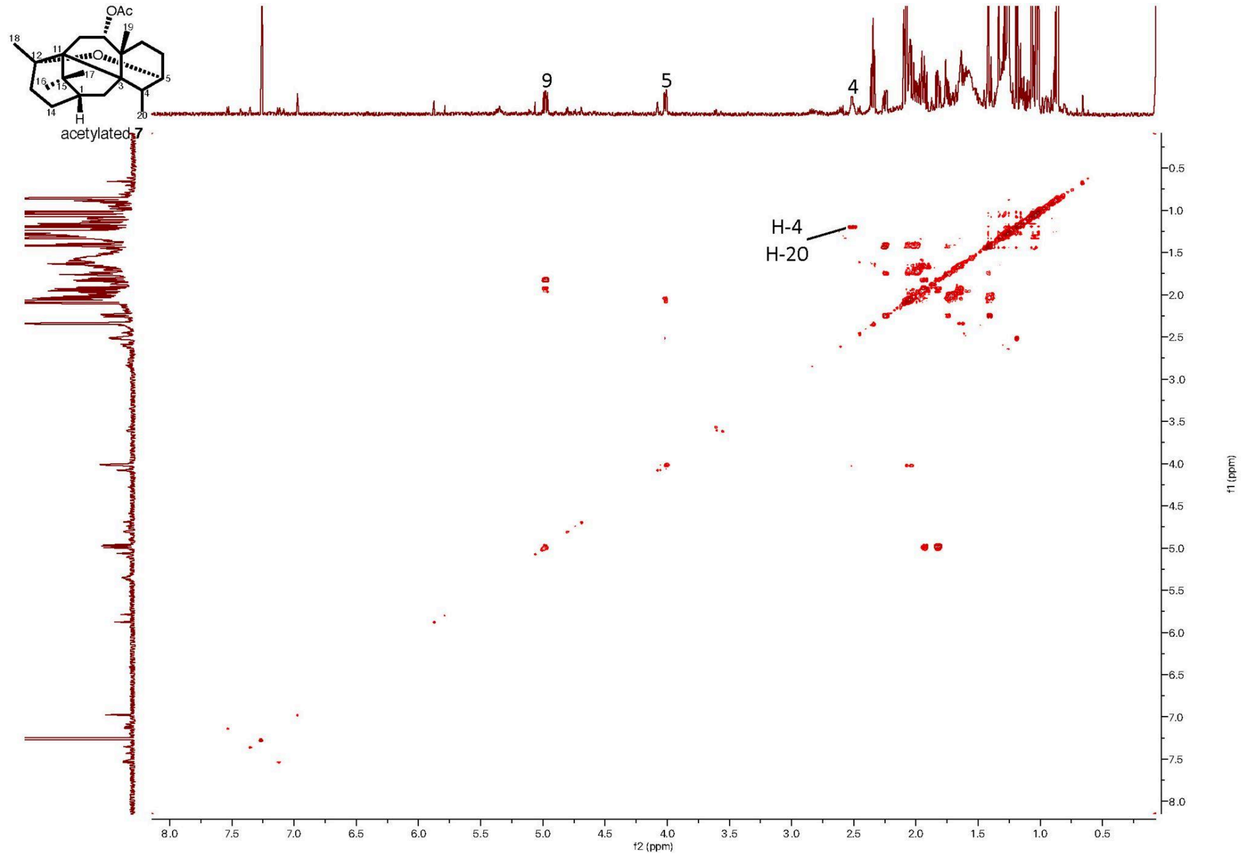

Supplementary Fig. 32. COSY spectrum of acetylated 7 in $CDCl_3$ (600 Hz, n = 4).

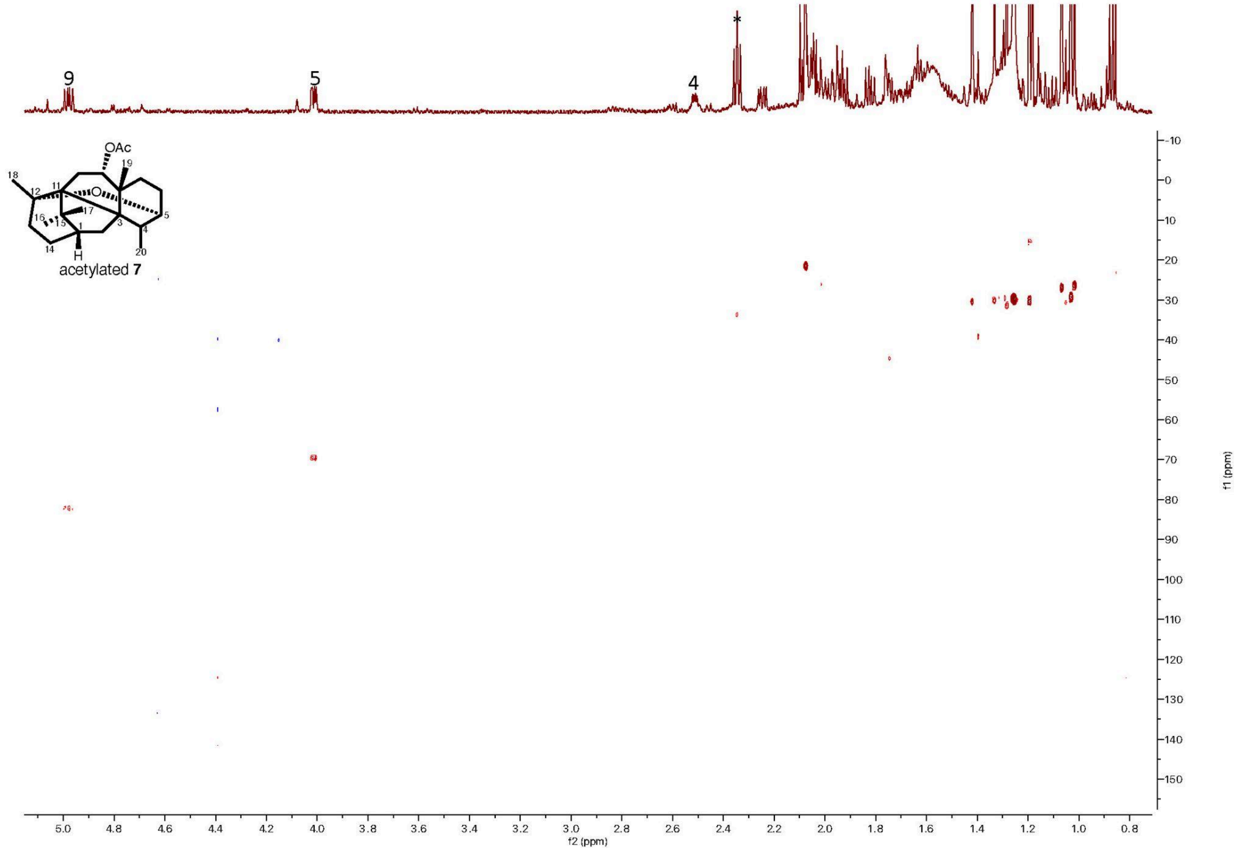

Supplementary Fig. 33. HSQC spectrum of acetylated 7 in $CDCl_3$ (600 Hz, n = 8).

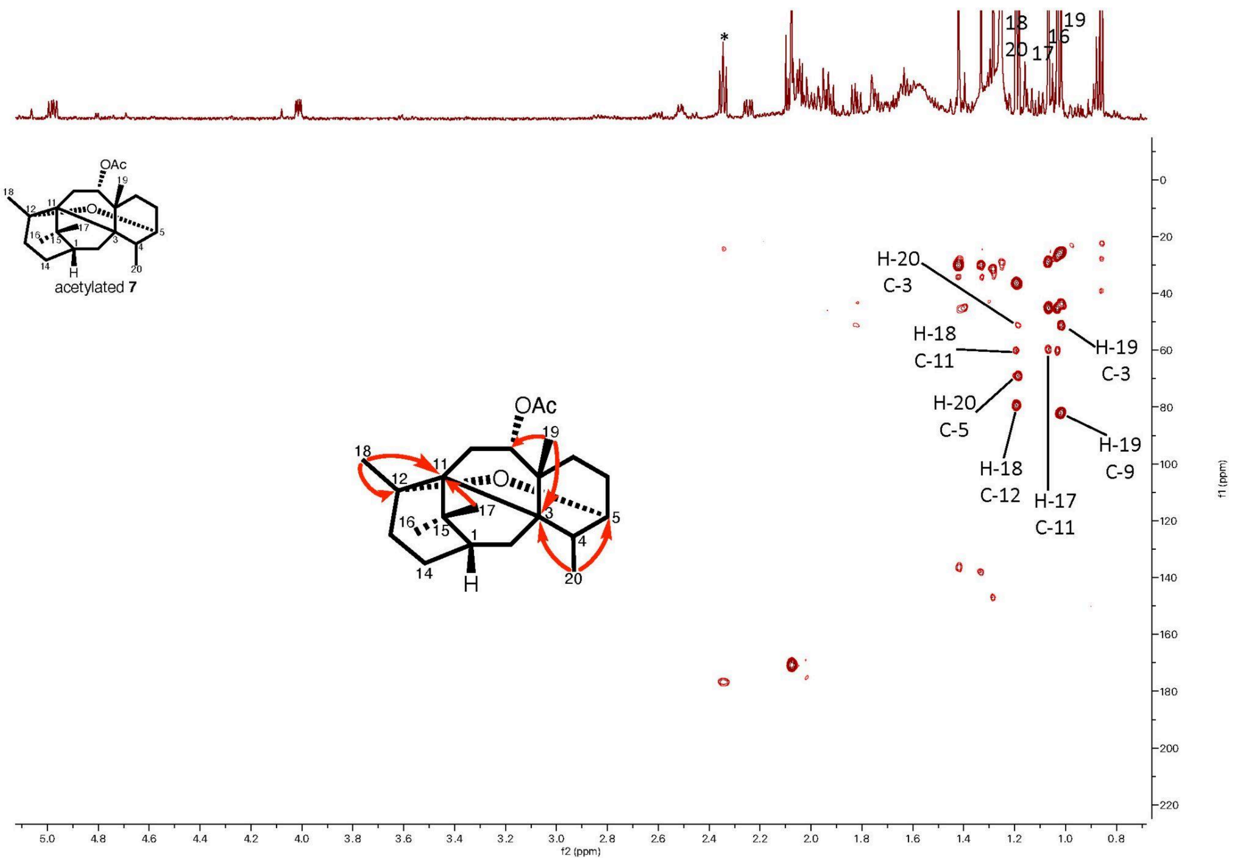

Supplementary Fig. 34. HMBC spectrum of acetylated 7 in $CDCl_3$ (600 Hz, n = 32).

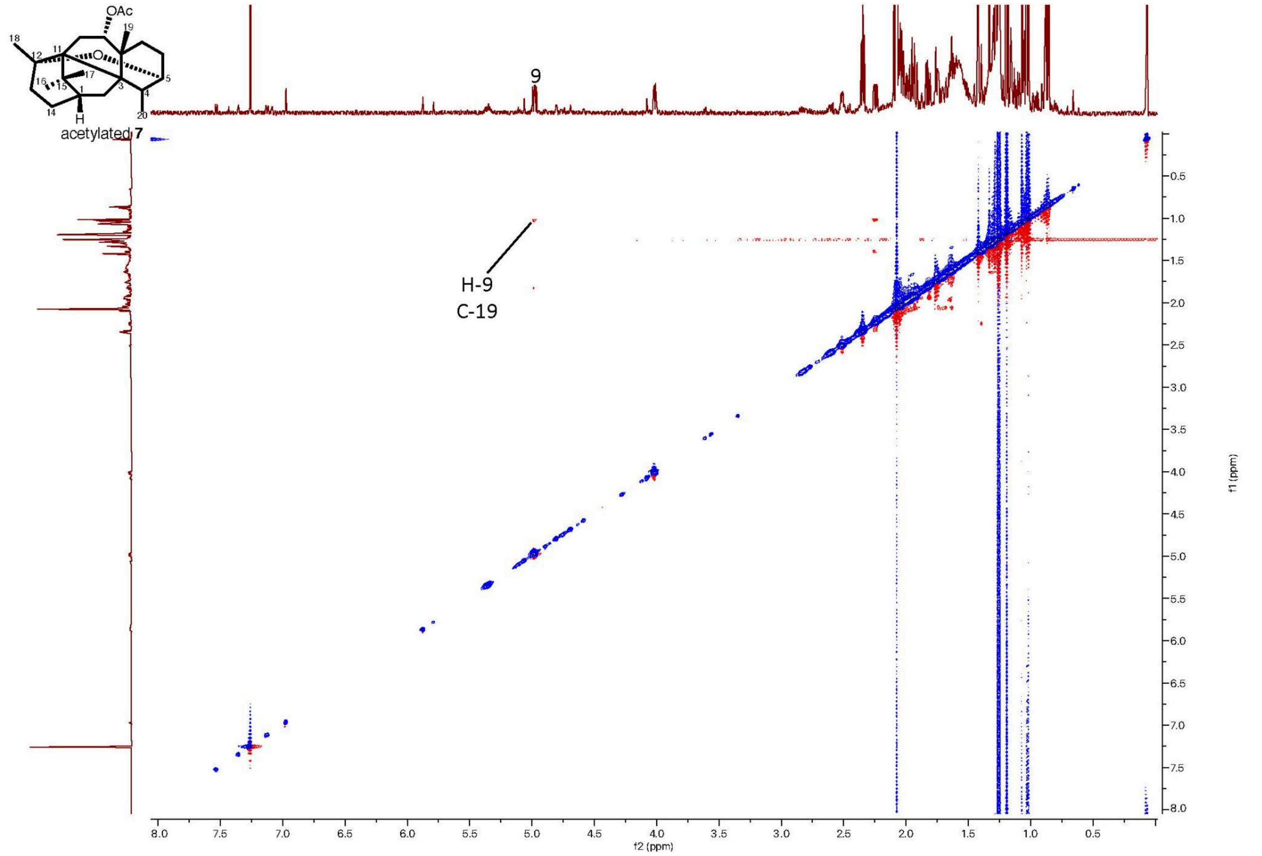

Supplementary Fig. 35. ROESY spectrum of acetylated 7 in $CDCl_3$ (600 Hz, n = 8).

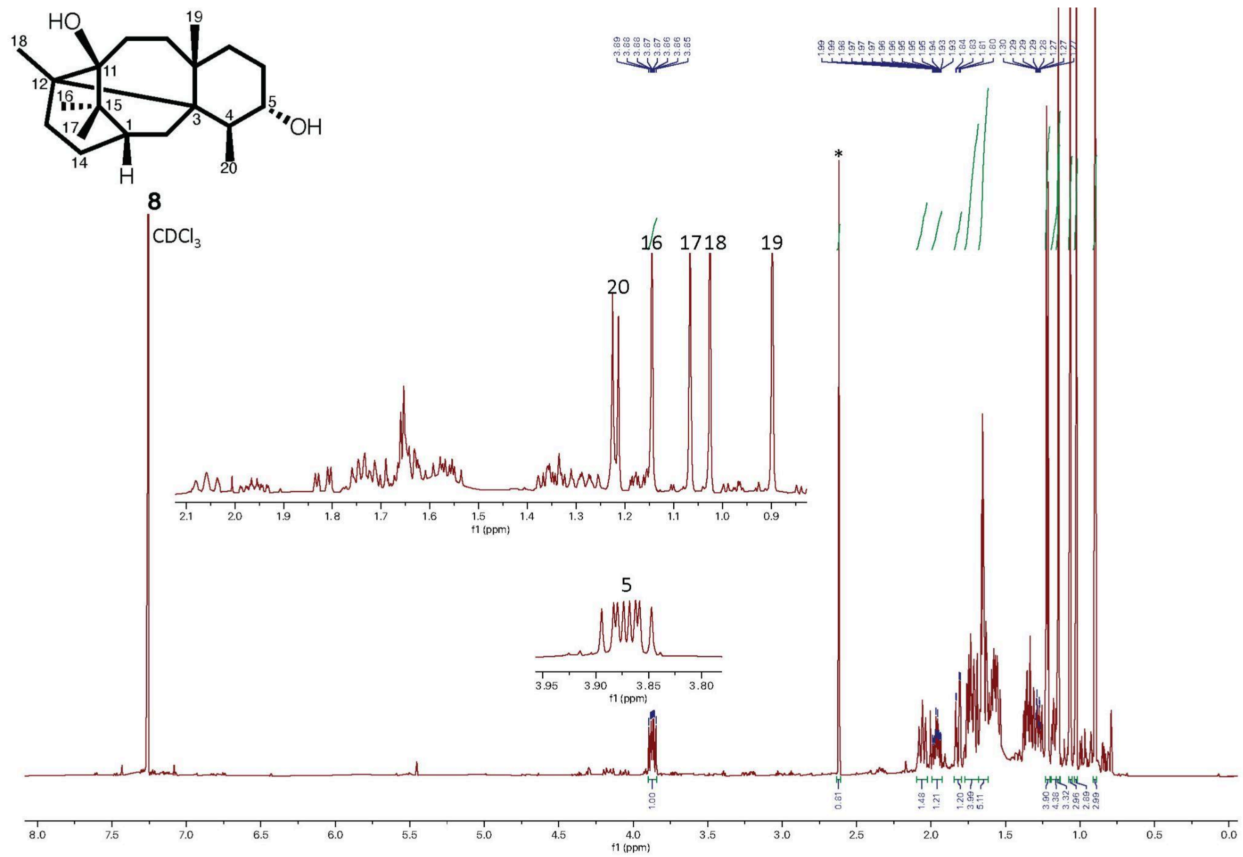

Supplementary Fig. 36. ^{1}H-NMR spectrum of compound 8 in $CDCl_3$ (600 Hz, n = 64). Asterisk indicates impurities.

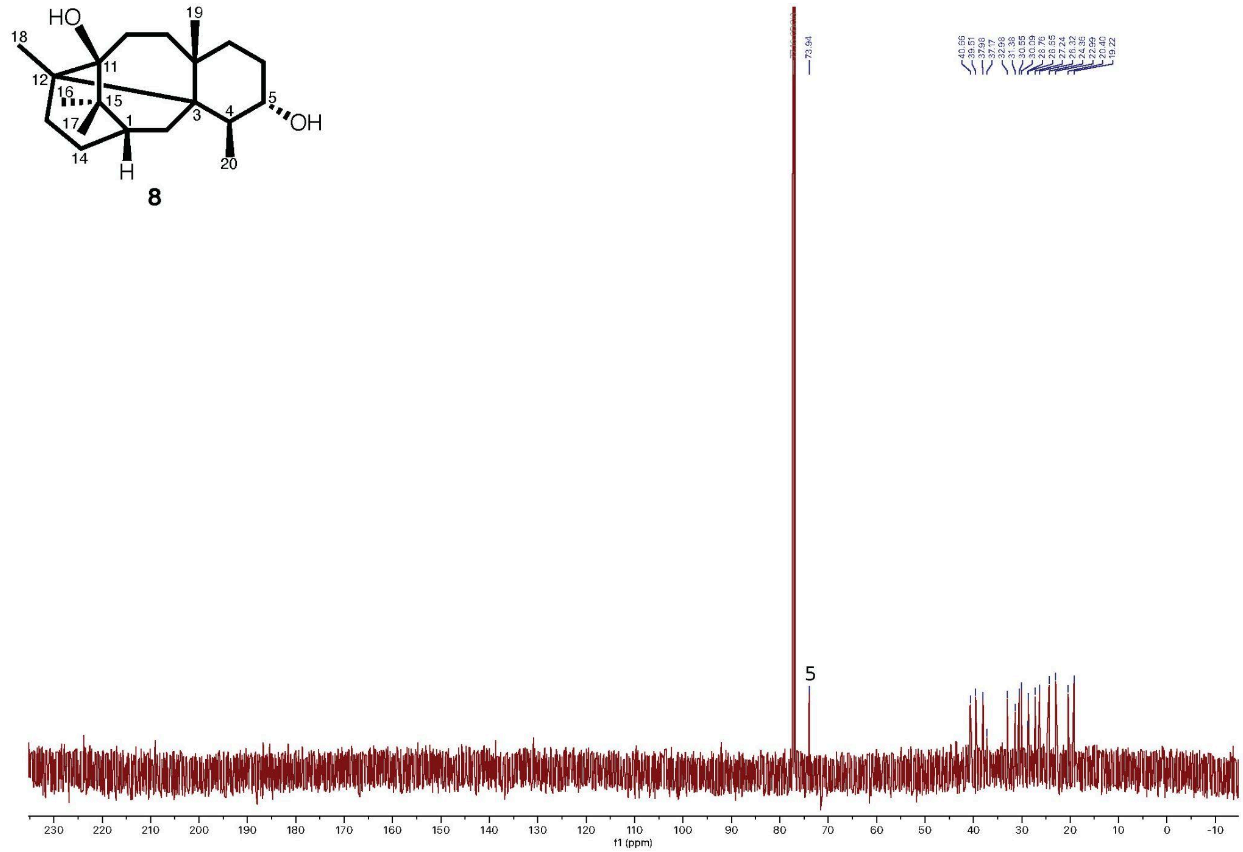

Supplementary Fig. 37. ^{13}C-NMR spectrum of compound 8 in $CDCl_3$ (600 Hz, n = 1024).

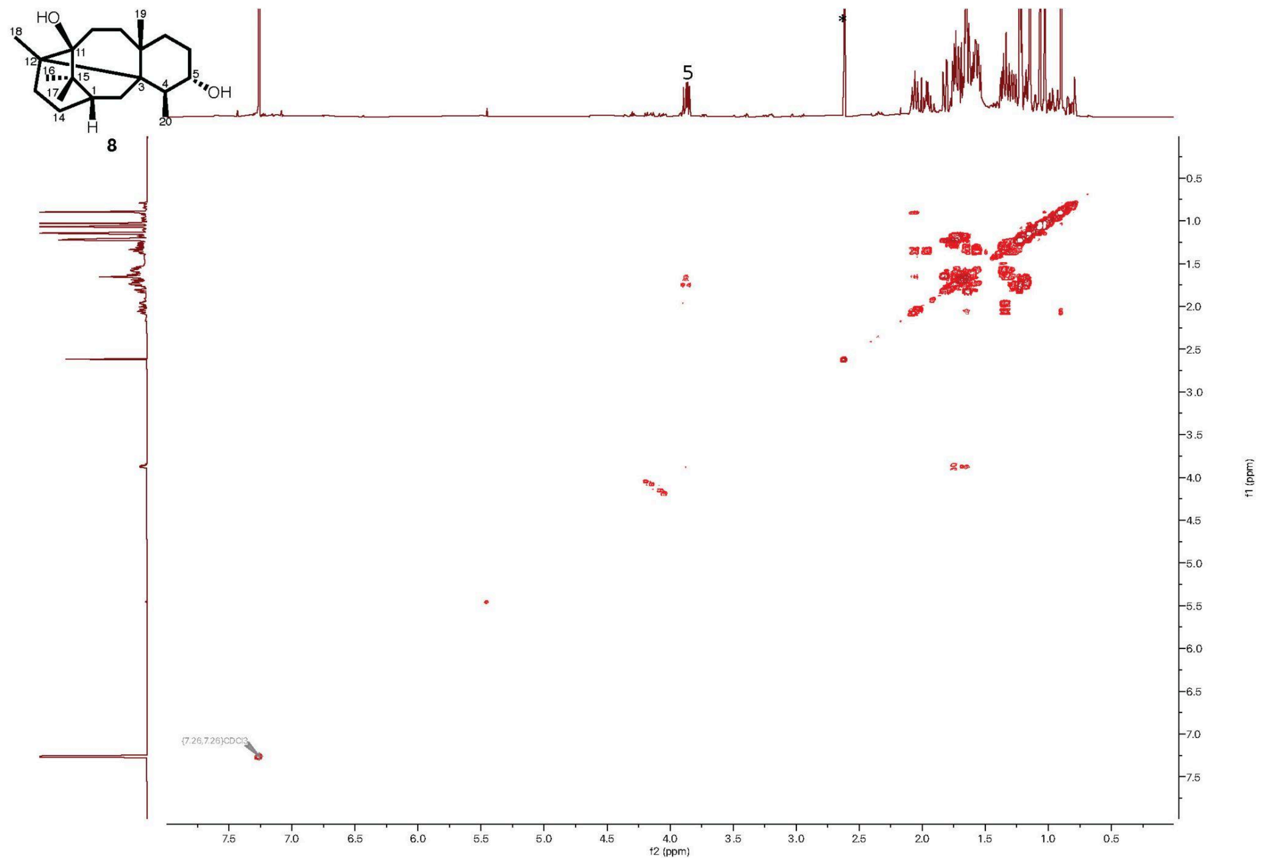

Supplementary Fig. 38. COSY spectrum of compound 8 in $CDCl_3$ (600 Hz, n = 8).

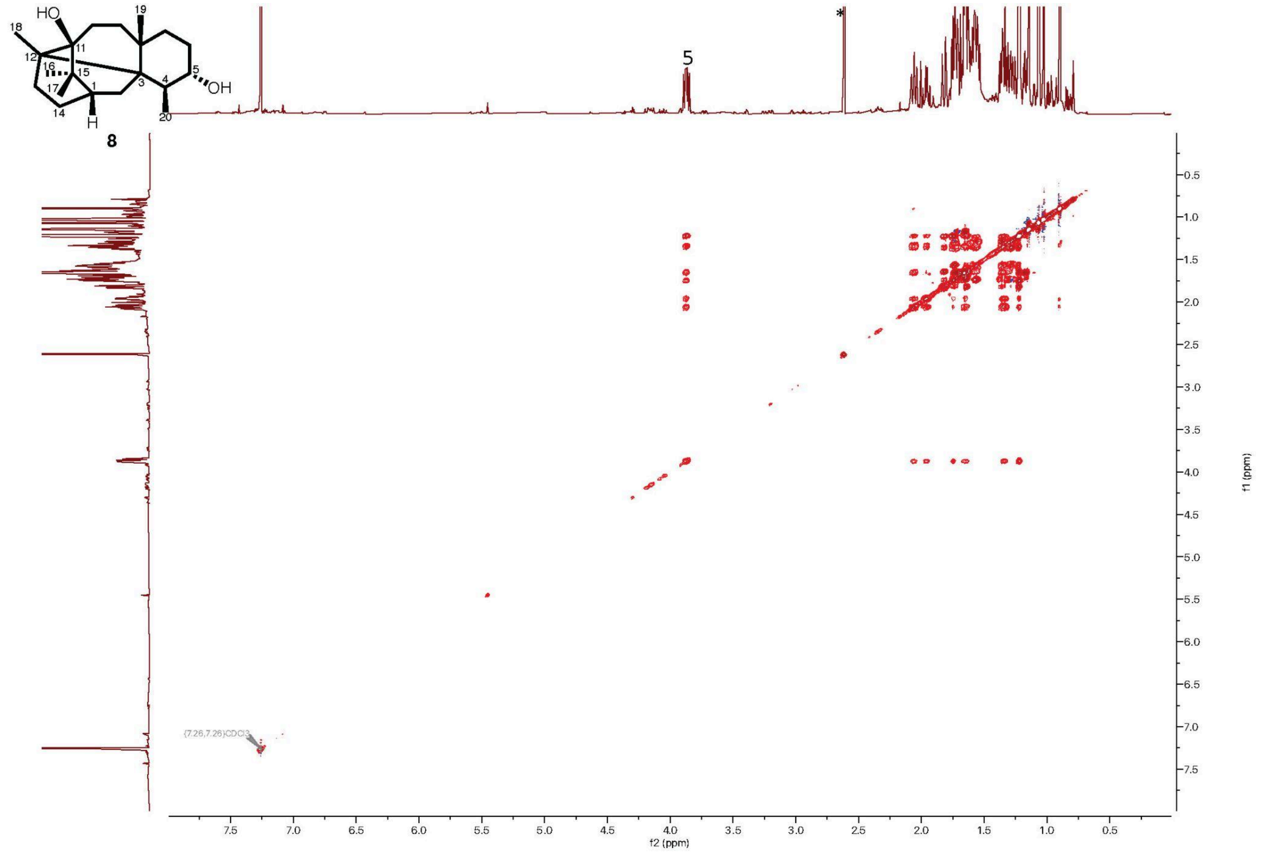

Supplementary Fig. 39. TOCSY spectrum of compound 8 in $CDCl_3$ (600 Hz, n = 4).

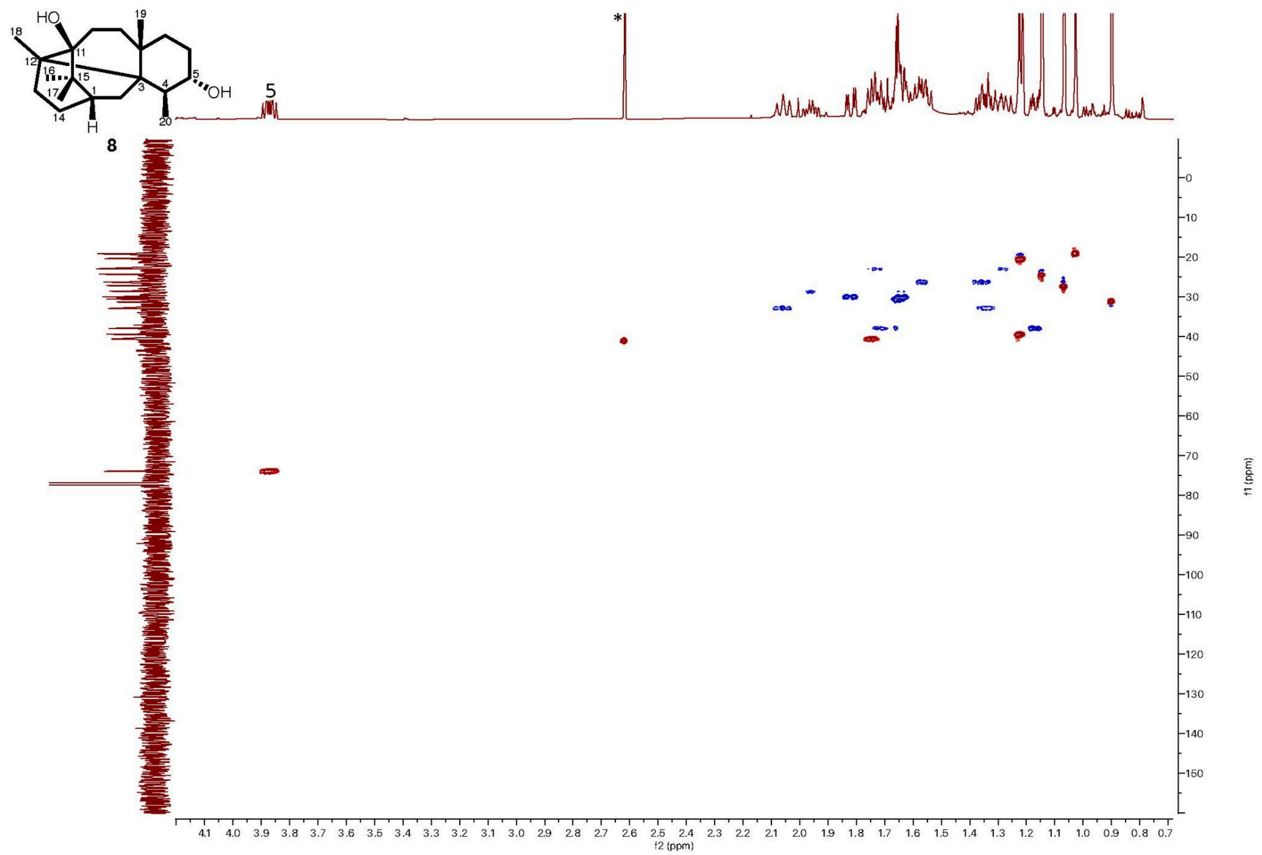

Supplementary Fig. 40. HSQC spectrum of compound 8 in $CDCl_3$ (600 Hz, n = 8).

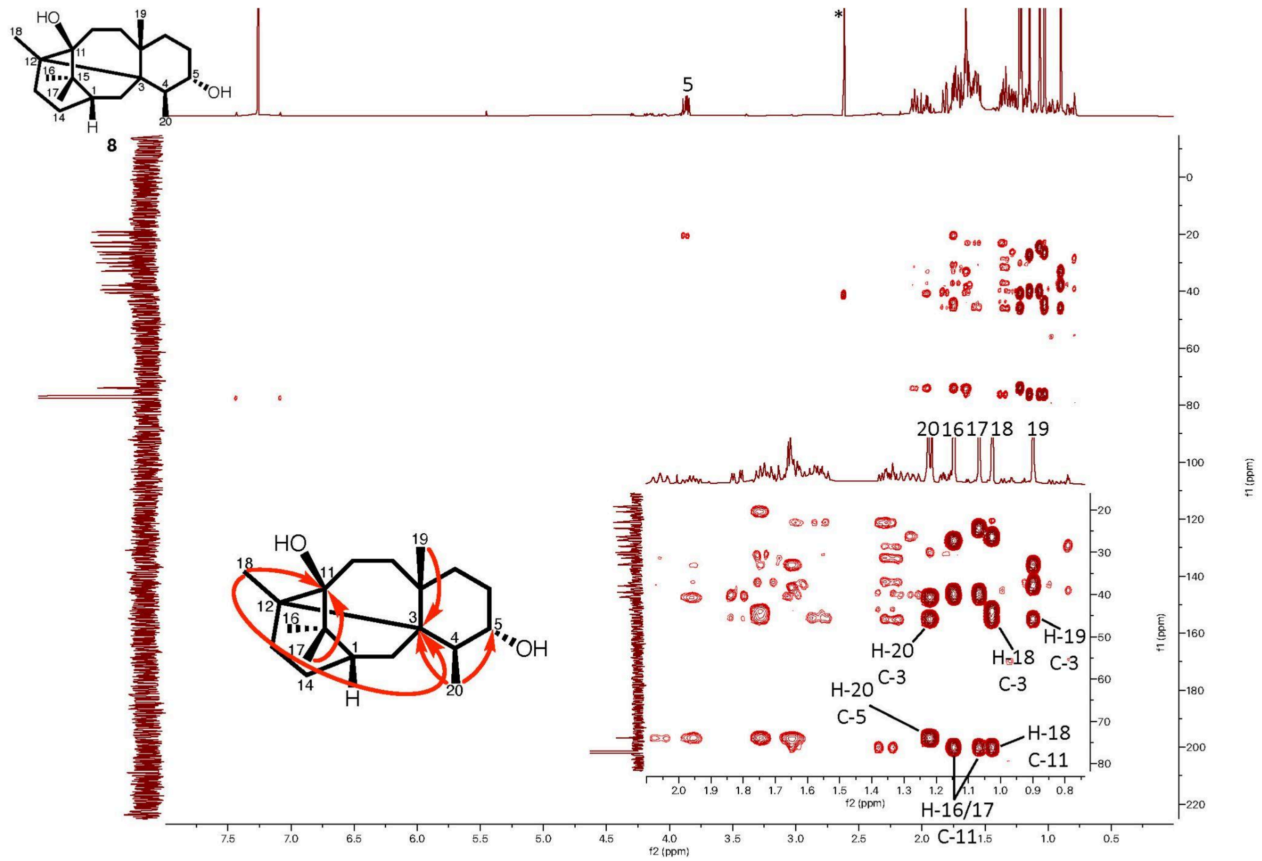

Supplementary Fig. 41. HMBC spectrum of compound 8 in $CDCl_3$ (600 Hz, n = 32).

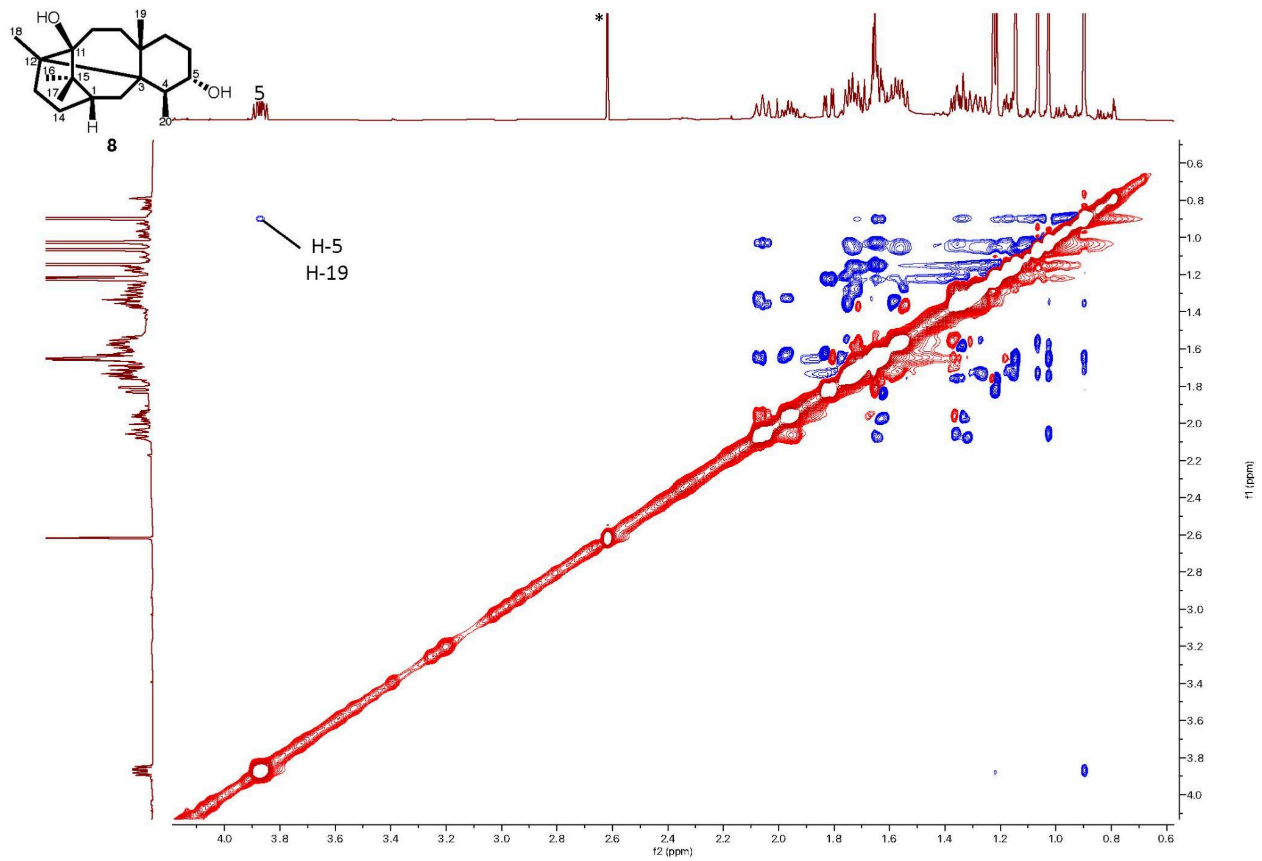

Supplementary Fig. 42. ROESY spectrum of compound 8 in $CDCl_3$ (600 Hz, n = 8).

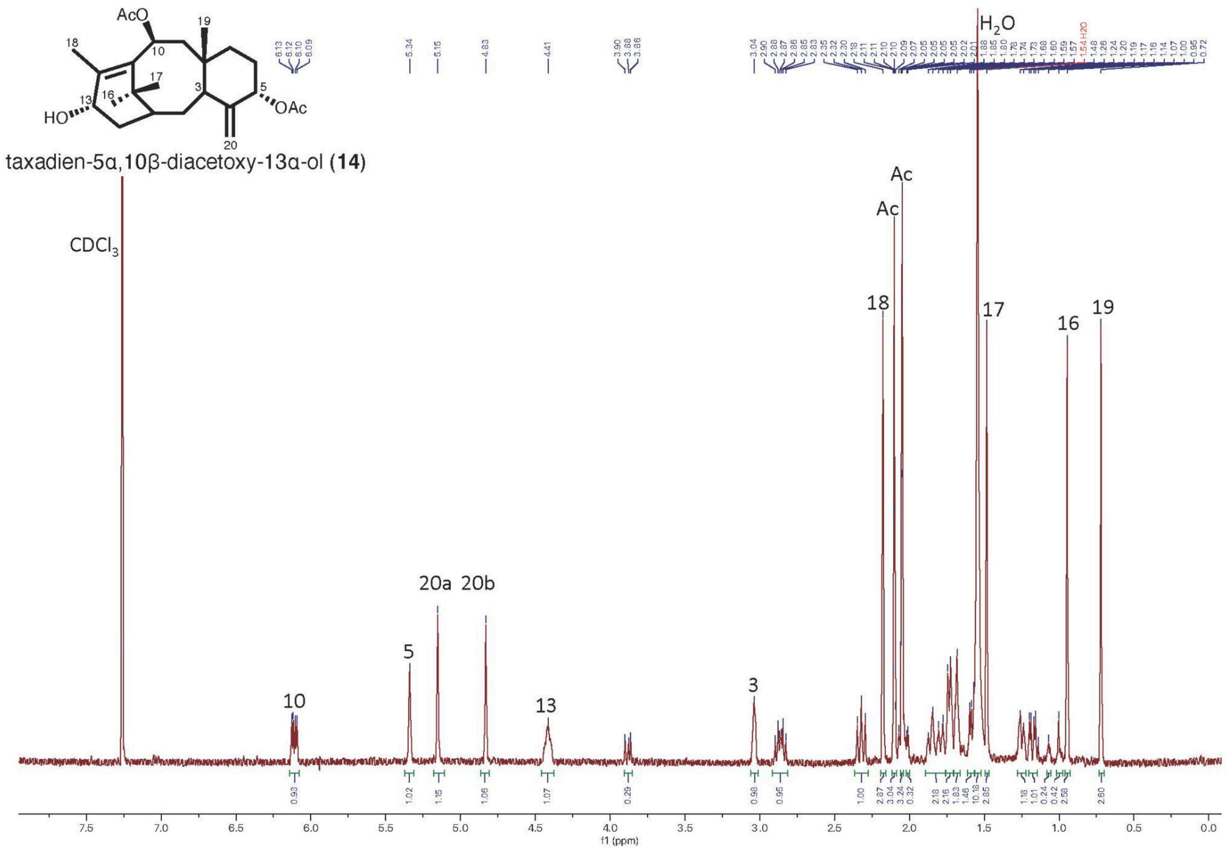

Supplementary Fig. 43. ^{1}H-NMR spectrum of 5α,10β-diacetoxy-13α-ol (14) in $CDCl_3$ (500 Hz, n = 64).

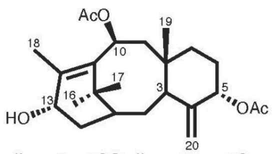

taxadien-5α,10β-diacetoxy-13α-ol (**14**)

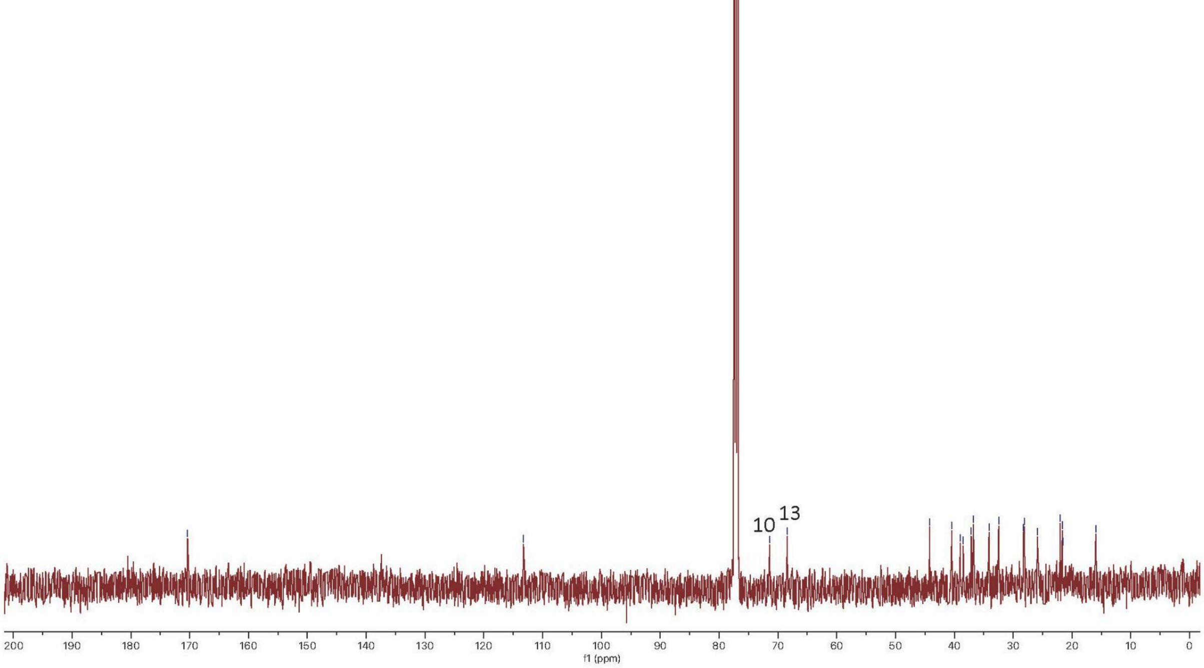

Supplementary Fig. 44. ^{13}C-NMR spectrum of 5α,10β-diacetoxy-13α-ol (14) in $CDCl_3$ (500 Hz, n = 2048).

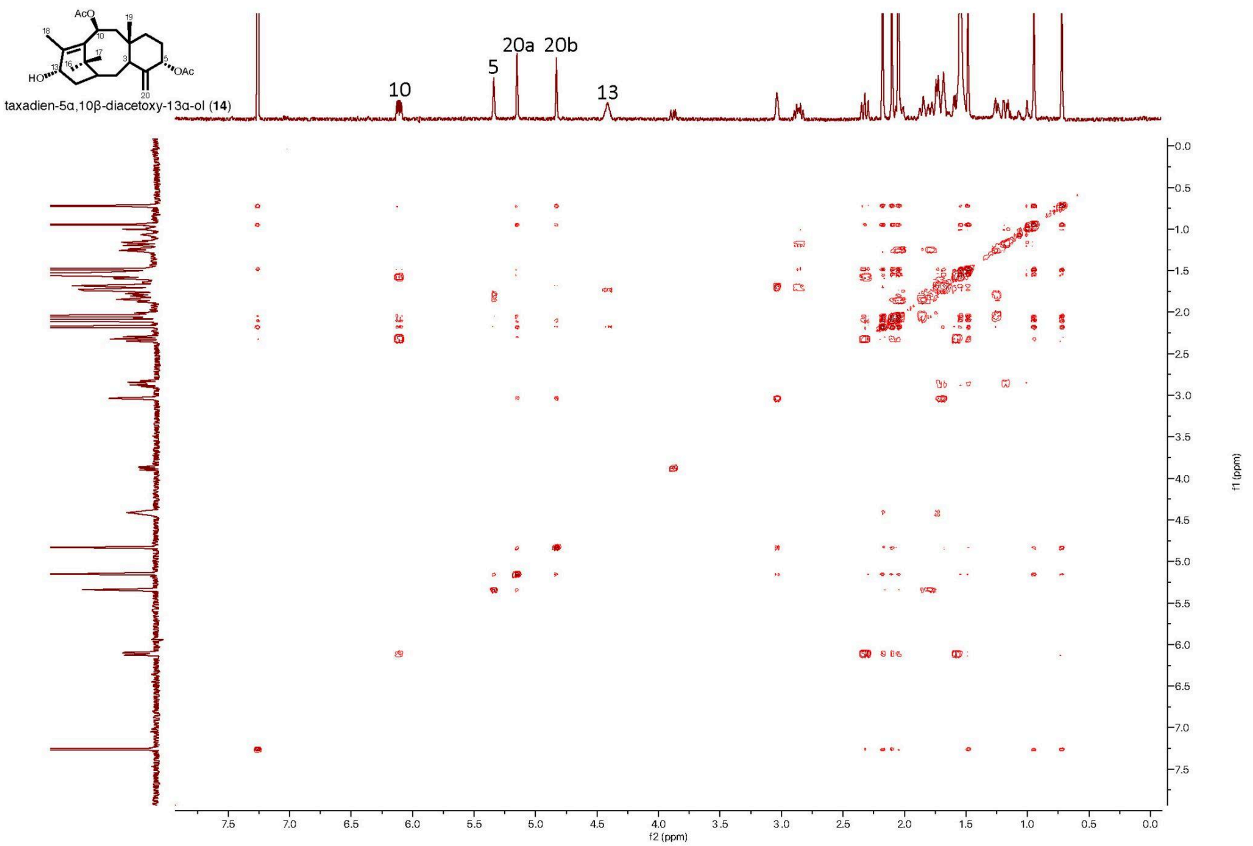

Supplementary Fig. 45. COSY spectrum of 5α,10β-diacetoxy-13α-ol (14) in $CDCl_3$ (500 Hz, n = 4).

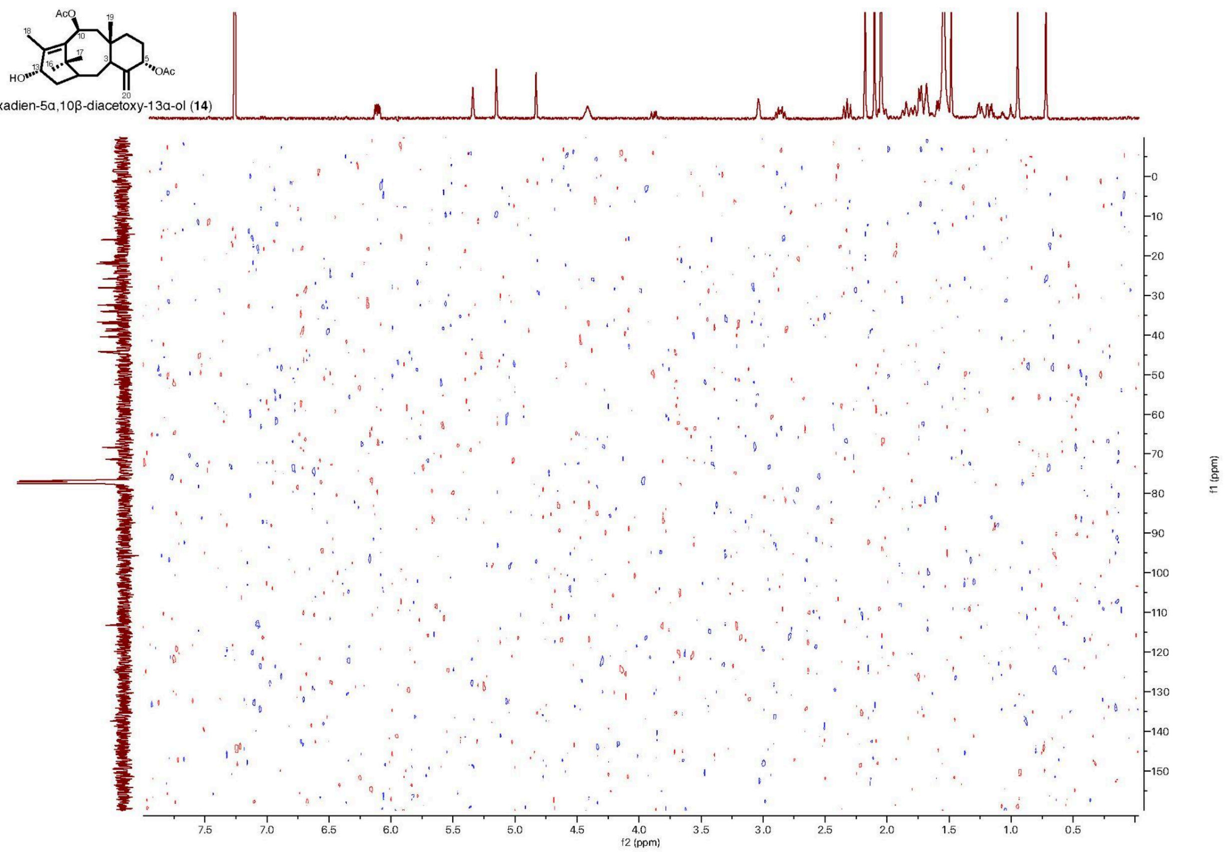

Supplementary Fig. 46. HSQC spectrum of 5α,10β-diacetoxy-13α-ol (14) in $CDCl_3$ (500 Hz, n = 8).

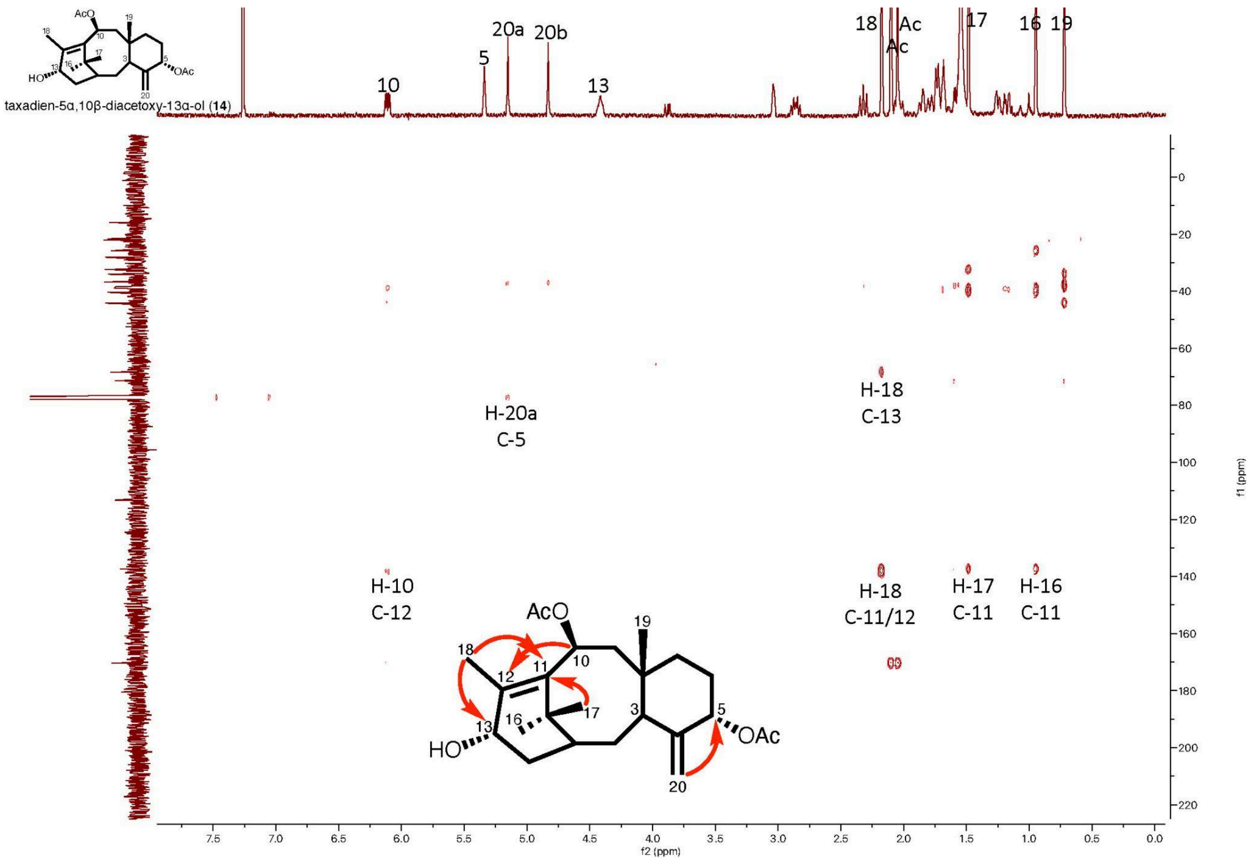

Supplementary Fig. 47. HMBC spectrum of 5α,10β-diacetoxy-13α-ol (14) in $CDCl_3$ (500 Hz, n = 32).

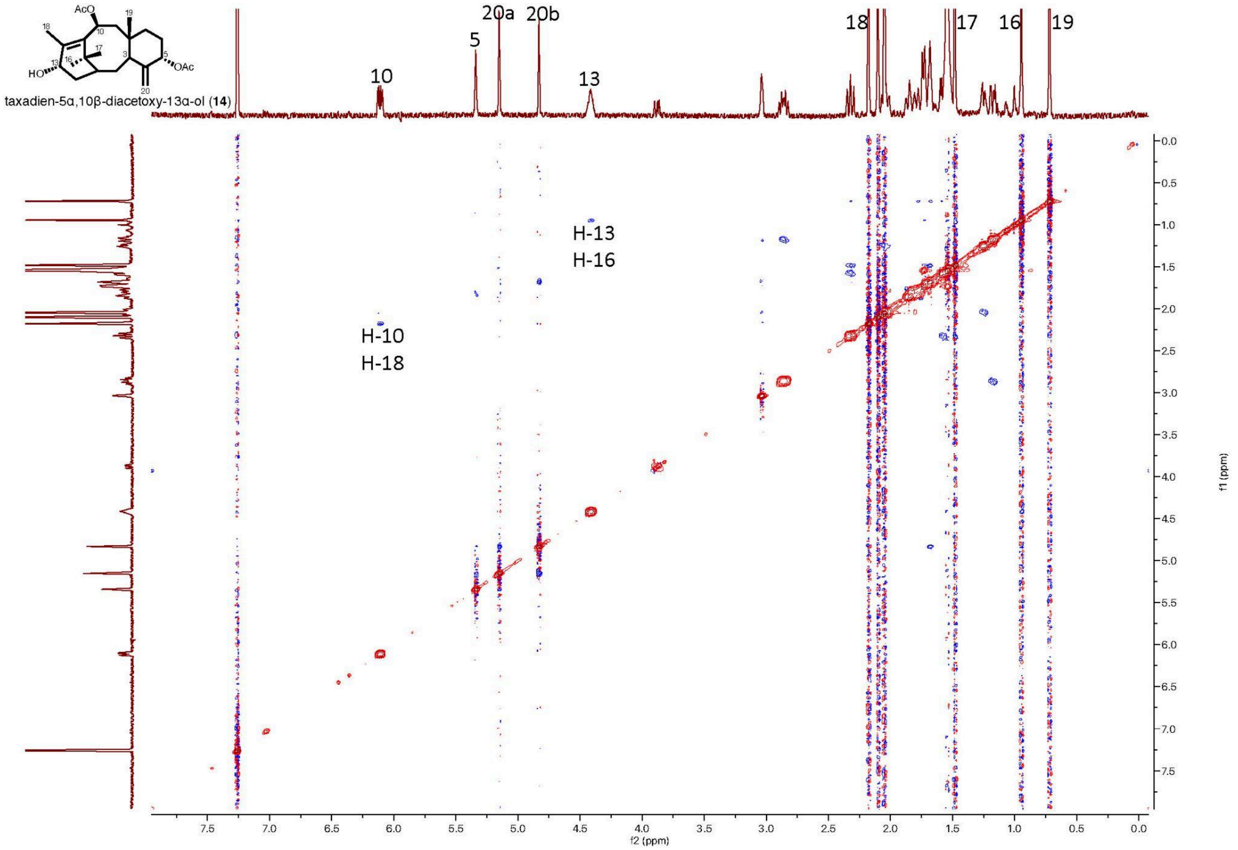

Supplementary Fig. 48. ROESY spectrum of 5α,10β-diacetoxy-13α-ol (14) in $CDCl_3$ (500 Hz, n = 8).

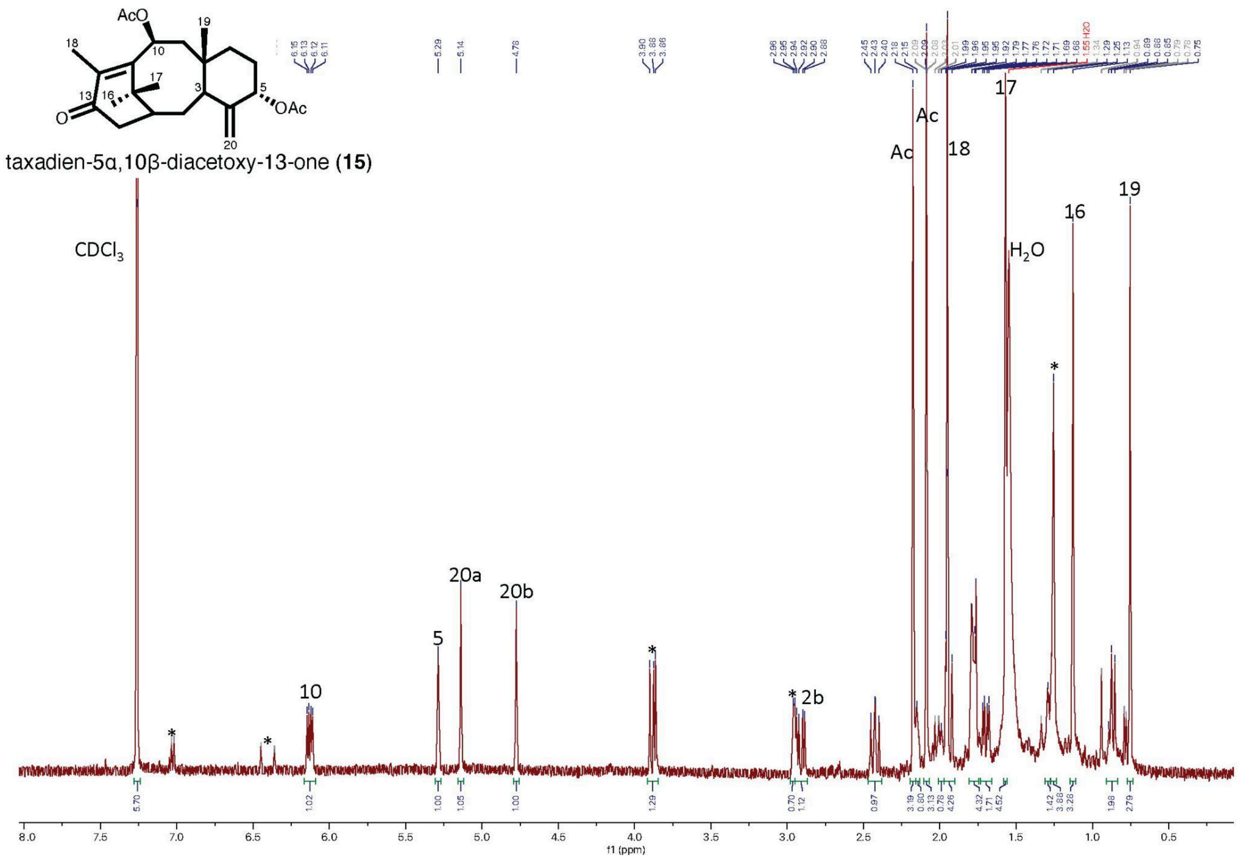

Supplementary Fig. 49. ^{1}H-NMR spectrum of 5α,10β-diacetoxy-13α-one (15) in $CDCl_3$ (500 Hz, n = 64). Asterisk indicates impurities.

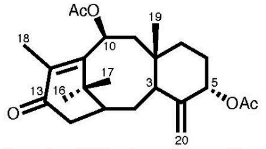

taxadien-5α,10β-diacetoxy-13-one (**15**)

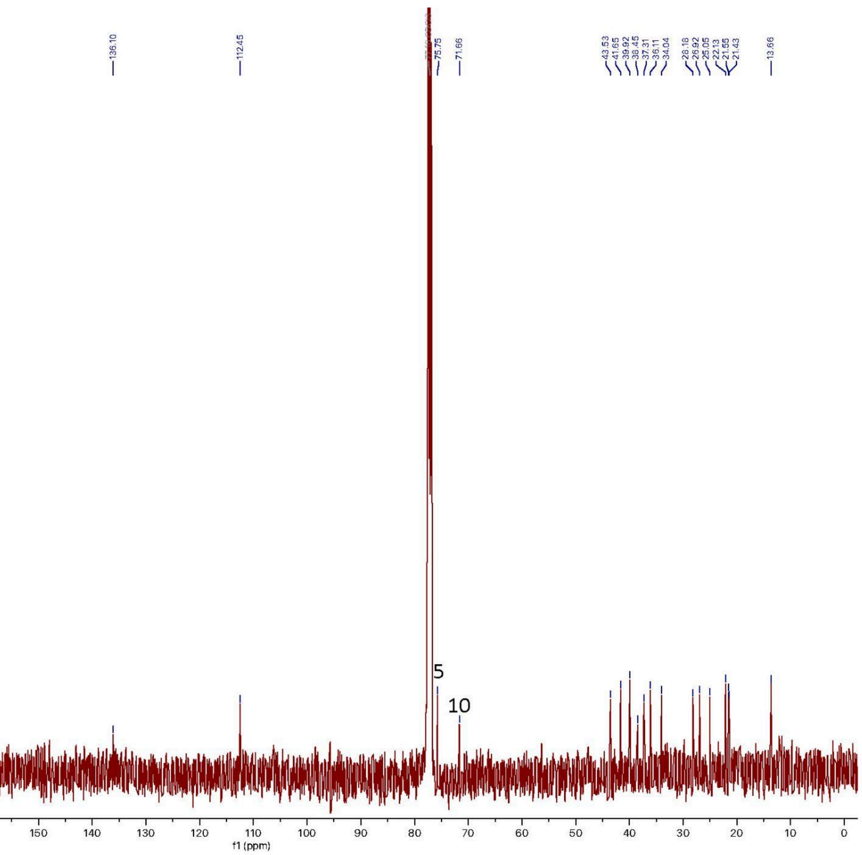

Supplementary Fig. 50. ^{13}C-NMR spectrum of 5α,10β-diacetoxy-13α-one (15) in $CDCl_3$ (500 Hz, n = 4096).

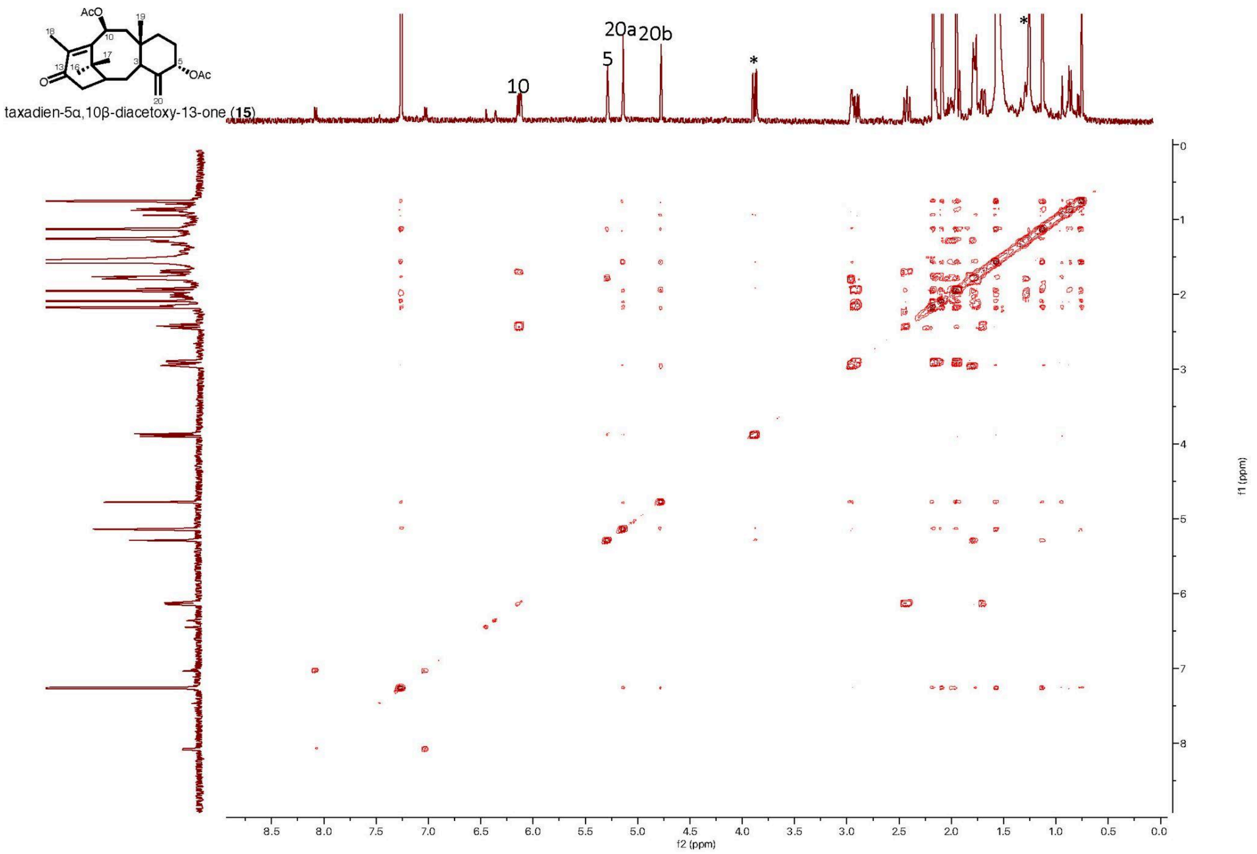

Supplementary Fig. 51. COSY spectrum of 5α,10β-diacetoxy-13α-one (15) in $CDCl_3$ (500 Hz, n = 2).

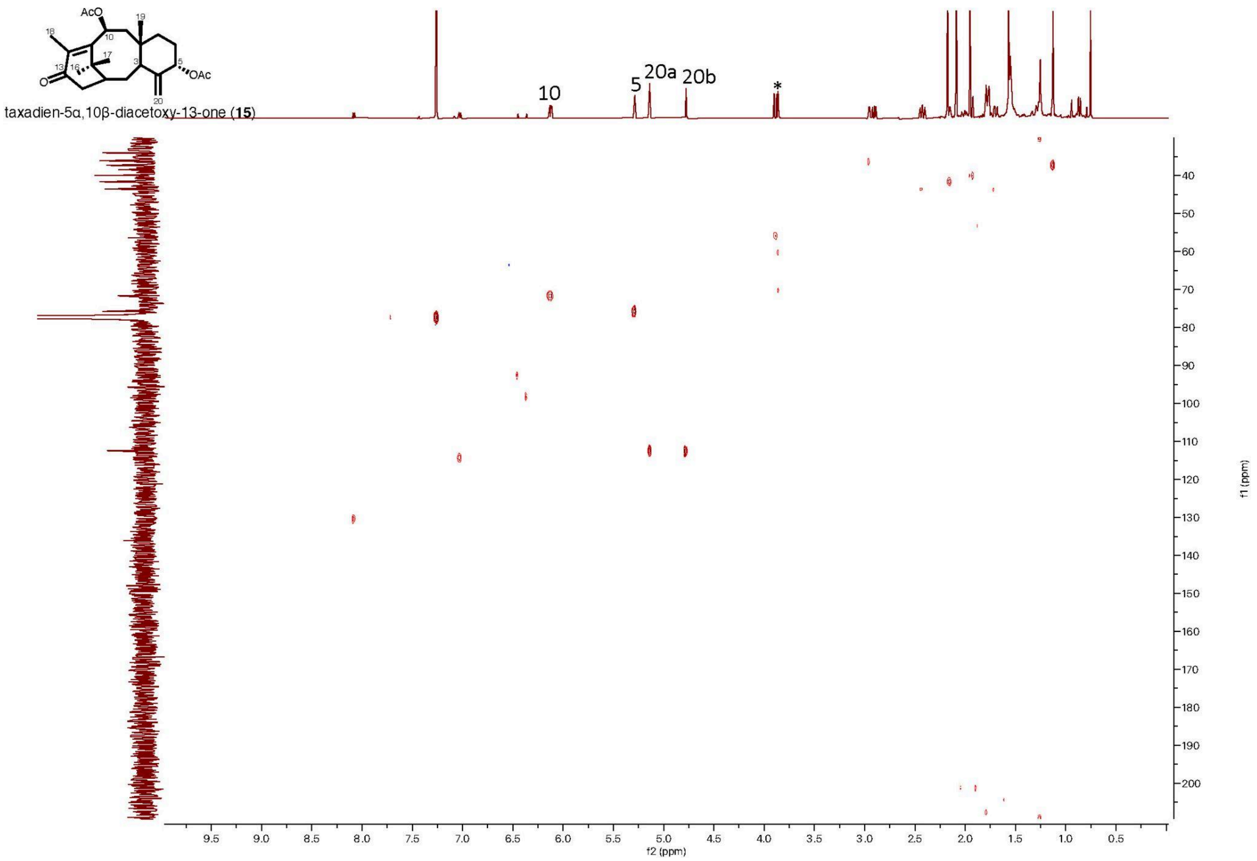

Supplementary Fig. 52. HSQC spectrum of 5α,10β-diacetoxy-13α-one (15) in $CDCl_3$ (600 Hz, n = 8).

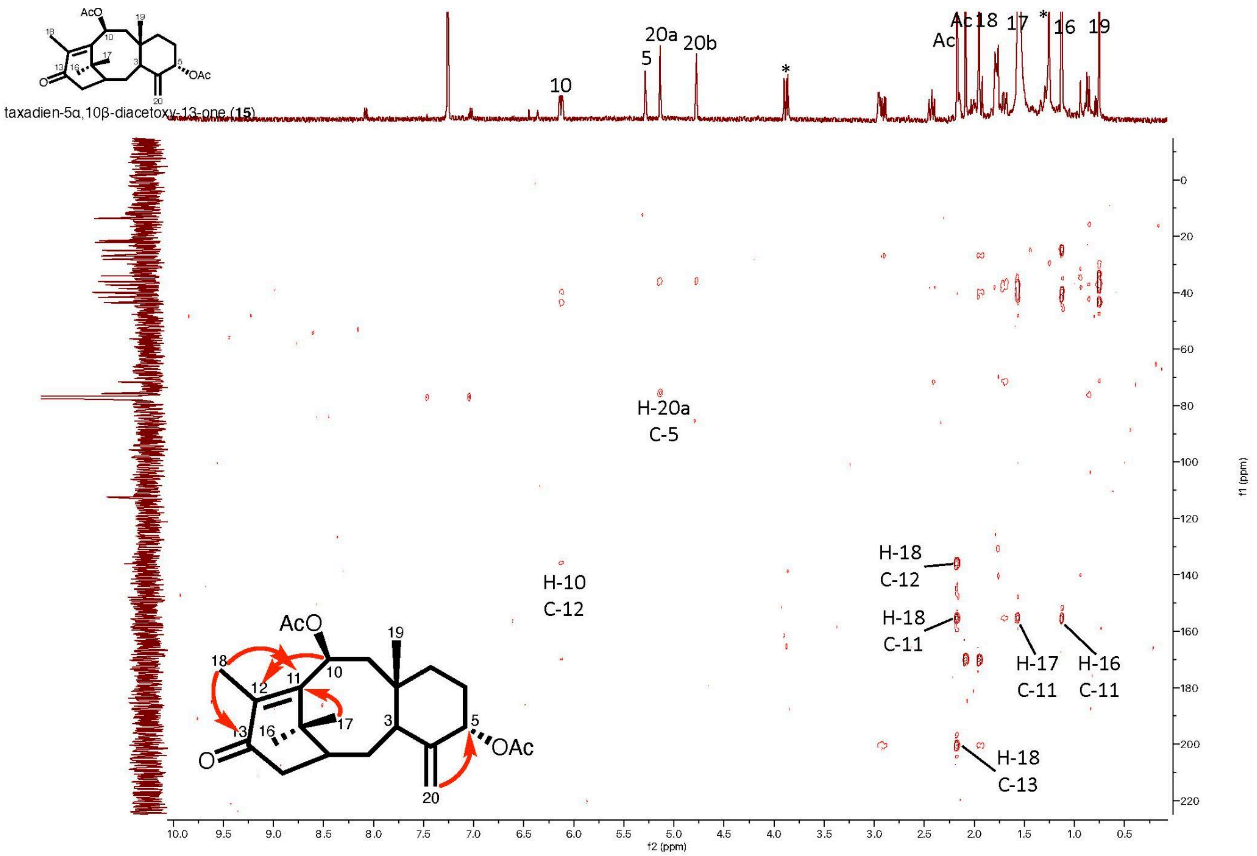

Supplementary Fig. 53. HMBC spectrum of 5α,10β-diacetoxy-13α-one (15) in $CDCl_3$ (500 Hz, n = 64).

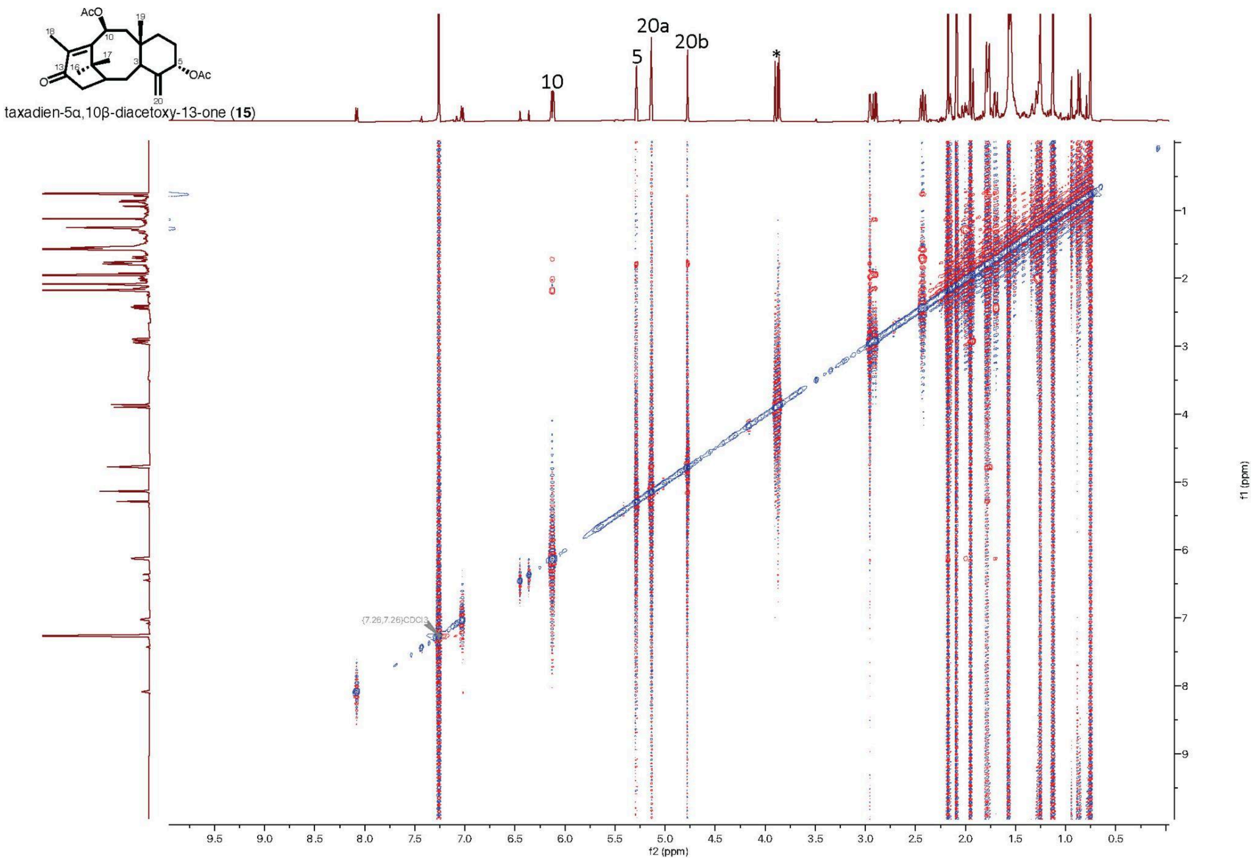

Supplementary Fig. 54. ROESY spectrum of 5α,10β-diacetoxy-13α-one (15) in $CDCl_3$ (500 Hz, n = 8).

Supplementary Tables

Supplementary Table 1. Reported product profile of T5αH in different systems.

Host	iso-OCT	OCT	Taxadien-5α-ol	Diols	Others	Year/Reference
S. cerevisiae			✓	✓		2004[10] (Original discovery of T5αH)
Spodoptera frugiperda (armyworm)			✓			
N. sylvestris trichome		✓				2008[11]
S. cerevisiae microsome		✓				
E. coli	✓	✓	?			2010[12]
E. coli	✓	✓	✓	✓	✓	2014[9]
E. coli (TS) + *S. cerevisiae* (T5αH) consortium		✓	✓		✓	2015[13]
In vitro lipid nanodisc		✓	✓	✓	✓	2016[7]
Yarrowia lipolytica	✓	✓	✓	✓	✓	2016[8]
E. coli	✓	✓	✓		✓	2018[5]
N. benthamiana	✓	✓	✓			2019[14]
S. cerevisiae	✓	✓	✓	✓	✓	2021[15]
S. cerevisiae microsome	✓	✓	✓			2021[16]
S. cerevisiae	✓	✓	✓	✓		2022[17]

Supplementary Table 2. Mass spectra of all compounds in this study.

Compound	Molecular formula	Calc. mass	Retention time (min)	Mass spectra
taxadiene (**1**)	$C_{20}H_{32}$	272.2504	10.61 (GCMS)	GCMS: 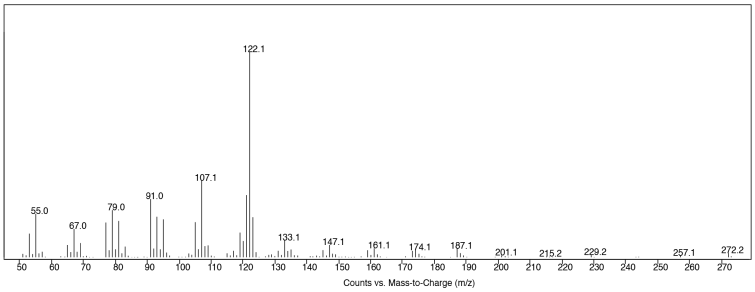
taxadien-5α-ol (**2**)	$C_{20}H_{32}O$	288.2453	12.17 (GCMS)	GCMS: 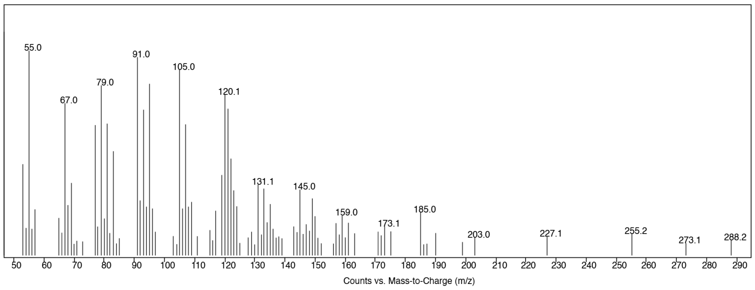
OCT (**3**)	$C_{20}H_{32}O$	288.2453	11.89 (GCMS)	GCMS: 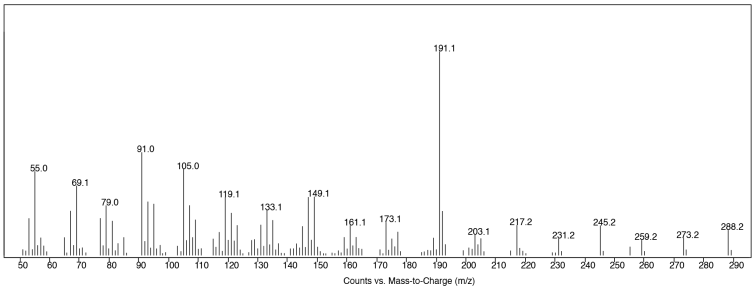
iso-OCT (**4**)	$C_{20}H_{32}O$	288.2453	11.33 (GCMS)	GCMS: 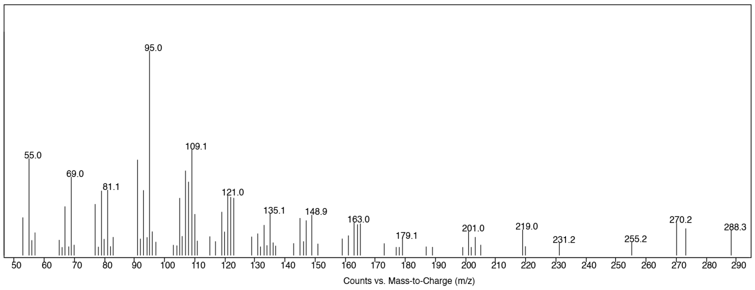

**5**	$C_{20}H_{32}O$	288.2453	12.90 (GCMS)	GCMS: 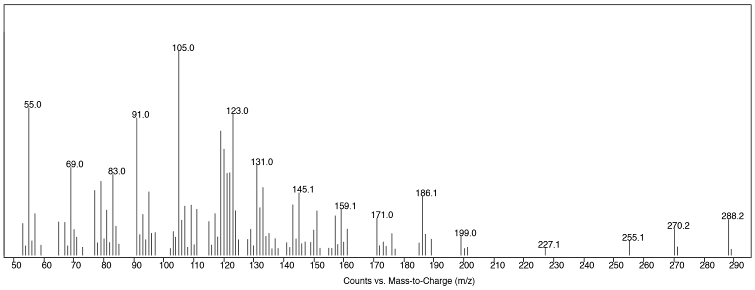
**6**	$C_{20}H_{32}O_2$	304.2402	13.90 (GCMS)	GCMS: 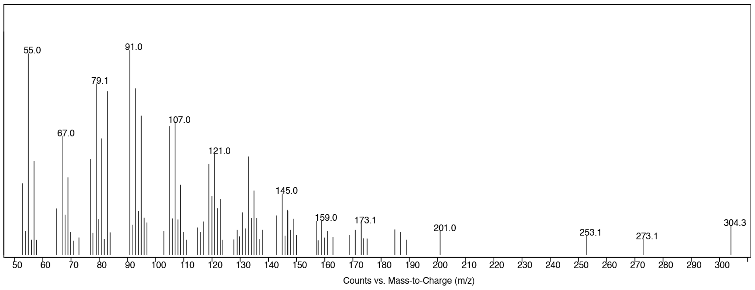
acetylated **6**	$C_{22}H_{34}O_3$	346.2508	15.02 (GCMS)	GCMS: 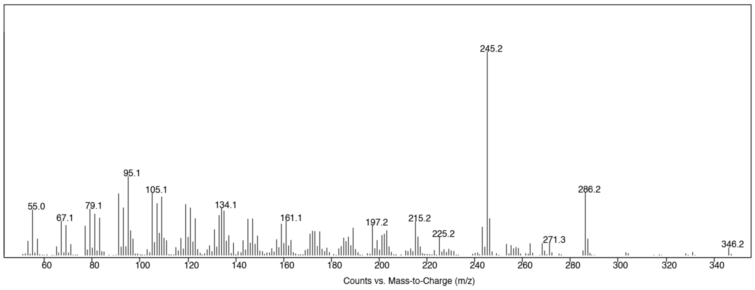
**7**	$C_{20}H_{32}O_2$	304.2402	14.49 (GCMS)	GCMS: 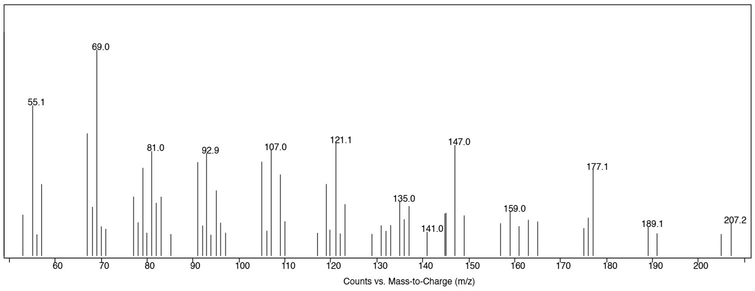

acetylated **7**	$C_{22}H_{34}O_3$	346.2508	14.83 (GCMS)	GCMS: 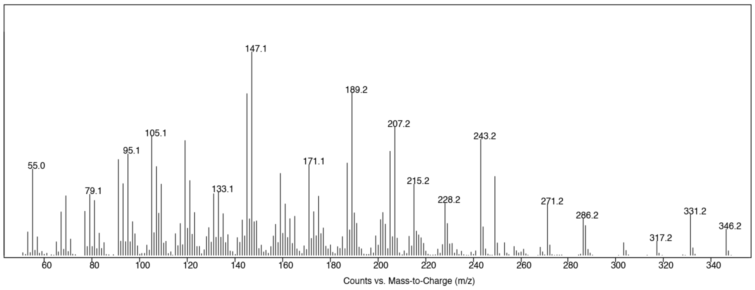
**8**	$C_{20}H_{34}O_2$	306.2559	15.80 (GCMS)	GCMS: 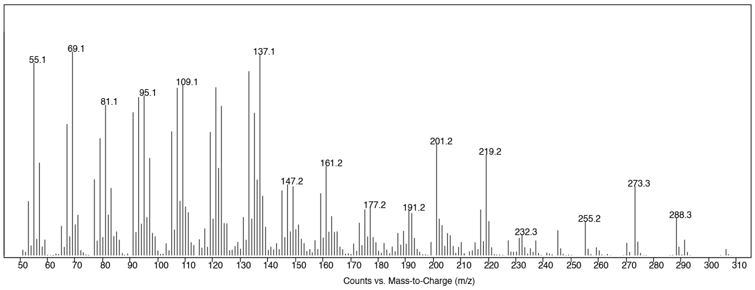
**9**	$C_{20}H_{32}O_2$	304.2402	13.82 (GCMS)	GCMS: 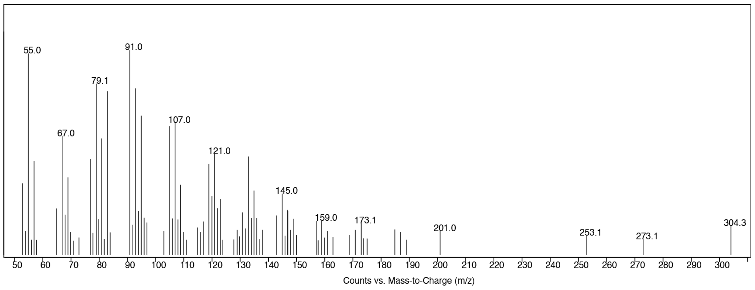
**10** (acetylated **9**)	$C_{22}H_{34}O_3$	346.2508	14.80 (GCMS)	GCMS: 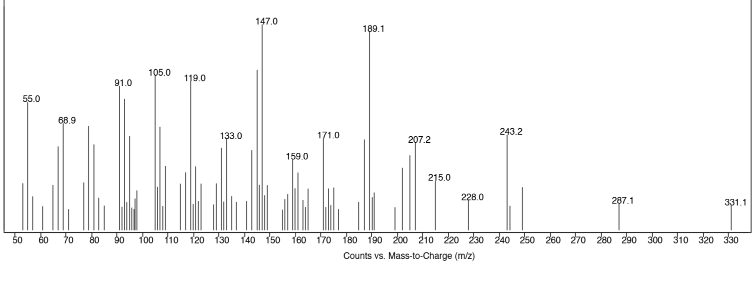

5α-acetoxytaxadiene (**11**)	$C_{22}H_{34}O_2$	330.2559	13.77 (GCMS)	GCMS: 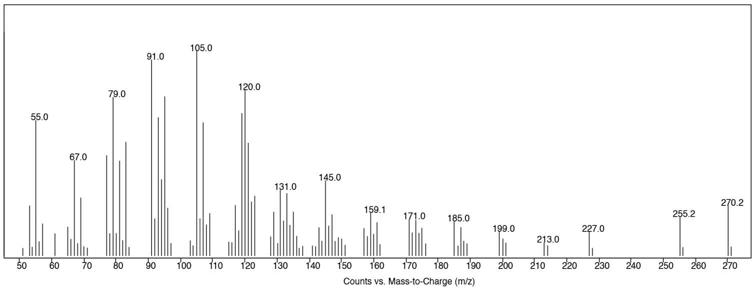
5α-acetoxytaxadien-10β-ol (**12**)	$C_{22}H_{34}O_3$	346.2508	15.79 (GCMS)	GCMS: 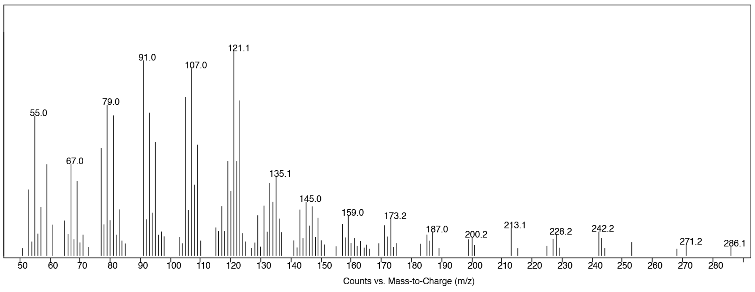
5α,10β-diacetoxytaxadiene (**13**)	$C_{24}H_{36}O_4$	388.2614	16.06 (GCMS)	GCMS: 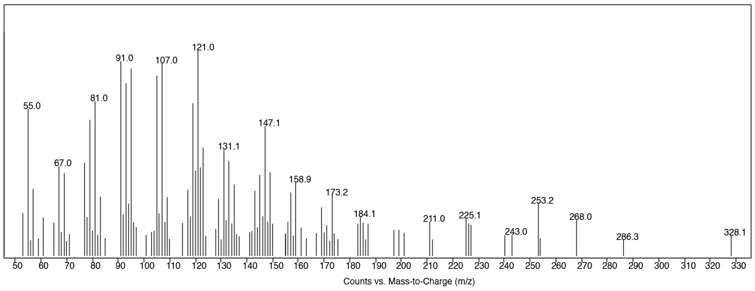

5α,10β-diace toxytaxadien -13α-ol (**14**)	$C_{24}H_{36}O_5$	404.2563	17.91 (GCMS), 8.66 (LCMS)	GCMS:<br>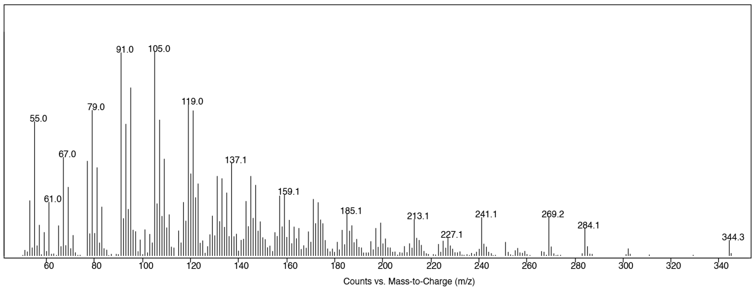<br><br>LCMS:<br>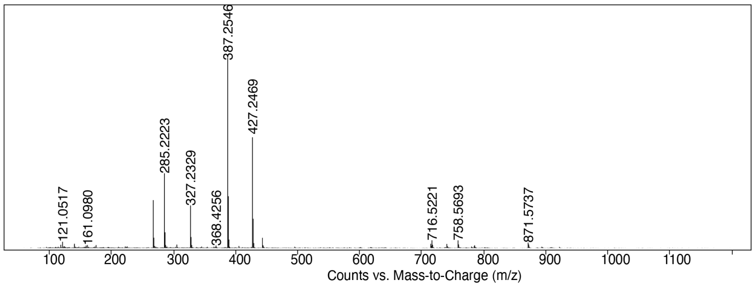
5α,10β-diace toxytaxadien -13-one (**15**)	$C_{24}H_{34}O_5$	402.2406	17.50 (GCMS), 8.32 (LCMS)	GCMS:<br>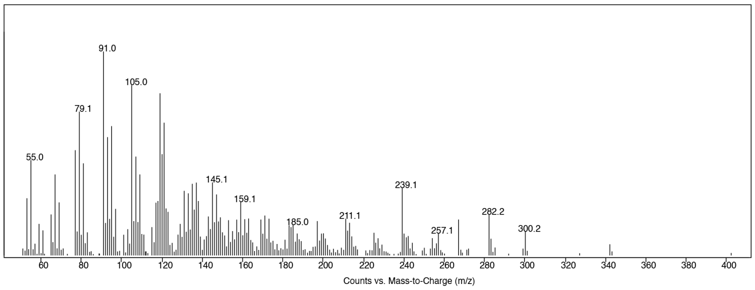<br><br>LCMS:<br>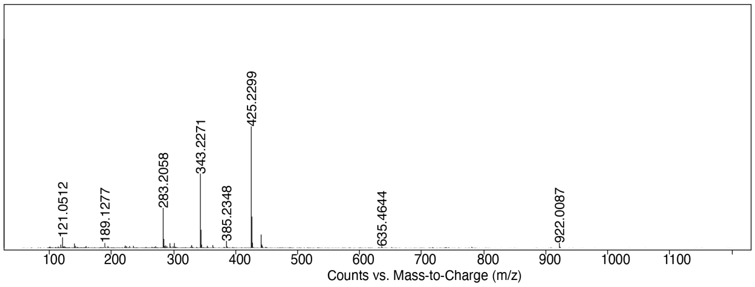

taxadien-5α, 13α-diol (**16**)	$C_{20}H_{32}O_2$	304.2402	10.17 (LCMS)	LCMS: 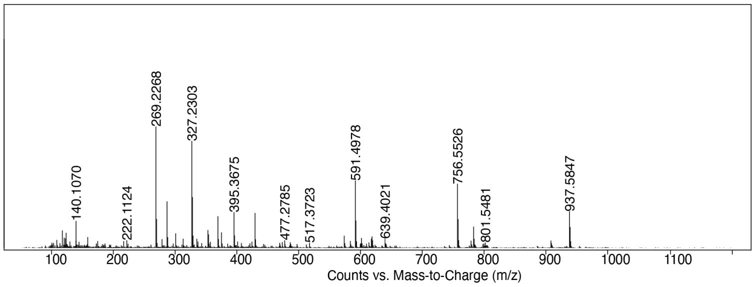
5α-hydroxytaxadien-13-one (**17**)	$C_{20}H_{30}O_2$	302.2246	9.40 (LCMS)	LCMS: 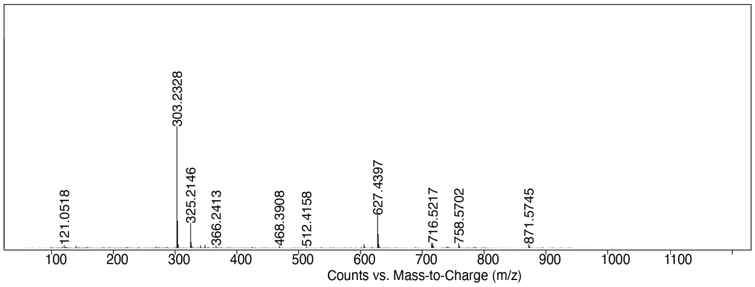
5α-acetoxytaxadien-13α-ol (**18**)	$C_{22}H_{34}O_3$	346.2508	15.83 (GCMS)	GCMS: 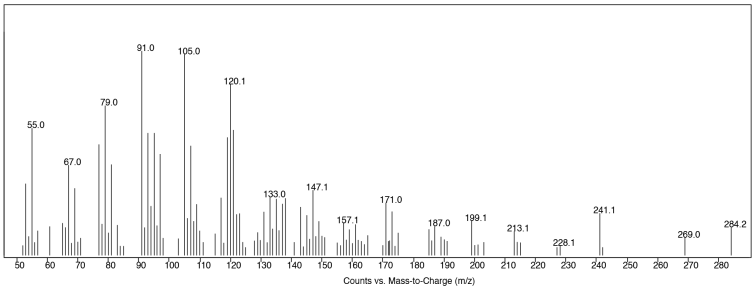
5α-acetoxytaxadien-13-one (**19**)	$C_{22}H_{32}O_3$	344.2351	9.92 (LCMS)	LCMS: 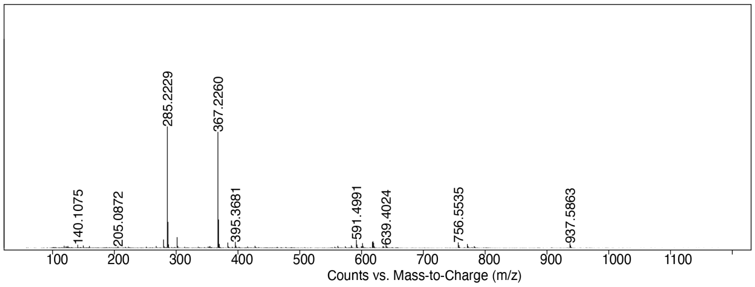

<table>
<tr><td>5α-acetoxytaxadien-10β,13α-diol (20)</td><td>$C_{22}H_{34}O_4$</td><td>362.2457</td><td>17.66 (GCMS)</td><td>GCMS:
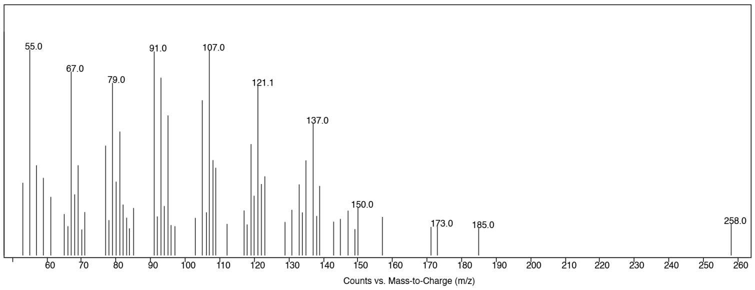
</td></tr>
<tr><td>5α-acetoxy-10β-hydroxytaxadien-13-one (21)</td><td>$C_{22}H_{32}O_4$</td><td>360.2301</td><td>6.69 (LCMS)</td><td>LCMS:
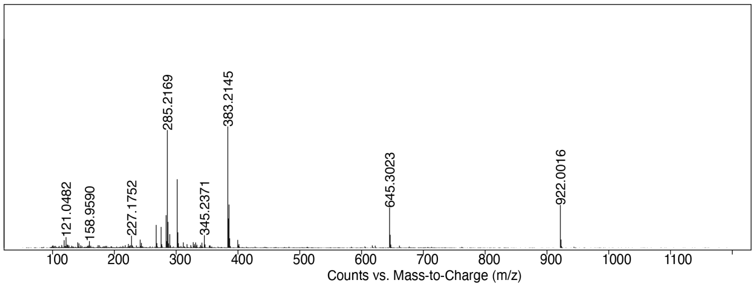
</td></tr>
</table>

Supplementary Table 3. Summary of compound purification in this study.

Name	Source	Scale	Yield (mg)	Column condition	NMR
**1**	Yeast expressing TS	400 mL culture	15.5	20 g silica column (isocratic 100% hexane)	Supplementary Figure 11~13
**2**	synthesis	15.5 mg of **1**	1.8	200 mg silica column (hexane:diethyl ether = 5:1)	Supplementary Figure 14-15
**3**	*N. benthamiana* transiently expressing tHMGR+GGPPS+TS1/2+T5αH+TAX19	58 * 6-weeks old plants (73.47 g DW)	0.4	1. 250 g silica column (EA:Hex = 3:7, 2 L; EA:Hex = 4:6, 1 L; EA:Hex = 1:1, 1L; EA:Hex = 6:4, 1L; EA:Hex = 8:2, 1L) 2. Biotage C18 6 g column (50% 5 CV, 50-65% over 45 CV)	Supplementary Figure 16-17
**5**	*N. benthamiana* transiently expressing tHMGR+GGPPS+TS1/2+(NOS)T5αH+TAT+(NOS)T10βH+DBAT+(NOS)T13αH	26 * 4-weeks old plants (14.31 g DW)	0.4	1. 100 g silica column (EA:Hex = 2:8, 1 L; EA:Hex = 3:7, 1 L; EA:Hex = 1:1, 1 L) 2. Biotage C18 6 g column (50% 3 CV, 50-70% over 20 CV)	Supplementary Figure 18~23 Supplementary Table 3
acetylated **6**	*N. benthamiana* transiently expressing tHMGR+GGPPS+TS1/2+T5αH+TAX19	58 * 6-weeks old plants (73.47 g DW)	1.5	1. 250 g silica column (EA:Hex = 3:7, 2 L; EA:Hex = 4:6, 1 L; EA:Hex = 1:1, 1L; EA:Hex = 6:4, 1L; EA:Hex = 8:2, 1L) 2. Biotage C18 6 g column (50% 5 CV, 50-65% over 45 CV)	Supplementary Figure 24~30 Supplementary Table 4
acetylated **7**	*N. benthamiana* transiently expressing tHMGR+GGPPS+TS1/2+T5αH+TAX19	58 * 6-weeks old plants (73.47 g DW)	1.7	1. 250 g silica column (EA:Hex = 3:7, 2 L; EA:Hex = 4:6, 1 L; EA:Hex = 1:1, 1L; EA:Hex = 6:4, 1L; EA:Hex = 8:2, 1L) 2. Biotage C18 6 g column (50% 5 CV, 50-65% over 45 CV)	Supplementary Figure 31~35 Supplementary Table 5
**8**	*S.cerevisiae* expressing TS+T5αH	4 L culture	1.8	1. 25 g Biotage silica column (100% Hex 10 CV, 100-85% Hex/EA over 15 CV) 2. Biotage C18 12 g column (40% 5 CV, 40-50% over 30 CV)	Supplementary Figure 36~42 Supplementary Table 6

**14**	*N. benthamiana* transiently expressing tHMGR+GGPPS+TS1/2+(NOS)T5αH+TAT+(NOS)T10βH+DBAT+(NOS)T13αH	26 * 4-weeks old plants (14.31 g DW)	0.9	1. 100 g silica column (EA:Hex = 2:8, 1 L; EA:Hex = 3:7, 1 L; EA:Hex = 1:1, 1 L)<br>2. Biotage C18 6 g column (50% 3 CV, 50-70% over 20 CV)	Supplementary Figure 43~48 Supplementary Table 8
**15**	*N. benthamiana* transiently expressing tHMGR+GGPPS+TS1/2+(NOS)T5αH+TAT+(NOS)T10βH+DBAT+(NOS)T13αH	26 * 4-weeks old plants (14.31 g DW)	0.6	1. 100 g silica column (EA:Hex = 2:8, 1 L, EA:Hex = 3:7, 1 L, EA:Hex = 1:1, 1 L)<br>2. Biotage C18 6 g column (50% 3 CV, 50-65% over 15 CV)	Supplementary Figure 49~54 Supplementary Table 8

Solvent system for biotage Sfär C18 D Duo 100 Å 30 μm column: A =water, B = acetonitrile. Percentage of solvent B is listed. TAX19 is a TAT homolog that has 5α-*O*-acetylation activity on taxadien-5α-ol but shows different regioselectivity to TAT on taxusin-tetraol substrate compared to TAT.[18]
DW: dry weight, EA: ethyl acetate, Hex: hexane, CV: column volume.

Supplementary Table 4. ^{13}C & ^{1}H δ assignments of mono-oxidized taxadiene 5.

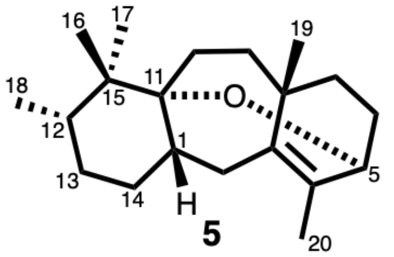

Carbon	δ ^{13}C (ppm)	δ ^{1}H (mult.; *J* in Hz)
1	42.86	1.97 (dt; 6.0, 5.5)
2	27.19	2.23 (dd; 6.0, 13.6),<br>2.42 (d; 13.6)
3	140.7	-
4	131.7	-
5	70.2	3.80 (d; 4.3)
6	29.21	1.77 (ddt; 3.5, 4.3, 14.4),<br>2.08 (tt; 4.3, 14.4)
7	31.95	1.10 (dt; 14.6, 3.5),<br>1.86 (dt; 13.6, 3.5)
8	38.70	-
9	35.41	1.12 (dt; 12.9, 3.5), 2.18 (m)
10	24.51	1.49 (m)
11	77.14	-
12	37.56	1.43 (m)
13	30.34	1.27 (m), 1.33 (m)
14	27.73	1.40 (m), 1.54 (m)
15	42.88	-
16	16.16	0.86 (s)
17	23.36	0.84 (s)
18	16.32	0.78 (d; 6.7)
19	28.22	0.93 (s)

20	19.95	1.74 (s)

s = singlet, d = doublet, dd = doublet of doublets, dt = doublet of triplets, t = triplet, q = quartet, quint = quintet, m = multiplet

Supplementary Table 5. ^{13}C & ^{1}H δ assignments of acetylated 6

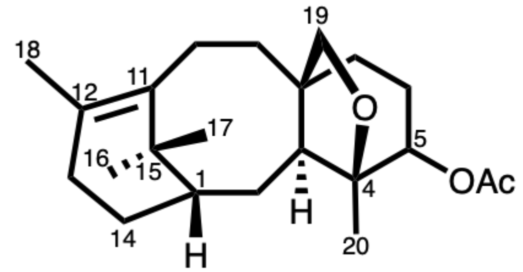

acetylated **6**

Carbon	δ ^{13}C (ppm)	δ ^{1}H (mult.; *J* in Hz)
1	43.05	1.78 (m)
2	27.29	1.49 (m)
3	42.16	2.25 (dd; 2.2, 7.6)
4	86.1	-
5	77.75b	4.68 (m)
6	25.18	1.63 (m), 1.99a (m)
7	32.54	1.19, 1.99b (m)
8	47.26	-
9	33.42	1.27 (m), 2.00 (m)
10	24.03	2.09 (d; 3.5),<br>2.61 (dt; 5.3, 13.4)
11	136.40	-
12	129.83	-
13	29.97	1.96 (m), 2.37 (m)
14	23.02	1.36 (m), 2.15 (m)
15	38.63	-
16	30.83	1.05 (s)
17	25.26	1.30 (s)
18	21.75	1.76 (s)
19	77.75a	3.56 (dd; 1.7, 8.0),<br>3.61 (d; 8.0)

20	21.43	1.13 (s)
$-\underline{C}O_2Me$	169.80	-
$-CO_2\underline{Me}$	21.36	2.07

Supplementary Table 6. ^{13}C & ^{1}H δ assignments of acetylated 7 in this study and OCT (3) from reference[11] as a comparison.

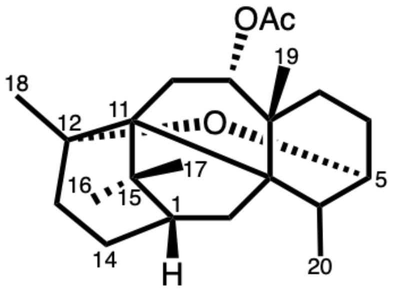

acetylated **7**

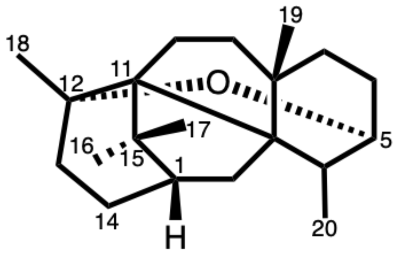

OCT (**3**)

	acetylated **7**		OCT (**3**)	
Carbon	δ ^{13}C (ppm)	δ ^{1}H (mult.; *J* in Hz)	δ ^{13}C (ppm)[11]	δ ^{1}H (mult.; *J* in Hz)[11]
1	44.58	1.74 (m)	45.9	1.71 (dd; 5.0, 8.4)
2	N.A.	1.41 (m), 2.24 (dd; 5.2, 13.0)	39.1	1.33 (d; 12.9), 2.21 (dd; 5.0, 12.9)
3	51.12	-	53.3	-
4	N.A.	2.51 (qd; 7.0, 3.5)	37.1	2.47 (qd; 7.0, 3.4)
5	69.45	4.01 (dd; 3.6, 8.7)	69.8	3.97 (dd; 3.4, 9.1)
6	N.A.	2.06 (m)	30.2	1.83 (m), 2.04 (m)
7	N.A.	N.A.	37.5	1.36 (d; 10.9), 1.83 (m)
8	43.78	-	42.7	-
9	82.18	4.98 (dd; 7.5, 11.5)	47.3	1.53 (dd; 8.8, 12.7), 1.82 (m)
10	N.A.	1.82 (dd; 7.9, 12.4), 1.93 (t, 12.0)	30.2	1.31 (dd; 3.6, 9.6), 1.38 (ddd; 1.0, 3.6, 10.9)
11	60.00	-	66.0	-
12	79.45	-	80.5	-
13	N.A.	N.A.	36.4	1.84 (m), 1.98 (m)
14	N.A.	N.A.	28.1	1.62 (ddd; 5.2, 11.2, 14.3), 2.01 (m)
15	N.A.	-	46.0	-
16	29.22	1.03 (s)	28.6	0.93 (brs)

17	26.83	1.07 (s)	26.9	1.01 (brs)
18	30.15	1.19 (s)	30.3	1.19 (s)
19	26.34	1.02 (s)	28.0	1.04 (s)
20	15.28	1.19 (d; 7.0)	15.2	1.13 (d; 7.0)
-<u>C</u>O_2Me	170.81	-	-	-
-CO_2<u>Me</u>	21.46	2.07	-	-

* N.A.: chemical shift not assigned due to insufficient information

Supplementary Table 7. ^{13}C & ^{1}H δ assignments of 8.

	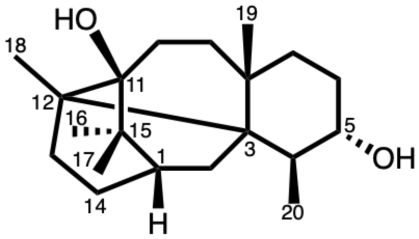 **8**	
Carbon	δ ^{13}C (ppm)	δ ^{1}H (mult.; *J* in Hz)
1	39.51	1.22b (m)
2	30.09	1.63 (m), 1.82 (dd; 4.0, 15.1)
3	45.77	-
4	40.66	1.74 (m)
5	73.94	3.87 (ddd; 6.8, 9.1, 12.5)
6	28.65	1.65 (m), 1.96 (dddd; 2.0, 6.8, 11.7, 13.8)
7	32.98	1.34 (m), 2.06 (m)
8	37.17	-
9	37.98	1.17 (m), 1.66 (m)
10	30.55	1.66 (m)
11	76.20	-
12	43.68	-
13	26.32	1.36 (m), 1.57 (m)
14	22.99	1.28 (dq, 2.2, 10.7), 1.74 (m)
15	N.A.	-
16	24.36	1.14 (s)
17	27.24	1.06 (s)
18	19.22	1.03 (s)
19	31.38	0.90 (d; 0.87)
20	20.40	1.22 (d; 7.1)

Supplementary Table 8. ^{13}C & ^{1}H δ assignments of 5α,10β-diacetoxy-13α-ol (14) and 5α,10β-diacetoxy-13α-one (15).

	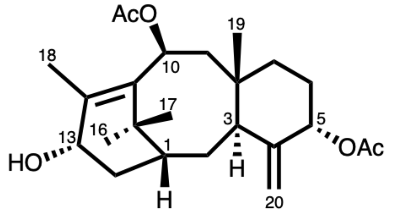<br>taxadien-5α,10β-diacetoxy-13α-ol (**14**)		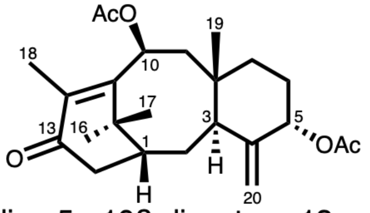<br>taxadien-5α,10β-diacetoxy-13-one (**15**)	
Carbon	δ ^{13}C (ppm)	δ ^{1}H (mult.; *J* in Hz)	δ ^{13}C (ppm)	δ ^{1}H (mult.; *J* in Hz)
1	40.42	1.18 (dd; 4.8, 15.4)	41.65	2.96 (m)
2	28.10	1.68 (t; 6.2),<br>2.86 (dt; 15.2, 9.7)	26.92	1.94 (d; 19.6),<br>2.91 (dd; 7.3, 19.6)
3	37.11	3.04 (m)	36.11	2.15 (m)
4	N.A.	-	N.A.	-
5	76.96	5.34 (s)	75.75	5.29 (s)
6	28.25	1.79 (m), 1.86 (m)	28.18	1.79 (m)
7	34.10	1.25 (m), 2.02 (m)	33.28	1.27 (m), 1.99 (m)
8	38.48	-	38.45	-
9	44.22	1.59 (dd; 5.5, 14.8),<br>2.32 (t; 13.4)	43.53	1.70 (dd; 5.5, 14.5),<br>2.43 (t; 13.4)
10	71.39	6.11 (dd; 5.5, 12.3)	71.66	6.13 (dd; 5.6, 12.2)
11	137.27	-	155.30	-
12	137.91	-	136.10	-
13	68.40	4.41 (m)	200.47	-
14	36.78	1.73 (d; 9.6)	36.11	1.28 (m), 1.79 (m)
15	38.98	-	39.92	-
16	32.47	0.95 (s)	37.31	1.13 (s)
17	25.90	1.48 (s)	25.05	1.57 (s)
18	22.02	2.18 (s)	21.55	1.95 (s)
19	21.63	0.72 (s)	22.13	0.75 (s)

20	113.26	4.83 (s), 5.15 (s)	112.45	4.78 (s), 5.14 (s)
-<u>C</u>O_2Me	170.36	-	169.85	-
-<u>C</u>O_2Me	170.36	-	170.04	-
-CO_2<u>Me</u>	21.50	2.05 (s)	21.43	2.09 (s)
-CO_2<u>Me</u>	15.95	2.10 (s)	13.66	2.18 (s)

* N.A.: chemical shift not assigned due to insufficient information

Supplementary Table 9. Accession numbers of genes used in this study.

Name	Organism	Accession number
TS1	*Taxus brevefolia*	U48796
TS2	*Taxus canadensis*	AY364470
T5αH	*Taxus cuspidata*	AY289209
TAT	*Taxus cuspidata*	AF190130
T10βH	*Taxus cuspidata*	AF318211
DBAT	*Taxus baccata*	AF456342
T13αH	*Taxus cuspidata*	AY056019

Supplementary Table 10. List of PCR primer pairs used in this study.

Purpose	Target		Sequence (5' to 3')
Cloning	T5αH	F	ATTCTGCCCAAATTCGCGACCGGT
Cloning	T5αH	R	GAAACCAGAGTTAAAGGCCTCGAGCTATGGTCTCGGAAACAGTTTAAT
Cloning	TAT	F	ATTCTGCCCAAATTCGCGACCGGT
Cloning	TAT	R	GAAACCAGAGTTAAAGGCCTCGAGTCATACTTTAGCCACATATTTTTT
Cloning	T10βH	F	ATTCTGCCCAAATTCGCGACCGGT
Cloning	T10βH	R	GAAACCAGAGTTAAAGGCCTCGAGTTAGGATCTCGGAAAAAGTTTTAT
Cloning	DBAT	F	ATTCTGCCCAAATTCGCGACCGGT
Cloning	DBAT	R	GAAACCAGAGTTAAAGGCCTCGAGTCAAGGTTTAGTTACATATTTGTT
Cloning	T13αH	F	ATTCTGCCCAAATTCGCGACCGGT
Cloning	T13αH	R	GAAACCAGAGTTAAAGGCCTCGAGTTAAGATCTGGAATAGAGTTTAAT
Promoter switching	To replace 35S with NOS in the pEAQ	F	CAATTAGAGTCTCATATTCACTCTCAATTATTAAAATCTTAATAGGTTTTGATAAAAGCG
Promoter switching	To replace 35S with NOS in the pEAQ	R	CTAAAGAAAATTTAATGAAACCAGAGTTAAACCGGTCGCGAATTTGGGCAGAATATACAG
Promoter switching	To replace 35S with UBQ10 in the pEAQ	F	ATTAATCTGAGTTTTTCTGATTAACACTTATTAAAATCTTAATAGGTTTTGATAAAAGCG
Promoter switching	To replace 35S with UBQ10 in the pEAQ	R	CTAAAGAAAATTTAATGAAACCAGAGTTAAACCGGTCGCGAATTTGGGCAGAATATACAG
Promoter switching	To PCR any gene from pEAQ vector into either pEAQ-NOS or pEAQ-UBQ10	F	CAATTAGAGTCTCATATTCACTCTCAATTATTAAAATCTTAATAGGTTTTGATAAAAGCG
Promoter switching	To PCR any gene from pEAQ vector into either pEAQ-NOS or pEAQ-UBQ10	R	GTAAATTCAAACTAAAGAAAATTTAATGAAACCAGAGTTAA

Nucleotides underlined are overlaps designed for Gibson assembly into pEAQ vectors. Synthetic genes contain 5'-overlaps designed for Gibson assembly into pEAQ vectors thus the same forward primer is used for PCR.

Supplementary Table 11. List of *S. cerevisiae* strains used in this study.

Strain ID	Name/alias	Description	Genotype
JBEI-18127	TS-expressing/ 5xTS	Mevalonate pathway, 3x CrtE GGPP synthases, 5x TS with protein tags, all in galactose-inducible promoters.	CEN.PK2-1C {1114a,1622b,308a,911b::GAL1p-MBP-TXS-ERG20-TDH1t; 1014a::GAL1p-TXS-GFP-ADH1t, leu2-3, 112::HIS3MX6-GAL1p-ERG19/GAL10p-ERG8; ura3-52::ura3/GAL1p-MvaSA110G/GAL10p-MvaE; his3Δ1::hphMX4-GAL1p-ERG12/GAL10p-IDI1; trp1-289::TRP1/GAL1p-CrtE/GAL10p-ERG20; YPRCdelta15::NatMX-GAL1p-CrtE/GAL10p-CrtE; MATa}
JBEI-18128	TS+T5αH-expressing/ 3xTS {T5αH-CPR}	Mevalonate pathway, 3x CrtE GGPP synthases, 3x TS with protein tags, and 1x T5αH-CPR, all in galactose-inducible promoters.	CEN.PK2-1C {511b::GAL1p-T5OH-PGK1t/GAL3p-CPR-ENO2t; 1114a,1622b::GAL1p-MBP-TXS-ERG20-TDH1t; 1014a::GAL1p-TXS-GFP-ADH1t, leu2-3, 112::HIS3MX6-GAL1p-ERG19/GAL10p-ERG8; ura3-52::ura3/GAL1p-MvaSA110G/GAL10p-MvaE; his3Δ1::hphMX4-GAL1p-ERG12/GAL10p-IDI1; trp1-289::TRP1/GAL1p-CrtE/GAL10p-ERG20; YPRCdelta15::NatMX-GAL1p-CrtE/GAL10p-CrtE; MATa}

Methods used to construct these strains are previously described.[19]

Supplementary References

1. Chau, M., Jennewein, S., Walker, K. & Croteau, R. Taxol Biosynthesis Molecular Cloning and Characterization of a Cytochrome P450 Taxoid 7β-Hydroxylase. *Chemistry & Biology* **11**, 663–672 (2004).
2. Walker, K. & Croteau, R. Taxol biosynthesis: molecular cloning of a benzoyl-CoA:taxane 2alpha-O-benzoyltransferase cDNA from taxus and functional expression in Escherichia coli. *Proc. Natl. Acad. Sci. U. S. A.* **97**, 13591–13596 (2000).
3. Sainsbury, F., Thuenemann, E. C. & Lomonossoff, G. P. pEAQ: versatile expression vectors for easy and quick transient expression of heterologous proteins in plants. *Plant Biotechnol. J.* **7**, 682–693 (2009).
4. De La Peña, R. & Sattely, E. S. Rerouting plant terpene biosynthesis enables momilactone pathway elucidation. *Nat. Chem. Biol.* **17**, 205–212 (2021).
5. Sagwan-Barkdoll, L. & Anterola, A. M. Taxadiene-5α-ol is a minor product of CYP725A4 when expressed in Escherichia coli. *Biotechnol. Appl. Biochem.* **65**, 294–305 (2018).
6. Barton, N. A. *et al.* Accessing low-oxidation state taxanes: is taxadiene-4(5)-epoxide on the taxol biosynthetic pathway? *Chem. Sci.* **7**, 3102–3107 (2016).
7. Biggs, B. W. *et al.* Orthogonal Assays Clarify the Oxidative Biochemistry of Taxol P450 CYP725A4. *ACS Chem. Biol.* **11**, 1445–1451 (2016).
8. Edgar, S. *et al.* Mechanistic Insights into Taxadiene Epoxidation by Taxadiene-5α-Hydroxylase. *ACS Chem. Biol.* **11**, 460–469 (2016).
9. Yadav, V. G. Unraveling the multispecificity and catalytic promiscuity of taxadiene monooxygenase. *J. Mol. Catal. B Enzym.* **110**, 154–164 (2014).
10. Jennewein, S., Long, R. M., Williams, R. M. & Croteau, R. Cytochrome p450 taxadiene 5alpha-hydroxylase, a mechanistically unusual monooxygenase catalyzing the first oxygenation step of taxol biosynthesis. *Chem. Biol.* **11**, 379–387 (2004).

11. Rontein, D. *et al.* CYP725A4 from yew catalyzes complex structural rearrangement of taxa-4 (5), 11 (12)-diene into the cyclic ether 5 (12)-oxa-3 (11)-cyclotaxane. *J. Biol. Chem.* **283**, 6067–6075 (2008).

12. Ajikumar, P. K. *et al.* Isoprenoid pathway optimization for Taxol precursor overproduction in Escherichia coli. *Science* **330**, 70–74 (2010).

13. Zhou, K., Qiao, K., Edgar, S. & Stephanopoulos, G. Distributing a metabolic pathway among a microbial consortium enhances production of natural products. *Nat. Biotechnol.* **33**, 377–383 (2015).

14. Li, J. *et al.* Chloroplastic metabolic engineering coupled with isoprenoid pool enhancement for committed taxanes biosynthesis in Nicotiana benthamiana. *Nat. Commun.* **10**, 4850 (2019).

15. Walls, L. E. *et al.* Optimizing the biosynthesis of oxygenated and acetylated Taxol precursors in Saccharomyces cerevisiae using advanced bioprocessing strategies. *Biotechnol. Bioeng.* **118**, 279–293 (2021).

16. Xiong, X. *et al.* The Taxus genome provides insights into paclitaxel biosynthesis. *Nat Plants* **7**, 1026–1036 (2021).

17. Nowrouzi, B., Lungang, L. & Rios-Solis, L. Exploring optimal Taxol® CYP725A4 activity in Saccharomyces cerevisiae. *Microb. Cell Fact.* **21**, 197 (2022).

18. Chau, M., Walker, K., Long, R. & Croteau, R. Regioselectivity of taxoid-O-acetyltransferases: heterologous expression and characterization of a new taxadien-5α-ol-O-acetyltransferase. *Arch. Biochem. Biophys.* **430**, 237–246 (2004).

19. Reider Apel, A. *et al.* A Cas9-based toolkit to program gene expression in Saccharomyces cerevisiae. *Nucleic Acids Res.* **45**, 496–508 (2017).
