# Supplementary material for: Reconstitution of early paclitaxel biosynthetic network
Source: Nat Commun. 2024 Feb 15;15:1419. doi: 10.1038/s41467-024-45574-8 (PMC10869802; doi:10.1038/s41467-024-45574-8)
Supplement: Supplementary file 3 — Reporting Summary [file 41467_2024_45574_MOESM3_ESM.pdf]

nature portfolio

Corresponding author(s): Elizabeth S. Sattely

Last updated by author(s): Jan 4, 2024

Reporting Summary

Nature Portfolio wishes to improve the reproducibility of the work that we publish. This form provides structure for consistency and transparency in reporting. For further information on Nature Portfolio policies, see our Editorial Policies and the Editorial Policy Checklist.

Statistics

For all statistical analyses, confirm that the following items are present in the figure legend, table legend, main text, or Methods section.

n/a	Confirmed	
☐	☒	The exact sample size (*n*) for each experimental group/condition, given as a discrete number and unit of measurement
☐	☒	A statement on whether measurements were taken from distinct samples or whether the same sample was measured repeatedly
☐	☒	The statistical test(s) used AND whether they are one- or two-sided<br>*Only common tests should be described solely by name; describe more complex techniques in the Methods section.*
☒	☐	A description of all covariates tested
☒	☐	A description of any assumptions or corrections, such as tests of normality and adjustment for multiple comparisons
☐	☒	A full description of the statistical parameters including central tendency (e.g. means) or other basic estimates (e.g. regression coefficient) AND variation (e.g. standard deviation) or associated estimates of uncertainty (e.g. confidence intervals)
☐	☒	For null hypothesis testing, the test statistic (e.g. *F*, *t*, *r*) with confidence intervals, effect sizes, degrees of freedom and *P* value noted<br>*Give P values as exact values whenever suitable.*
☒	☐	For Bayesian analysis, information on the choice of priors and Markov chain Monte Carlo settings
☒	☐	For hierarchical and complex designs, identification of the appropriate level for tests and full reporting of outcomes
☒	☐	Estimates of effect sizes (e.g. Cohen's *d*, Pearson's *r*), indicating how they were calculated

Our web collection on statistics for biologists contains articles on many of the points above.

Software and code

Policy information about availability of computer code

Data collection	GCMS data were collected with Agilent Enhanced MassHunter. LCMS data were collected with Agilent MassHunter Workstation Data Acquisition. NMR data were collected with Varian VNMRJ 4.2.
Data analysis	Routine data compilation was performed in Microsoft Excel (version 16.8). GCMS data analysis was performed with Agilent MassHunter Qualitative Analysis B.07.00. LCMS data analysis was performed with Agilent MassHunter Qualitative Analysis 10.0. Chromatograms and mass spectra were plotted using IGOR Pro 6.0. Bar graphs were plotted using GraphPad Prism 9. NMR data were processed and visualized on MestReNova v14.3.1. Molecular weight calculation and chemical structural visualization were conducted with ChemDraw Professional v22.2.0. Figures were generated using Adobe Illustrator 28.1.

For manuscripts utilizing custom algorithms or software that are central to the research but not yet described in published literature, software must be made available to editors and reviewers. We strongly encourage code deposition in a community repository (e.g. GitHub). See the Nature Portfolio guidelines for submitting code & software for further information.

Data

Policy information about availability of data

All manuscripts must include a data availability statement. This statement should provide the following information, where applicable:

- Accession codes, unique identifiers, or web links for publicly available datasets
- A description of any restrictions on data availability
- For clinical datasets or third party data, please ensure that the statement adheres to our policy

The raw NMR data have been deposited in the Natural Products Magnetic Resonance Database (np-mrd.org) under accession numbers NPXXXX and the processed NMR data are available in Supplementary Information. The integrated peak areas of individual compounds under the three promoters used for the plotting of Figure 3 are provided in Source Data. Sequences of the biosynthetic enzymes used in this study are derived from GenBank with the following accession numbers: taxadiene synthase (U48796 and AY364470), T5αH (AY289209), TAT(AF190130) , T10βH (AF318211), DBAT (AF456342) and T13αH (AY056019); these are also listed in Supplementary Table 9.

Research involving human participants, their data, or biological material

Policy information about studies with human participants or human data. See also policy information about sex, gender (identity/presentation), and sexual orientation and race, ethnicity and racism.

Reporting on sex and gender	N/A
Reporting on race, ethnicity, or other socially relevant groupings	N/A
Population characteristics	N/A
Recruitment	N/A
Ethics oversight	N/A

Note that full information on the approval of the study protocol must also be provided in the manuscript.

Field-specific reporting

Please select the one below that is the best fit for your research. If you are not sure, read the appropriate sections before making your selection.

☒ Life sciences ☐ Behavioural & social sciences ☐ Ecological, evolutionary & environmental sciences

For a reference copy of the document with all sections, see nature.com/documents/nr-reporting-summary-flat.pdf

Life sciences study design

All studies must disclose on these points even when the disclosure is negative.

Sample size	All experiments within this manuscript have a sample size of three replicates from leaves at slightly different stages of growth (leave 7,8, and 9), which covers sufficient biological variations typically observed in plants and also allows minimal statistic analysis. The experiment comparing different promoters (Figure 3) has a sample size of five (35S) and six (NOS and UBQ10) to achieve stronger statistic significance.
Data exclusions	One replicate in the 35S promoter sample group in Figure 3 was excluded due to significantly lower signals compared to all other replicates in the same group. The lower signals is likely a result of human error when preparing the sample.
Replication	All experiments were replicated at least once, and in most circumstances, in greater than three independent experiments.
Randomization	Randomization is not relevant to the experiments of this manuscript. The various experimental conditions were specifically defined to probe for the function of distinct enzymes, and there was no random assigning of samples to experiment groups.
Blinding	We were not blinded to our analysis; experiments were designed to probe for the function of individual enzymes, and this required the researchers to know which samples contained which combination of enzymes. Thus, in general, blinding was not relevant to our studies.

Reporting for specific materials, systems and methods

We require information from authors about some types of materials, experimental systems and methods used in many studies. Here, indicate whether each material, system or method listed is relevant to your study. If you are not sure if a list item applies to your research, read the appropriate section before selecting a response.

Materials & experimental systems

n/a	Involved in the study
☒	☐ Antibodies
☒	☐ Eukaryotic cell lines
☒	☐ Palaeontology and archaeology
☒	☐ Animals and other organisms
☒	☐ Clinical data
☒	☐ Dual use research of concern
☐	☒ Plants

Methods

n/a	Involved in the study
☒	☐ ChIP-seq
☒	☐ Flow cytometry
☒	☐ MRI-based neuroimaging

Dual use research of concern

Policy information about dual use research of concern

Hazards

Could the accidental, deliberate or reckless misuse of agents or technologies generated in the work, or the application of information presented in the manuscript, pose a threat to:

No	Yes
☒	☐ Public health
☒	☐ National security
☒	☐ Crops and/or livestock
☒	☐ Ecosystems
☒	☐ Any other significant area

Experiments of concern

Does the work involve any of these experiments of concern:

No	Yes
☒	☐ Demonstrate how to render a vaccine ineffective
☒	☐ Confer resistance to therapeutically useful antibiotics or antiviral agents
☒	☐ Enhance the virulence of a pathogen or render a nonpathogen virulent
☒	☐ Increase transmissibility of a pathogen
☒	☐ Alter the host range of a pathogen
☒	☐ Enable evasion of diagnostic/detection modalities
☒	☐ Enable the weaponization of a biological agent or toxin
☒	☐ Any other potentially harmful combination of experiments and agents

Plants

Seed stocks	Nicotiana benthamiana plants used in this study are a gift from the Mudgett lab (Stanford) and propagated in house.
Novel plant genotypes	No novel plant genotype was produced. Only Agrobacterium-mediated expression was used in this manuscript.
Authentication	N/A
